# Supplementary material for: Annexin A5 controls VDAC1-dependent mitochondrial Ca2+ homeostasis and determines cellular susceptibility to apoptosis
Source: EMBO J. 2025 May 9;44(12):3413–47. doi: 10.1038/s44318-025-00454-9 (PMC12170872; doi:10.1038/s44318-025-00454-9)
Supplement: Supplementary file 1 — Appendix [file 44318_2025_454_MOESM1_ESM.pdf]

Appendix for Annexin A5 controls VDAC1-dependent mitochondrial Ca2+ homeostasis and determines cellular susceptibility to apoptosis

Table of Contents

Appendix Figure S1. AnxA5 depletion does not affect mitochondria-ER contact sites............3

Appendix Figure S2. AnxA5 localizes near VDAC1, and ER Ca^{2+} release induces its accumulation on the OMM 4

Appendix Figure S3. AnxA5-KO cells are more susceptible to cisplatin-induced apoptosis ...7

Appendix Fig. S1

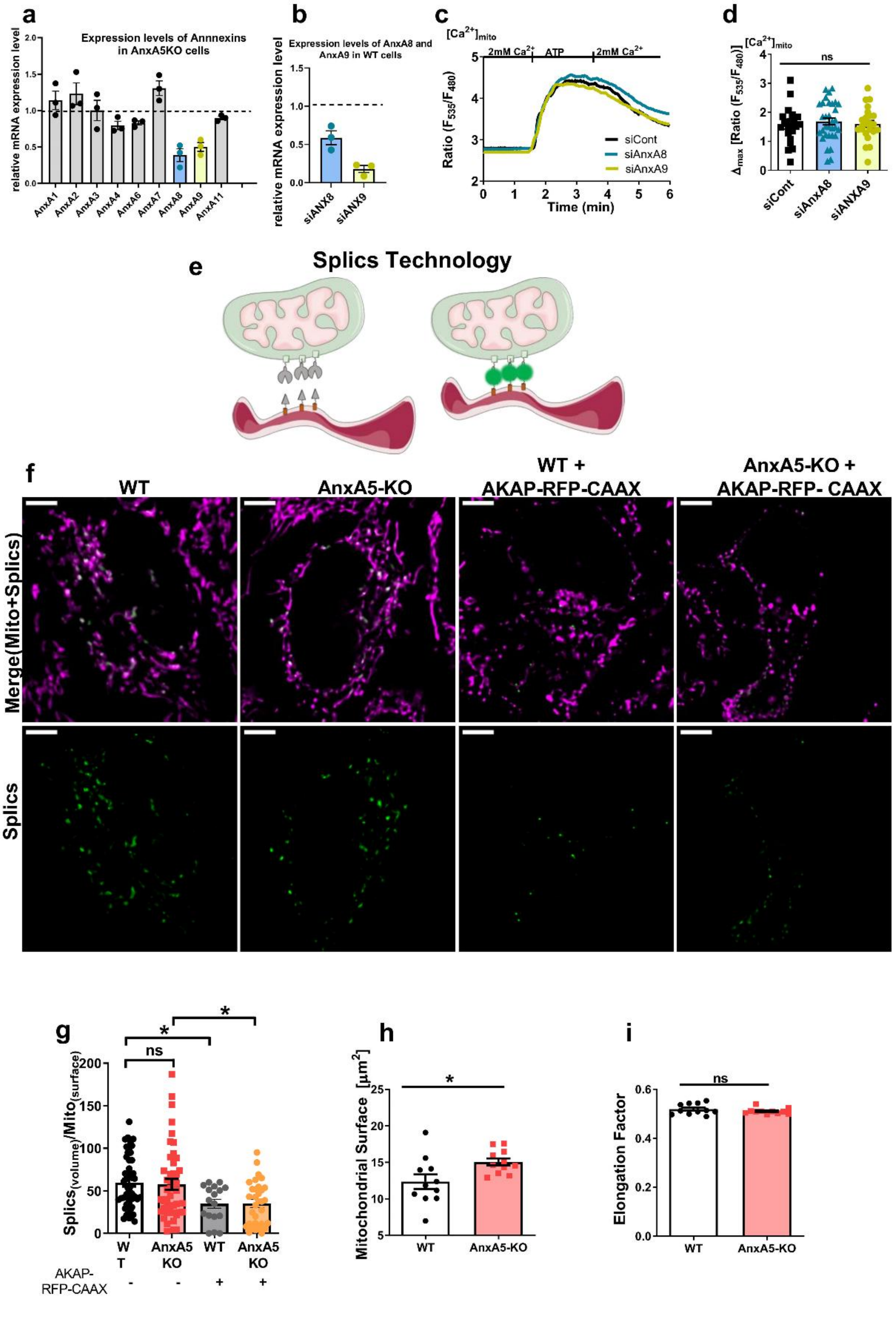

Appendix Figure S1. AnxA5 depletion does not affect mitochondria-ER contact sites.
a Bar graphs show the mRNA expression level of the Annexin family (AnxA1, AnxA2, AnxA3, AnxA4, AnxA6, AnxA7, AnxA8, AnxA9, and AnxA11) in AnxA5-KO cells. **b** Bar graphs show the Knock-down efficiency of AnxA8 and AnxA9 in HeLa cells. Data points represent the mean ± SEM (n=3) **c** Average time courses of the 100 µM ATP-induced $[Ca^{2+}]_{Matrix}$ responses in WT (black), si-AnxA8 (light blue), and si-AnxA9 (light yellow) in HeLa cells measured in Ca^{2+} (2 mM) containing buffer. **d** Bar graphs show the ATP-induced maximum $[Ca^{2+}]_{Matrix}$ level in WT (black), si-AnxA8 (light blue), and si-AnxA9 (light yellow) in HeLa cells. Data points represent the mean ± SEM (nWT = 22/6; n si-AnxA8 = 32/8; nsi-AnxA9 = 23/6). **e** The graphical illustration of the SPLICS sensor. **f** Representative confocal images of WT and AnxA5-KO HeLa cells were captured, stained with MTR-CMX (magenta), and expressing ER-mitochondria contact site sensor SPLICS (green) alone or together with AKAP-RFP-CAAX (tags mitochondria to sub-plasma membrane region). **g** Bar graphs show the ratio of SPLICS volume to mitochondrial surface area in WT (black), AnxA5-KO (red), WT + AKAP-RFP-CAAX (gray), and AnxA5-KO + AKAP-RFP-CAAX (orange) in HeLa cells. Data points represent the mean ± SEM (nWT = 49/4; nAnxA5-KO = 45/4; nWT+ AKAP-RFP-CAAX = 17/5; nAnxA5-KO+AKAP-RFP-CAAX = 32/5). **h** Bar graphs show the mitochondrial surface area [μm^2] and **i** elongation factor in WT (black) and AnxA5-KO (red) HeLa cells. Data points represent the mean ± SEM (n_{WT} =91/11; $n_{AnxA5\text{-}KO}$ =88/11). Significant differences were assessed using either one-way ANOVA with Tukey's multiple comparison tests or Kruskal–Wallis test (* $p < 0.05$, and ns: not significant) and with the unpaired Student's t-test or Kolmogorov-Smirnov test (* $p < 0.05$, and ns: not significant).

Appendix Fig. S2

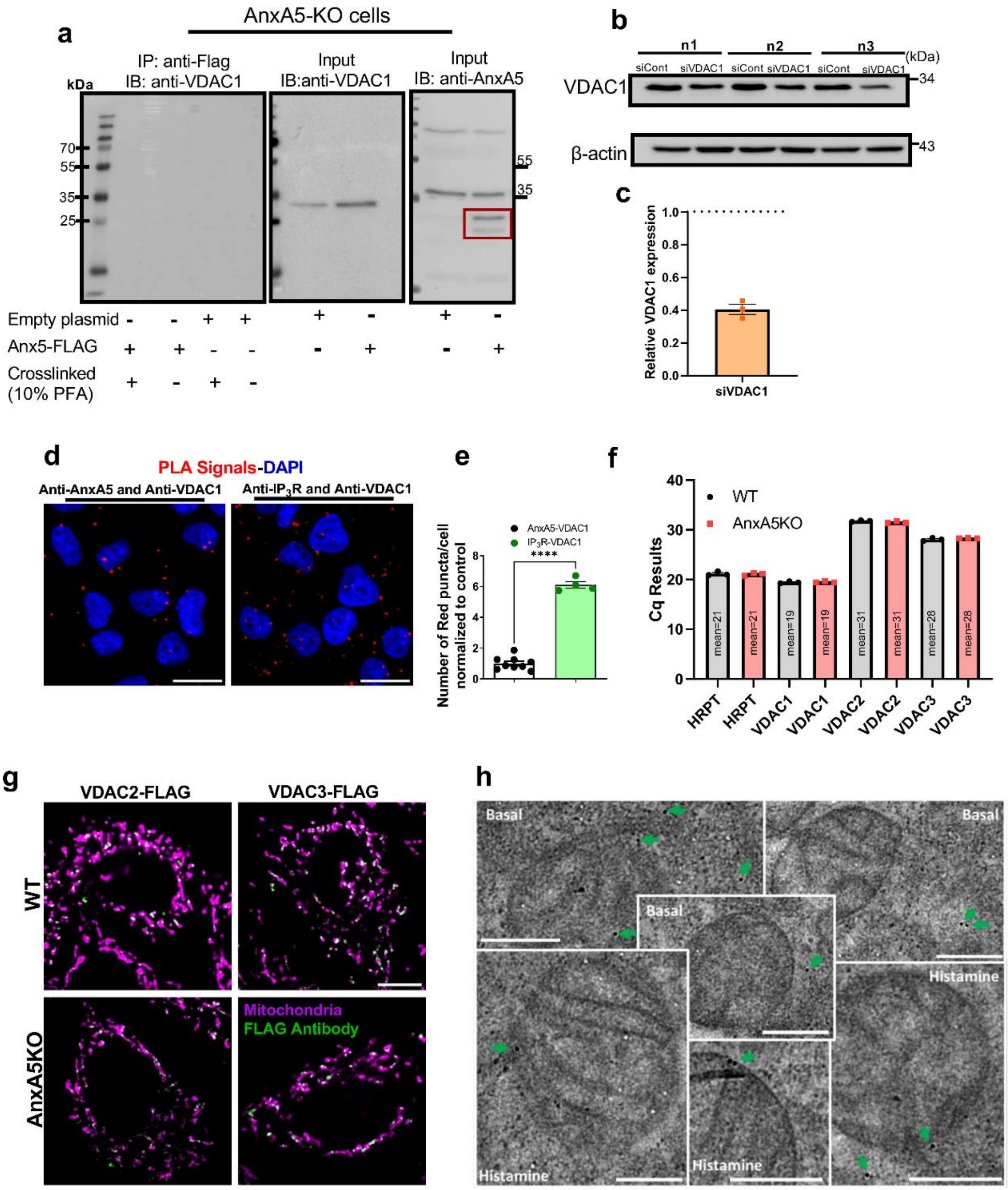

Appendix Figure S2. AnxA5 localizes near VDAC1, and ER Ca2+ release induces its accumulation on the OMM. a Immunoblots show the AnxA5-KO HeLa cells that were transfected either with AnxA5- FLAG or with empty plasmid (set of cells were fixed with 10% PFA as described in methods). AnxA5 was immunoprecipitated from whole cell lysate using the FLAG tag. Co-immunoprecipitation of VDAC1 was checked by immunoblotting with VDAC1-antibody. Uncropped blots are provided in the Source Data. **b** Immunoblots show the expression level of VDAC1 transfected either with siNeg or siVDAC1. **c** Bar graph represents immunoblot analysis of VDAC1 expression as mean ± SEM (nsiControl =3; nsiVDAC1 =3). **d** Representative image of PLA assay indicating the protein-protein proximity between AnxA5-VDAC1 (left panel) and IP3R- VDAC1 (right panel) in HeLa

cells (scale bar = 10 µm). **e** Bar graph shows the ratio of total PLA signals to the number of cells. Data points represent the mean ± SEM (nAnxA5-VDAC1 =2; nIP3R-VDAC1 =2). **f** Bar graph represents the cycle threshold (Cq) values of HRPT1, VDAC1, VDAC2, and VDAC3 genes in WT and AnxA5KO cells, shown as mean ± SEM (nWT = 3; nAnxA5KO = 3). **g** Representative confocal images of WT and AnxA5-KO HeLa cells expressing FLAG-tagged VDAC2 and VDAC3 (green), stained with MTR-CMX (magenta), were captured (Scale bars = 10 µm). **h** Representative immunoelectron micrographs of mitochondria from HeLa cells (Scale bars = 200 nm). Individual gold particles (indicated by green arrows) represent the localization of AnxA5 under basal conditions (upper images) or 20 seconds after histamine stimulation (lower images). This panel shows a redisplay of content from Figure 3B.

Appendix Fig. S3

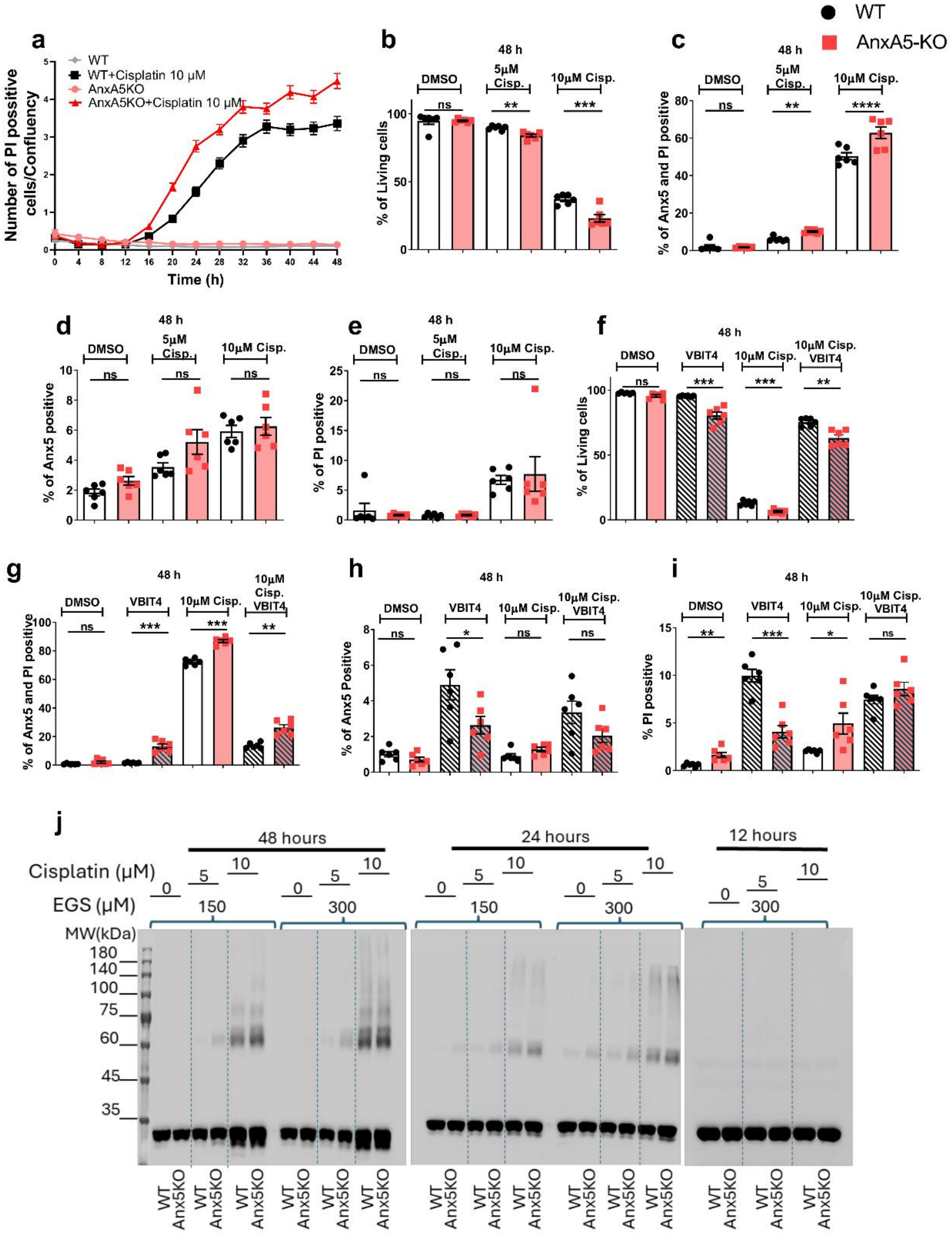

Appendix Figure S3. AnxA5-KO cells are more susceptible to cisplatin-induced apoptosis. a Time course of cisplatin-induced cell death in WT (gray), WT + cisplatin (black), AnxA5-KO (light red), and AnxA5-KO + cisplatin (red) cells. Cells were treated with 10 µM cisplatin, stained with 1 µg/ml PI, and imaged every 4 hours using the CellCyteX microscopy system in an incubator. Data points represent the mean ± SEM. (nWT =2; nAnxA5-KO =2). **b** Bar graphs show the percentage of living cells, **c** late apoptosis, **d** early apoptosis, and **e** necrosis in WT (black) and AnxA5-KO (red) HeLa cells upon 48 h DMSO, 5 µM, and 10 µM cisplatin treatment. Data points represent the mean ± SEM (nWT =6; nAnxA5-KO =6). **f** Bar graphs show the percentage of living cells, **g** late apoptosis, **h** early apoptosis, and **i** necrosis in WT (black) and AnxA5-KO (red) cells upon 48 h DMSO, 20 µM VBIT-4, 10 µM cisplatin, and cisplatin + VBIT-4 treatment. Data points represent the mean ± SEM (nWT =6; nAnxA5-KO =6). **j** Immunoblots showing monomeric and dimeric levels of VDAC1 in WT and AnxA5-KO cells treated with 5 µM and 10 µM cisplatin for 12, 24, and 48 hours. Two different concentrations of the crosslinking reagent EGS (ethylene glycol bis(succinimidyl succinate)), 150 µM and 300 µM, were used to more effectively capture the dimerization levels of VDAC1. Significant differences were assessed using the unpaired Student's t-test or Kolmogorov-Smirnov test (* $p < 0.05$, ** $p < 0.01$, *** $p < 0.001$, **** $p < 0.0001$, and ns: not significant).
