# Supplementary material for: Annexin A5 controls VDAC1-dependent mitochondrial Ca2+ homeostasis and determines cellular susceptibility to apoptosis
Source: EMBO J. 2025 May 9;44(12):3413–47. doi: 10.1038/s44318-025-00454-9 (PMC12170872; doi:10.1038/s44318-025-00454-9)

AnxA5 (35–36 kDa) antibody

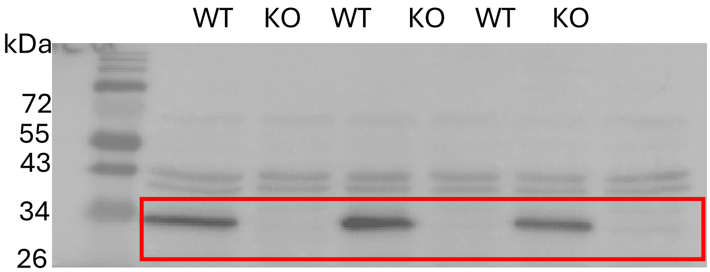

VDAC1 (30–32 kDa) antibody

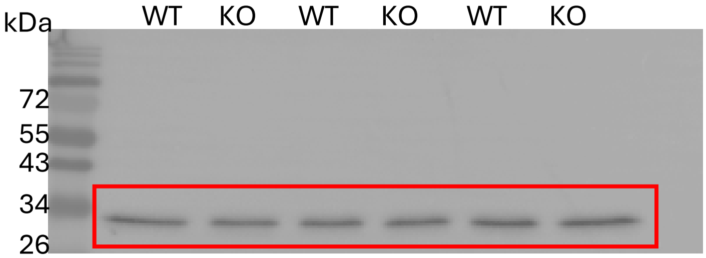

UCP2 (33 kDa) antibody

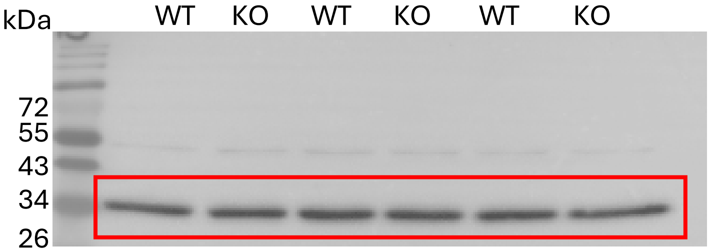

β-Actin antibody (42 kDa) (loading control for VDAC1)

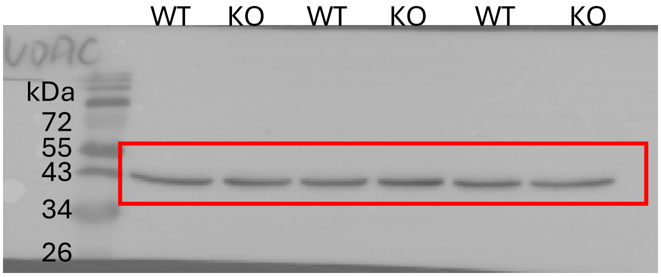

β-Actin antibody (42 kDa) (loading control for UCP2)

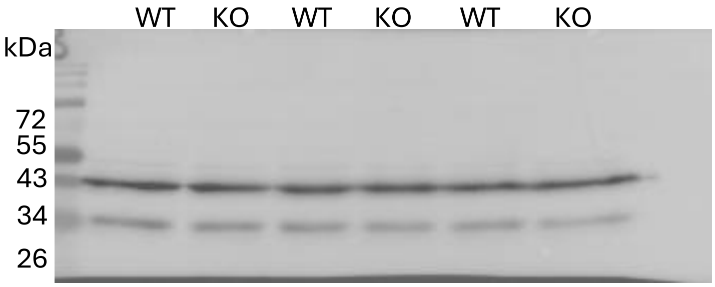

MICU1 (50–54 kDa) antibody

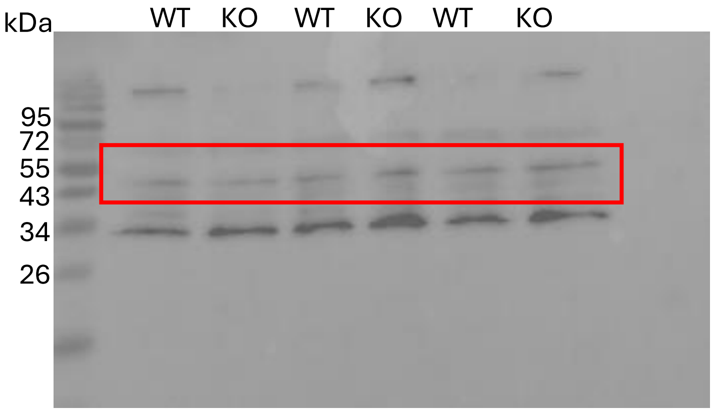

β-Actin antibody (42 kDa) (loading control for MICU1)

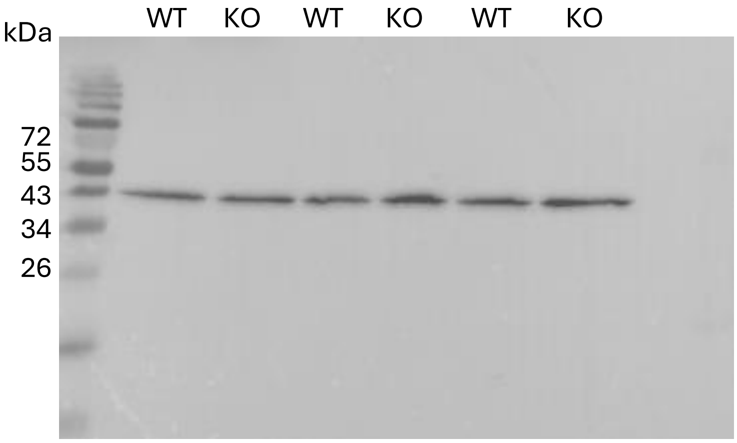

MCU (30 to 35 kDa) antibody

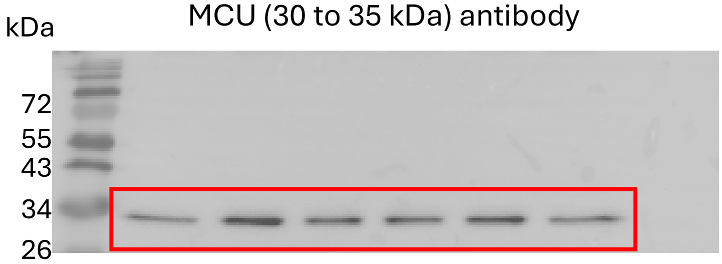

β-Actin antibody (42 kDa) (loading control for MCU blot)

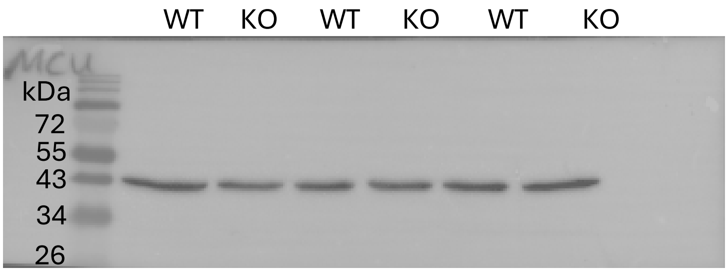

MICU2 (50 kDa) antibody

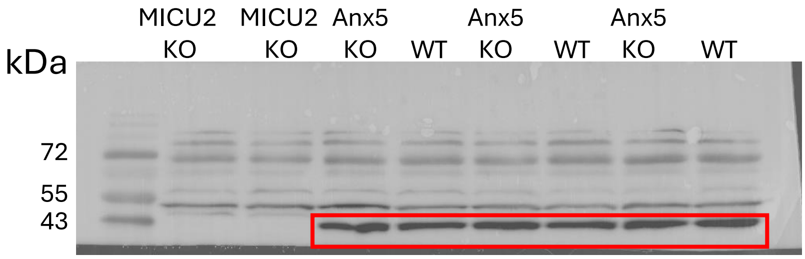

Here, we used MICU2 knockout cells to confirm the correct band of MICU2, as we observed multiple bands. The calculated molecular weight of MICU2 is 50 kDa, but the observed molecular weight is around 43 kDa.

Histone H3 (15 kDa) antibody loading control for MICU2 blot

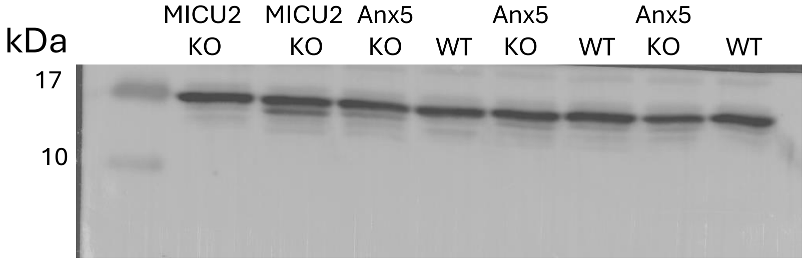

EMRE antibody

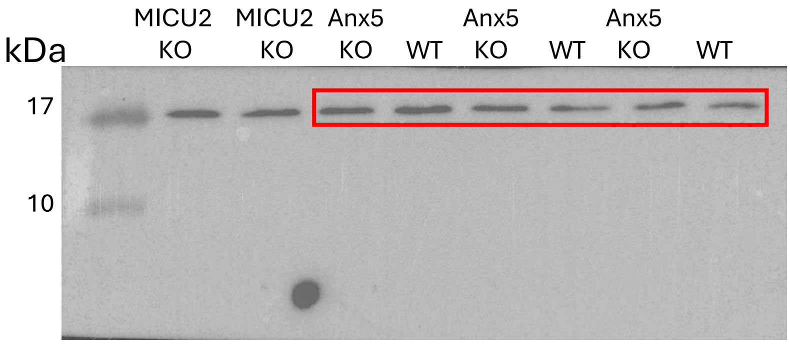

β-Actin (42 kDa) antibody antibody loading control for EMRE blot

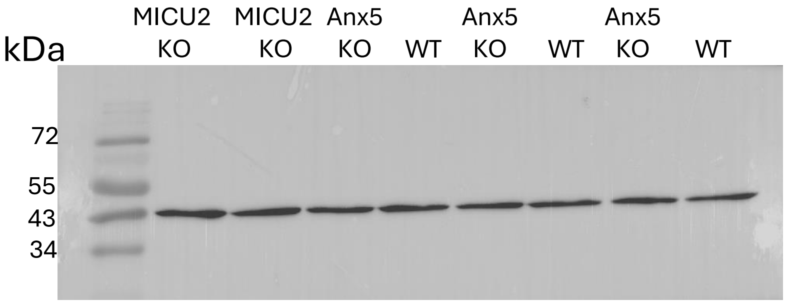

Supplement: Supplementary file 4 — Source data Fig. 2 [file 44318_2025_454_MOESM4_ESM.zip › Figure 2/2C/Figure 2C blots.pdf]
