# Supplementary figures and images for: Annexin A5 controls VDAC1-dependent mitochondrial Ca2+ homeostasis and determines cellular susceptibility to apoptosis
Source: EMBO J. 2025 May 9;44(12):3413–47. doi: 10.1038/s44318-025-00454-9 (PMC12170872; doi:10.1038/s44318-025-00454-9)

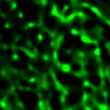

Supplement: Supplementary file 4 — Source data Fig. 2 [file 44318_2025_454_MOESM4_ESM.zip › Figure 2/2E/AnxA5KO-croped-ER.tif]

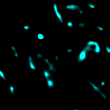

Supplement: Supplementary file 4 — Source data Fig. 2 [file 44318_2025_454_MOESM4_ESM.zip › Figure 2/2E/AnxA5KO-croped-MAMs.tif]

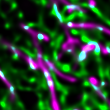

Supplement: Supplementary file 4 — Source data Fig. 2 [file 44318_2025_454_MOESM4_ESM.zip › Figure 2/2E/AnxA5KO-croped-merged.tif]

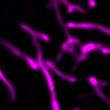

Supplement: Supplementary file 4 — Source data Fig. 2 [file 44318_2025_454_MOESM4_ESM.zip › Figure 2/2E/AnxA5KO-croped-Mito.tif]

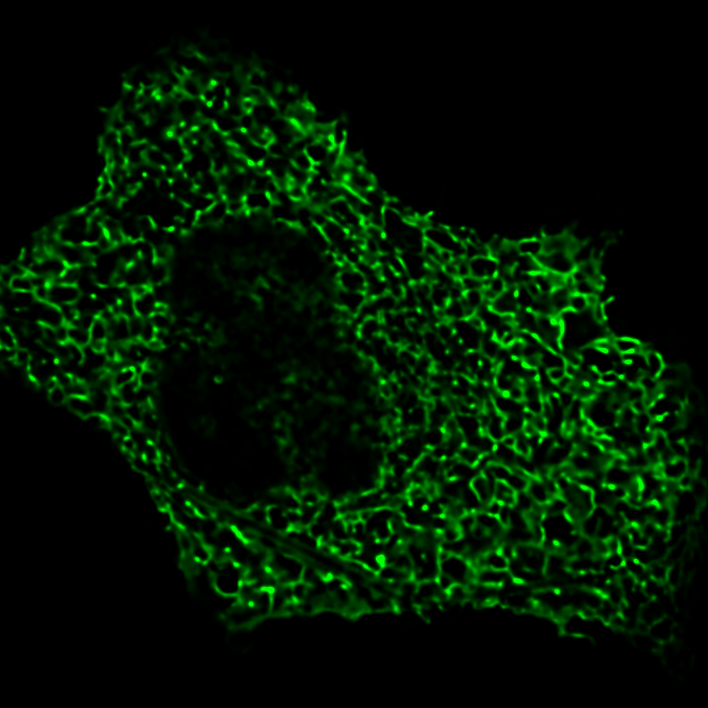

Supplement: Supplementary file 4 — Source data Fig. 2 [file 44318_2025_454_MOESM4_ESM.zip › Figure 2/2E/AnxA5KO-ER.tif]

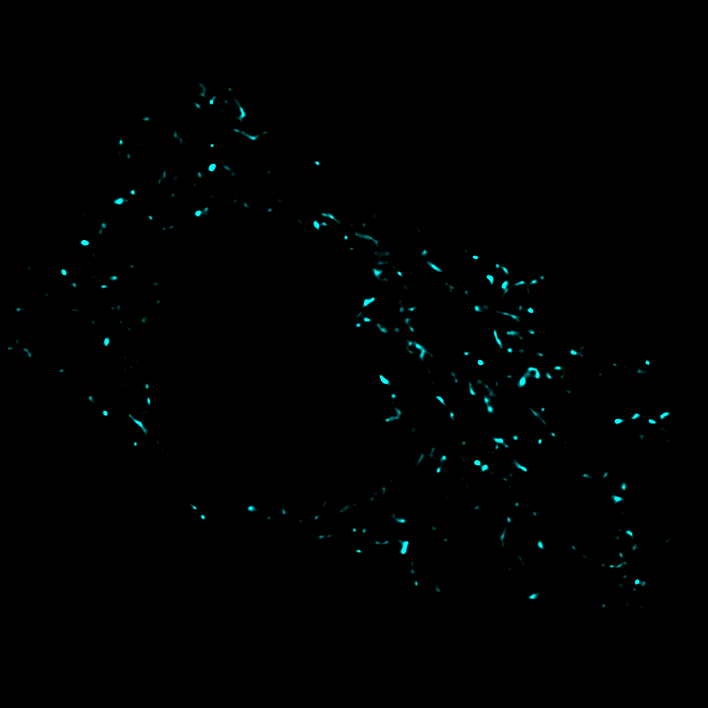

Supplement: Supplementary file 4 — Source data Fig. 2 [file 44318_2025_454_MOESM4_ESM.zip › Figure 2/2E/AnxA5KO-MAMs.tif]

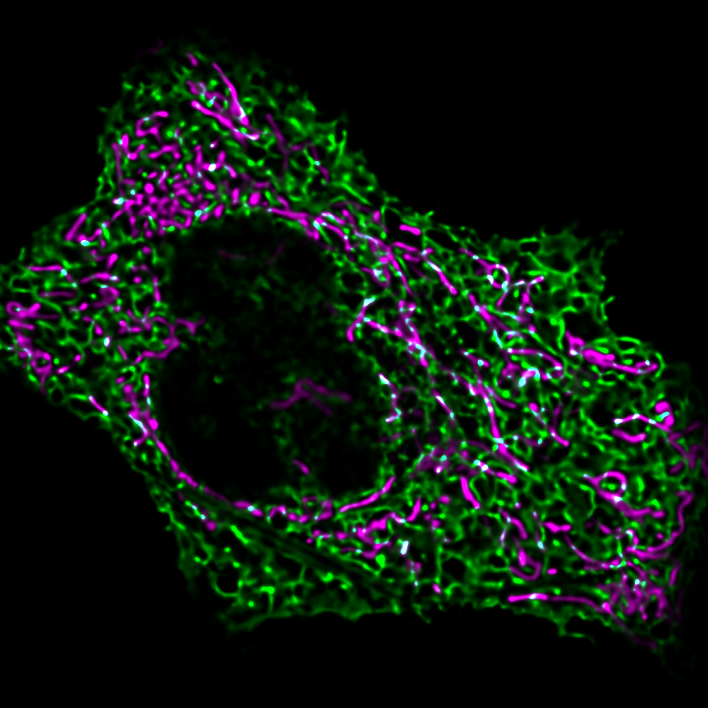

Supplement: Supplementary file 4 — Source data Fig. 2 [file 44318_2025_454_MOESM4_ESM.zip › Figure 2/2E/AnxA5KO-merged.tif]

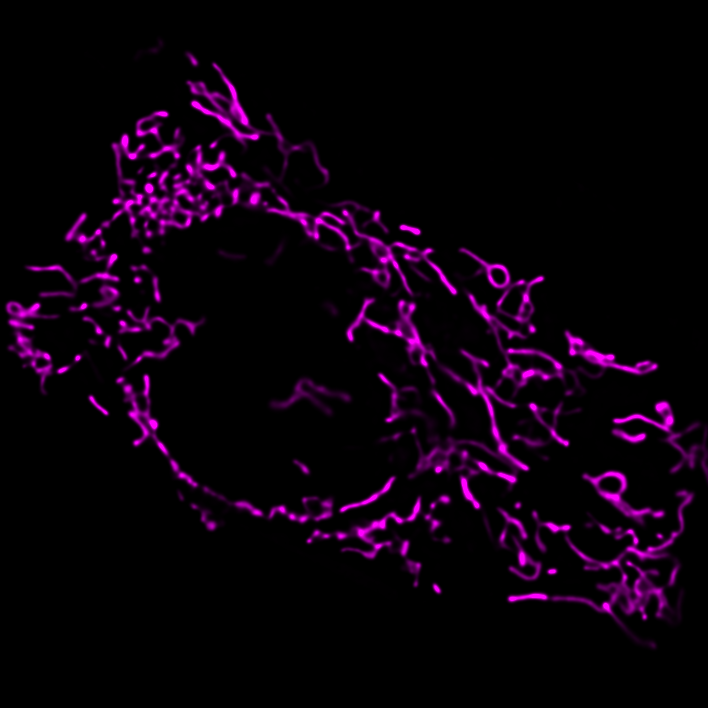

Supplement: Supplementary file 4 — Source data Fig. 2 [file 44318_2025_454_MOESM4_ESM.zip › Figure 2/2E/AnxA5KO-mito.tif]

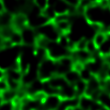

Supplement: Supplementary file 4 — Source data Fig. 2 [file 44318_2025_454_MOESM4_ESM.zip › Figure 2/2E/WT-ER-croped.tif]

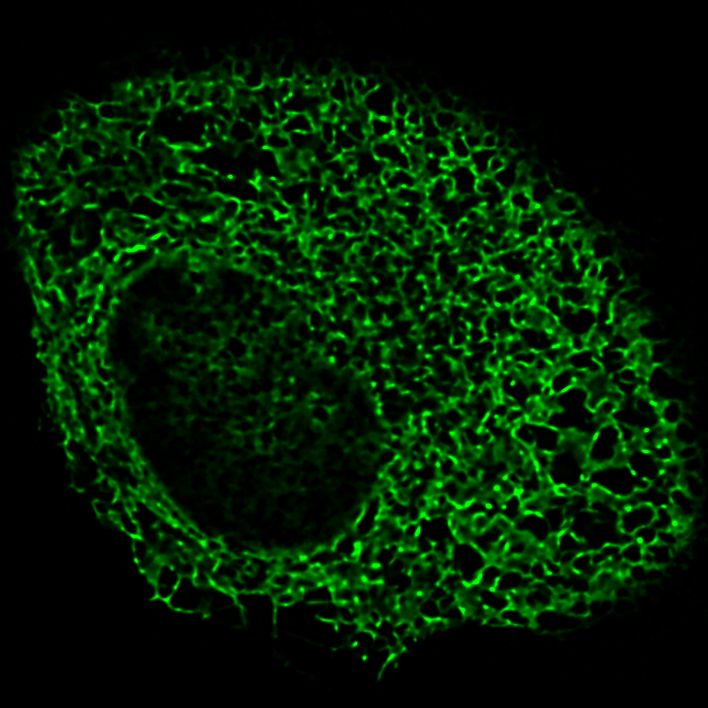

Supplement: Supplementary file 4 — Source data Fig. 2 [file 44318_2025_454_MOESM4_ESM.zip › Figure 2/2E/WT-ER.tif]

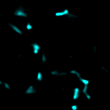

Supplement: Supplementary file 4 — Source data Fig. 2 [file 44318_2025_454_MOESM4_ESM.zip › Figure 2/2E/WT-MAMs-croped.tif]

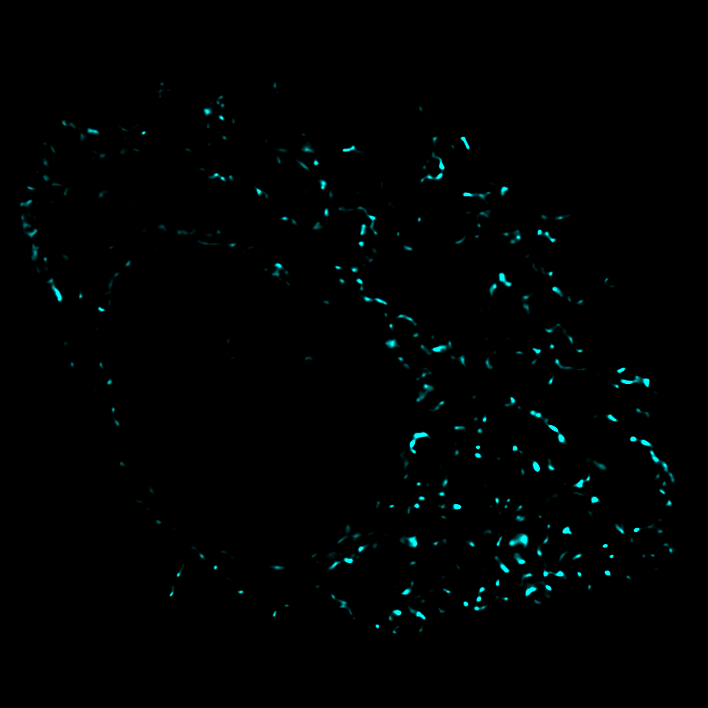

Supplement: Supplementary file 4 — Source data Fig. 2 [file 44318_2025_454_MOESM4_ESM.zip › Figure 2/2E/WT-MAMs.tif]

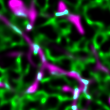

Supplement: Supplementary file 4 — Source data Fig. 2 [file 44318_2025_454_MOESM4_ESM.zip › Figure 2/2E/WT-merged-croped.tif]

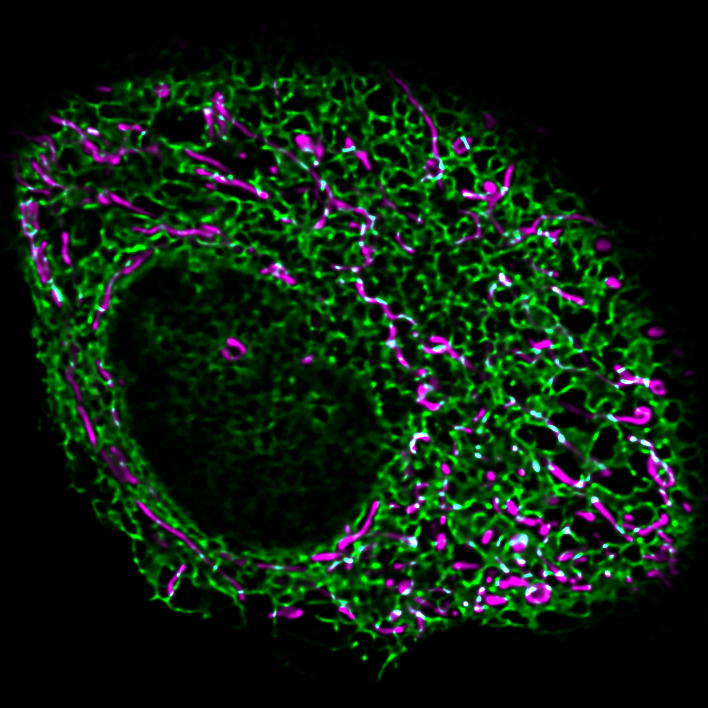

Supplement: Supplementary file 4 — Source data Fig. 2 [file 44318_2025_454_MOESM4_ESM.zip › Figure 2/2E/WT-Merged.tif]

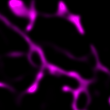

Supplement: Supplementary file 4 — Source data Fig. 2 [file 44318_2025_454_MOESM4_ESM.zip › Figure 2/2E/WT-mito-croped.tif]

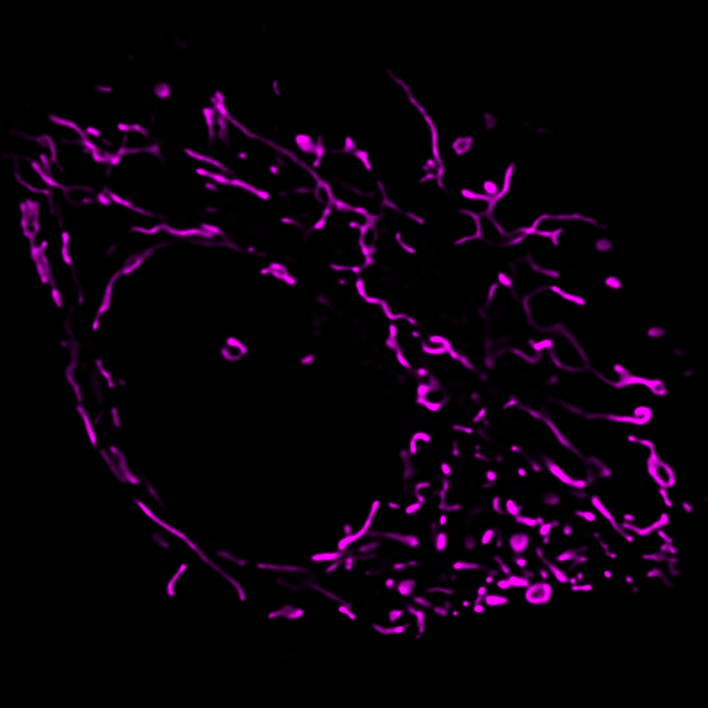

Supplement: Supplementary file 4 — Source data Fig. 2 [file 44318_2025_454_MOESM4_ESM.zip › Figure 2/2E/WT-Mito.tif]

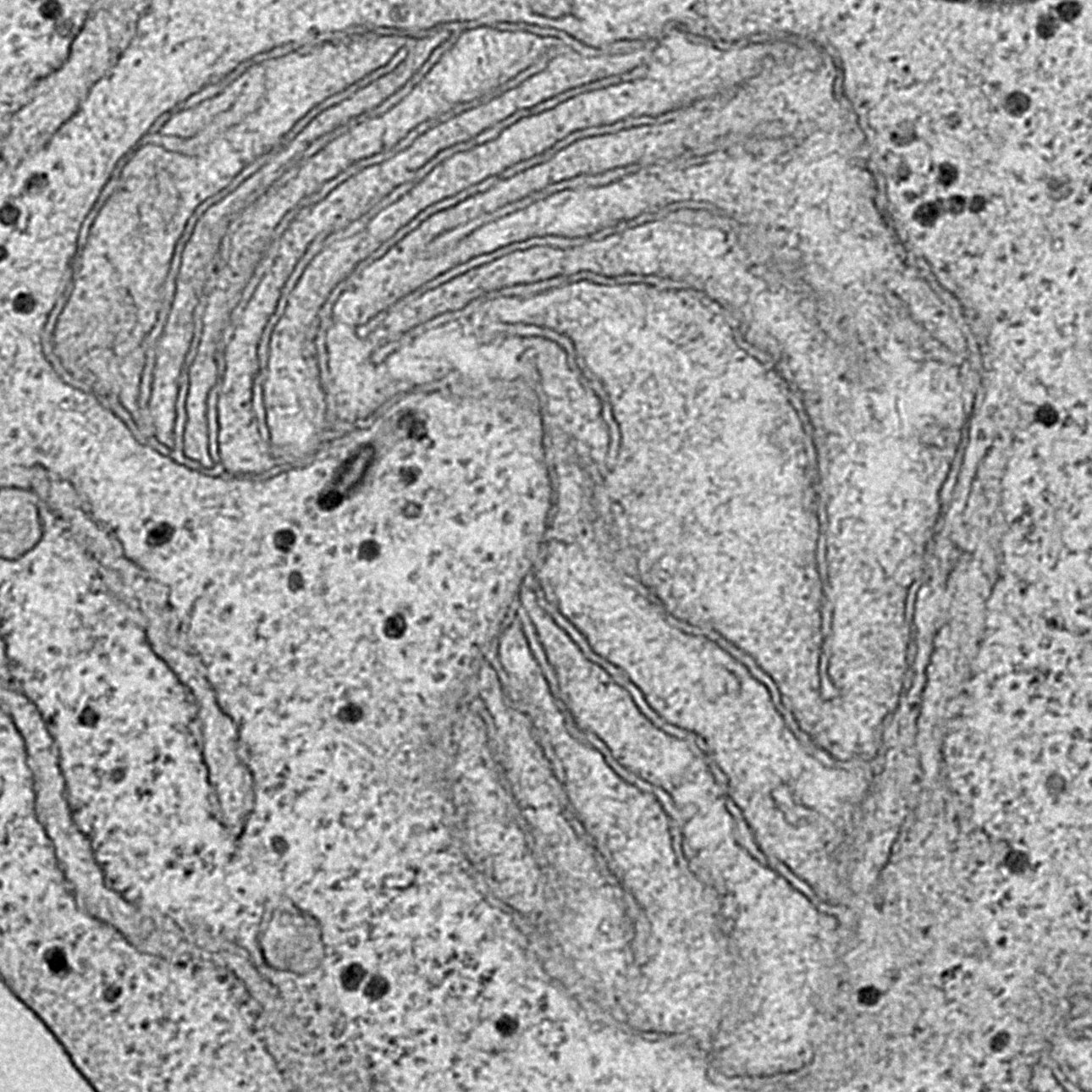

Supplement: Supplementary file 4 — Source data Fig. 2 [file 44318_2025_454_MOESM4_ESM.zip › Figure 2/2I/AnxA5KO-magnification1.tif]

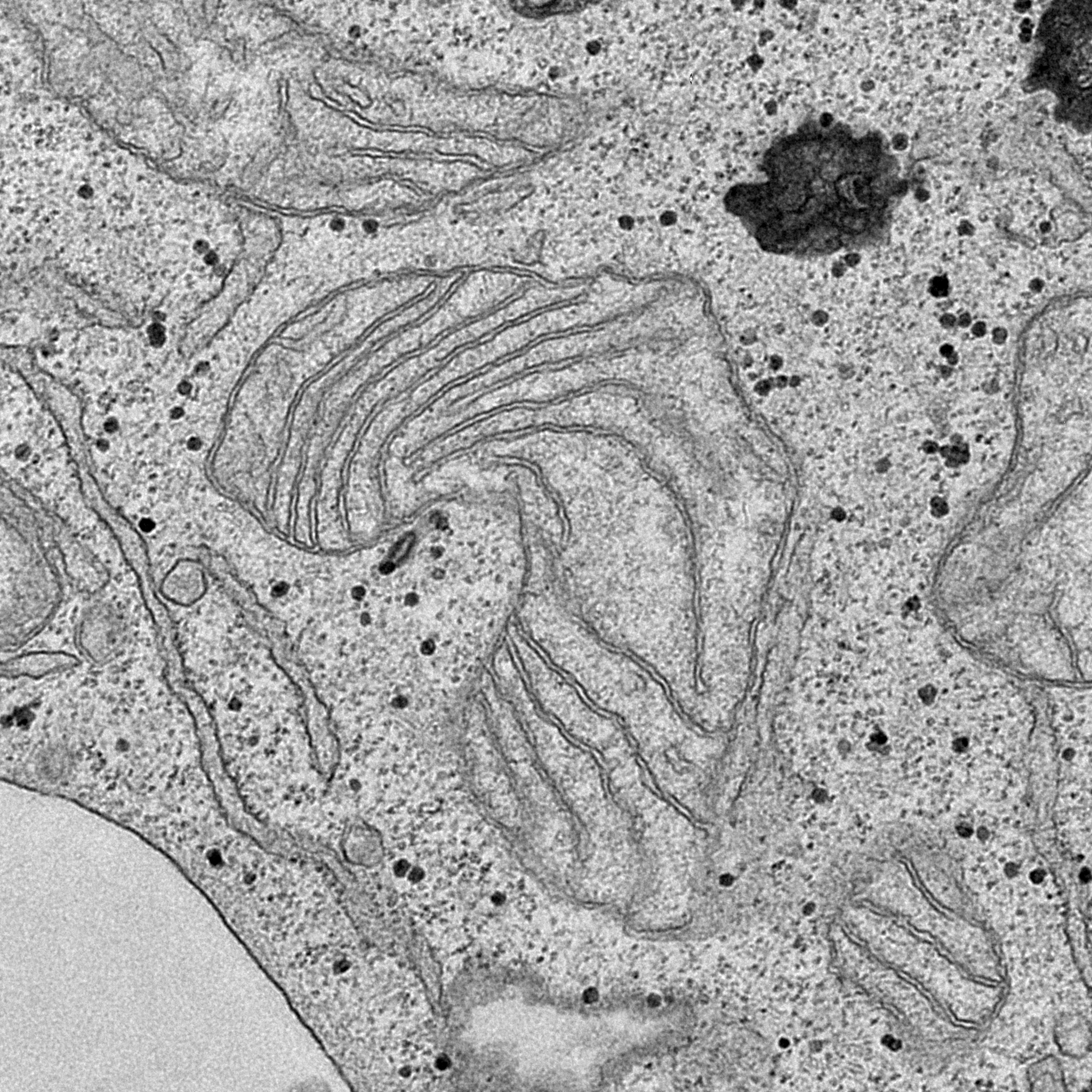

Supplement: Supplementary file 4 — Source data Fig. 2 [file 44318_2025_454_MOESM4_ESM.zip › Figure 2/2I/AnxA5KO-magnification2.tif]

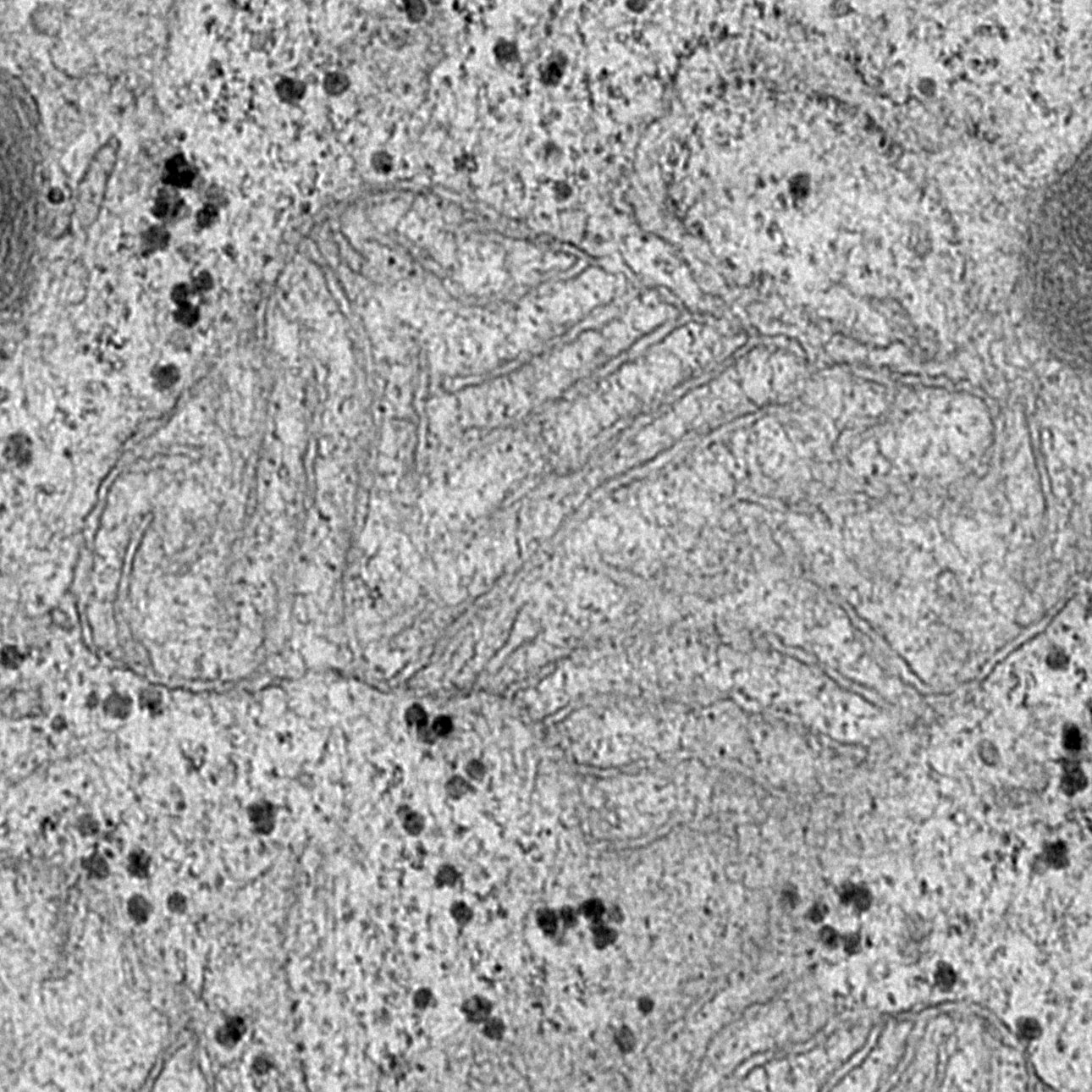

Supplement: Supplementary file 4 — Source data Fig. 2 [file 44318_2025_454_MOESM4_ESM.zip › Figure 2/2I/WT-magnification1.tif]

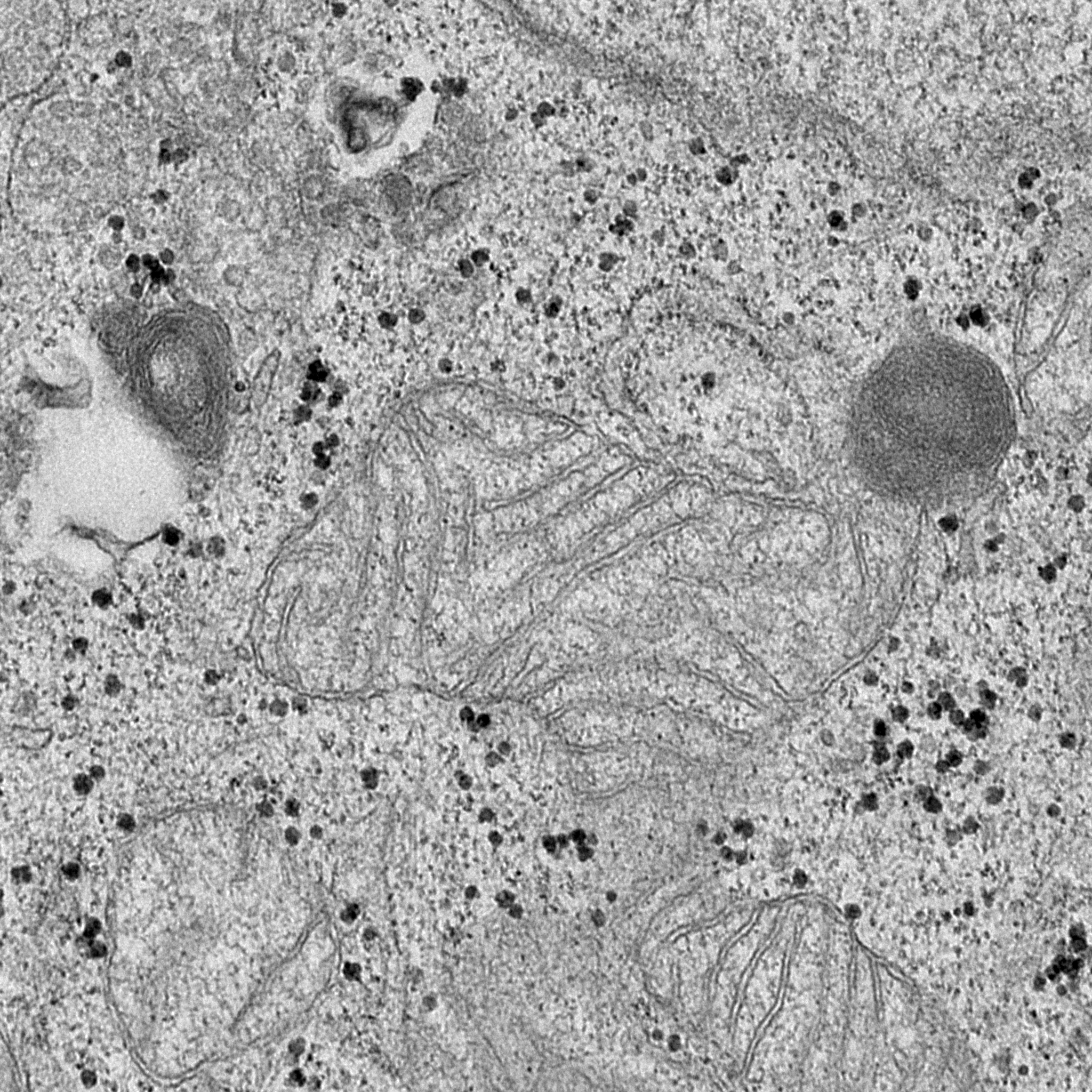

Supplement: Supplementary file 4 — Source data Fig. 2 [file 44318_2025_454_MOESM4_ESM.zip › Figure 2/2I/WT-magnification2.tif]

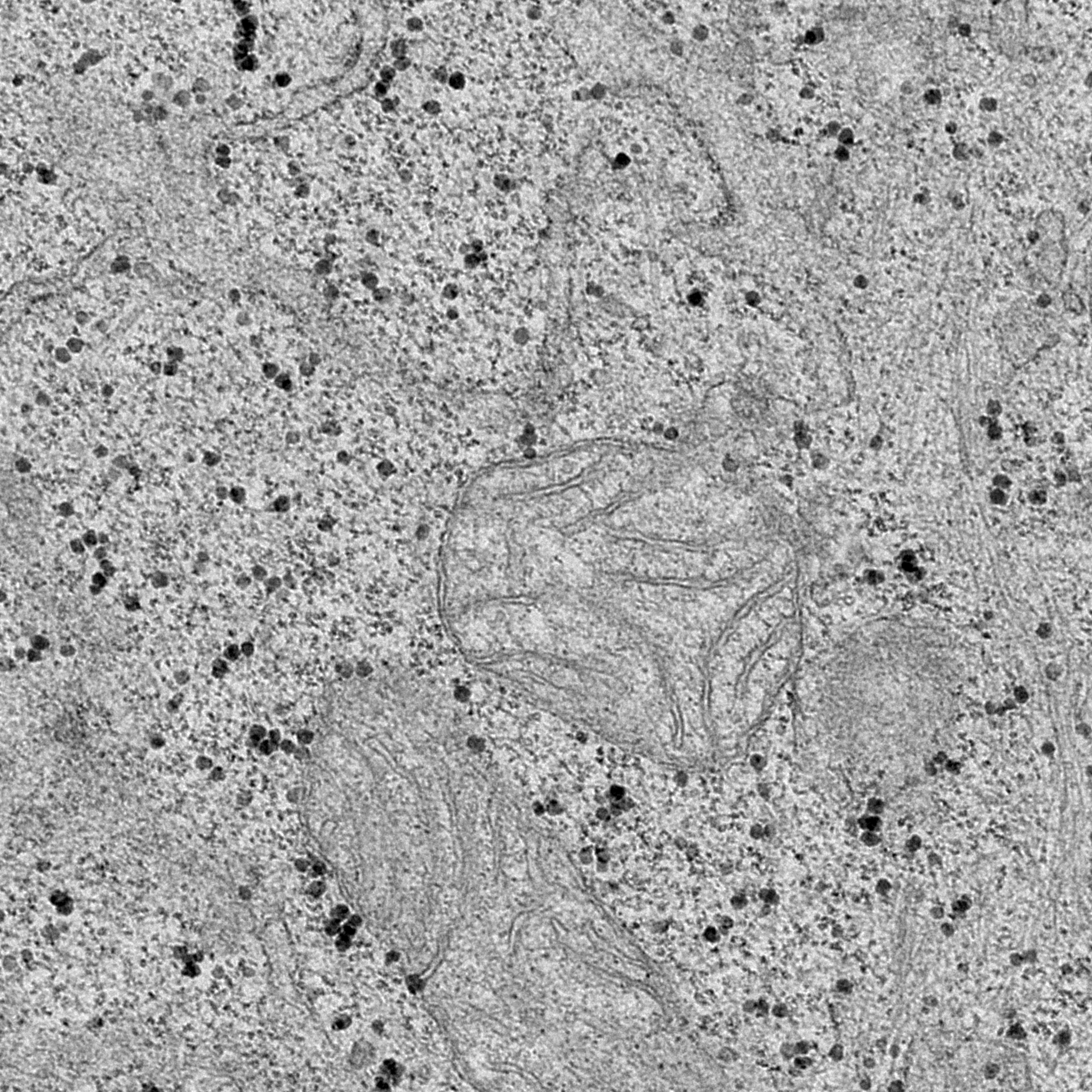

Supplement: Supplementary file 4 — Source data Fig. 2 [file 44318_2025_454_MOESM4_ESM.zip › Figure 2/2L/Figure 2L.tif]

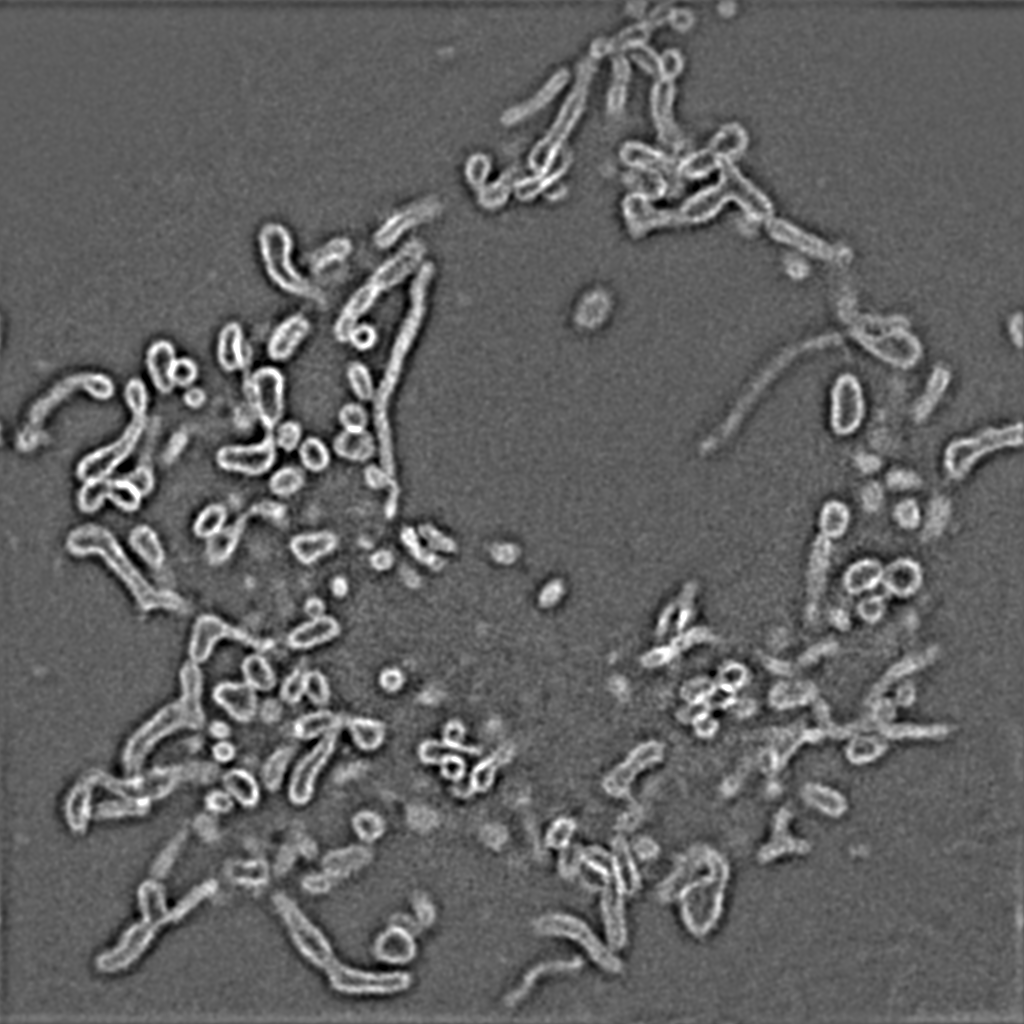

Supplement: Supplementary file 6 — Source data Fig. 4 [file 44318_2025_454_MOESM6_ESM.zip › Figure 4/4H/AnxA5KO.tif]

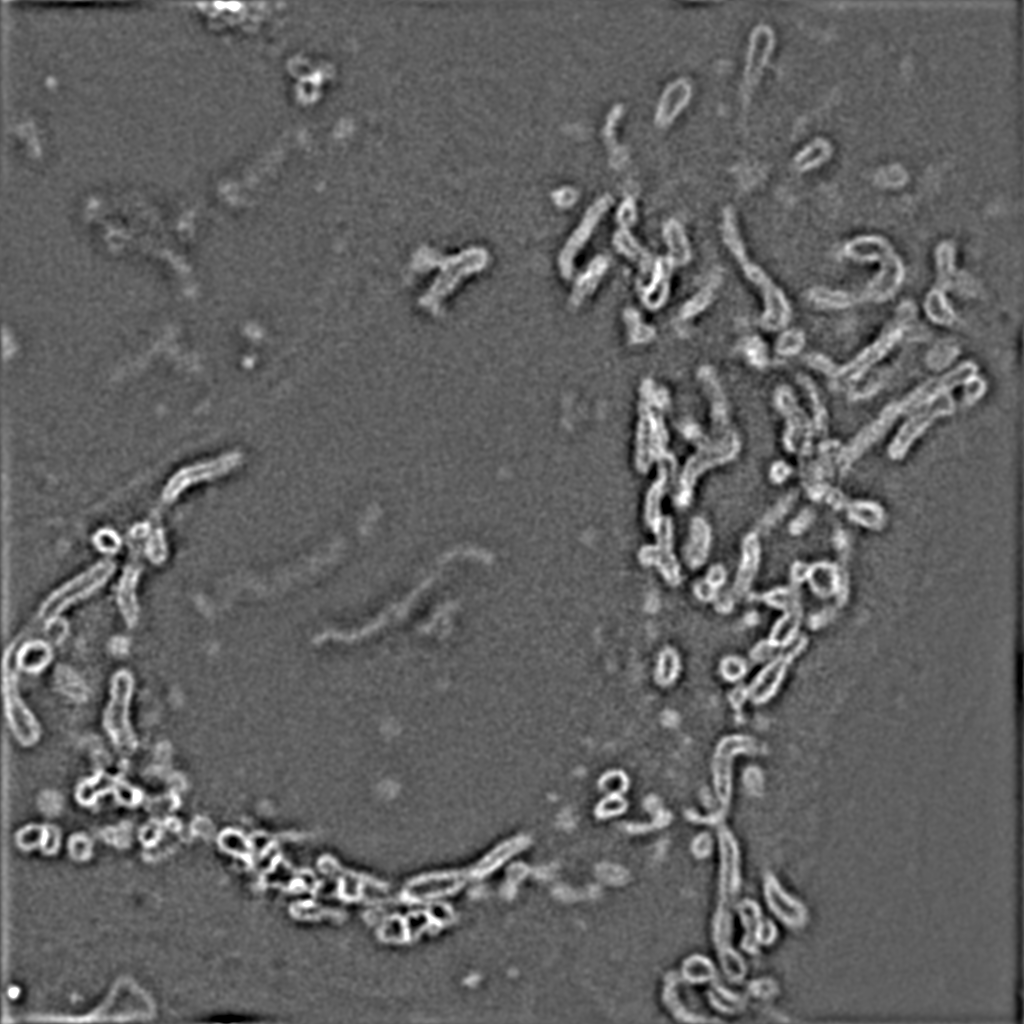

Supplement: Supplementary file 6 — Source data Fig. 4 [file 44318_2025_454_MOESM6_ESM.zip › Figure 4/4H/AnxA5KO_Histamine.tif]

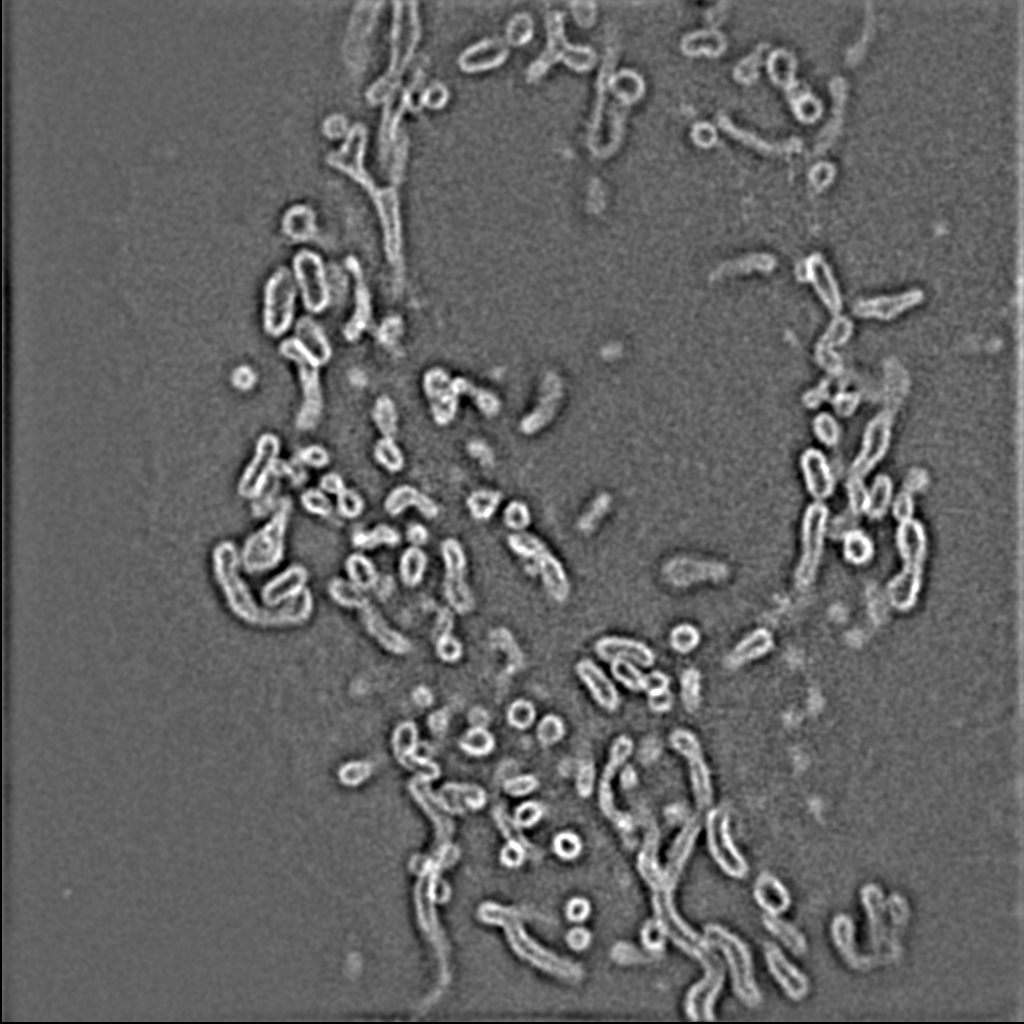

Supplement: Supplementary file 6 — Source data Fig. 4 [file 44318_2025_454_MOESM6_ESM.zip › Figure 4/4H/WT.tif]

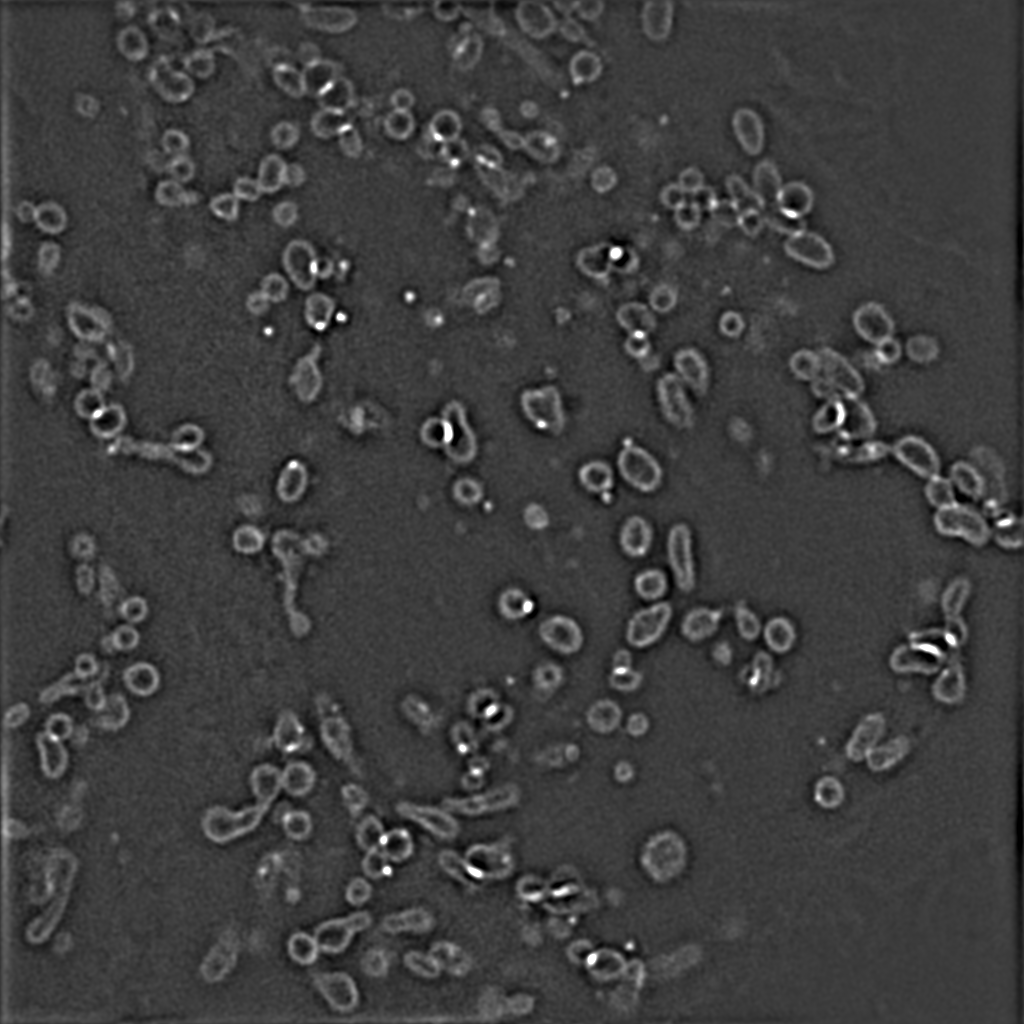

Supplement: Supplementary file 6 — Source data Fig. 4 [file 44318_2025_454_MOESM6_ESM.zip › Figure 4/4H/WT_Histamine.tif]

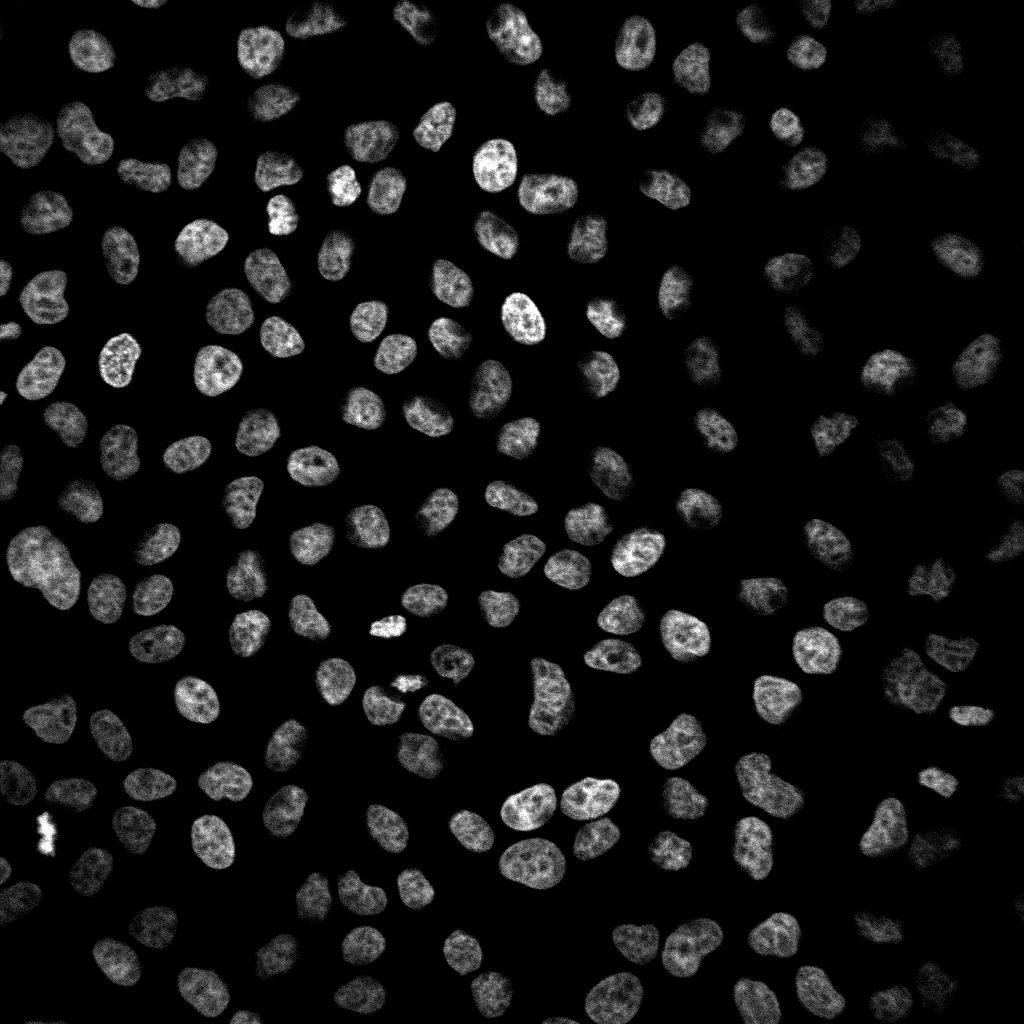

Supplement: Supplementary file 9 — Source data Fig. 5 [file 44318_2025_454_MOESM9_ESM.zip › Figure 5/EMBOJ-2024-119002_SourceDataForFigure5A/-siNeg_AnxA5KO_DAPI.tif]

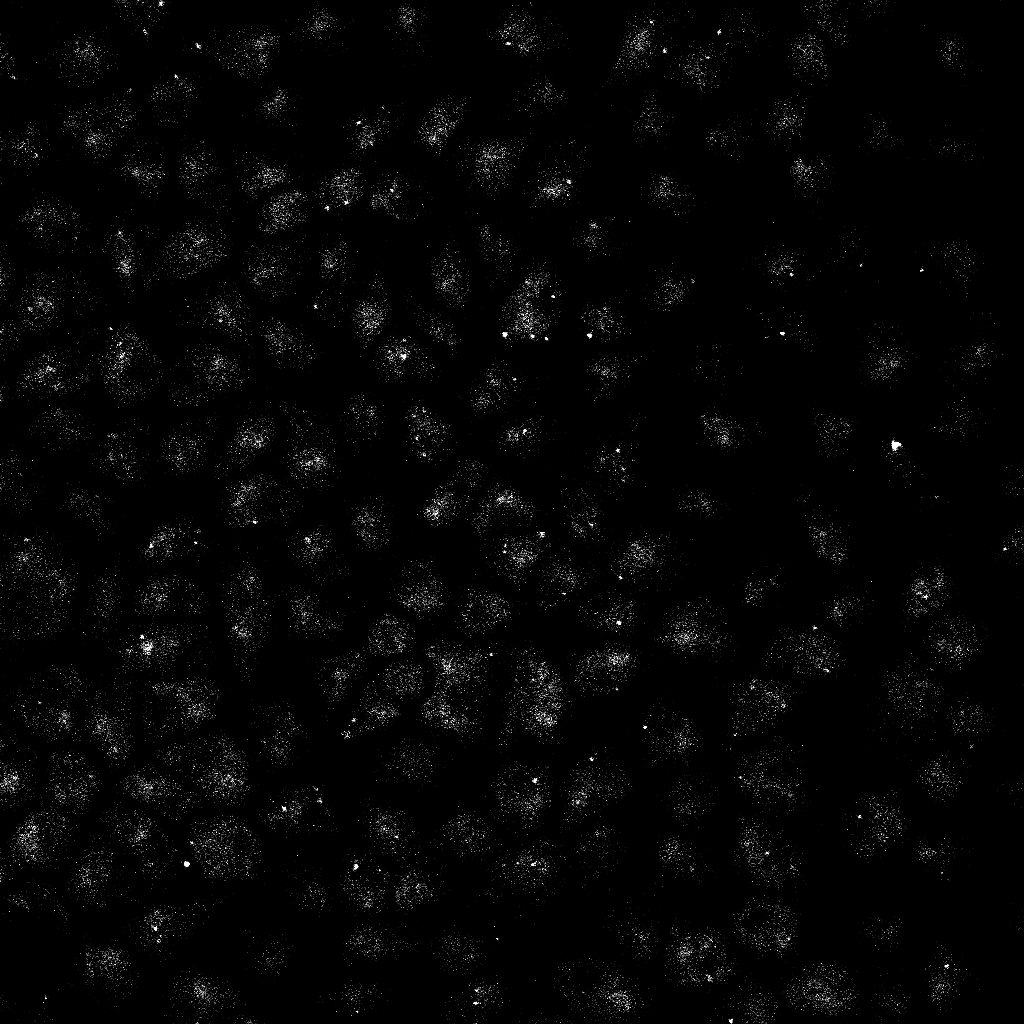

Supplement: Supplementary file 9 — Source data Fig. 5 [file 44318_2025_454_MOESM9_ESM.zip › Figure 5/EMBOJ-2024-119002_SourceDataForFigure5A/-siNeg_AnxA5KO_Merged.tif]

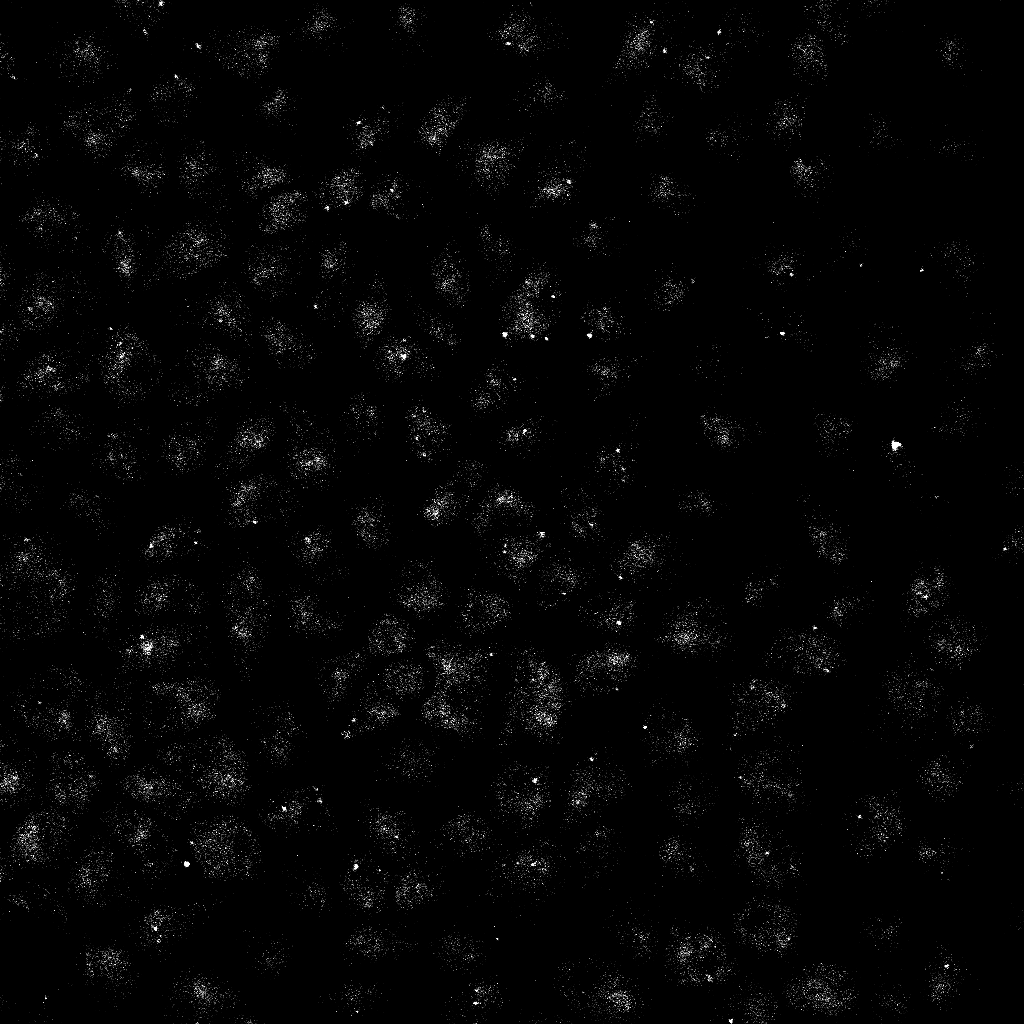

Supplement: Supplementary file 9 — Source data Fig. 5 [file 44318_2025_454_MOESM9_ESM.zip › Figure 5/EMBOJ-2024-119002_SourceDataForFigure5A/-siNeg_AnxA5KO_PLA.tif]

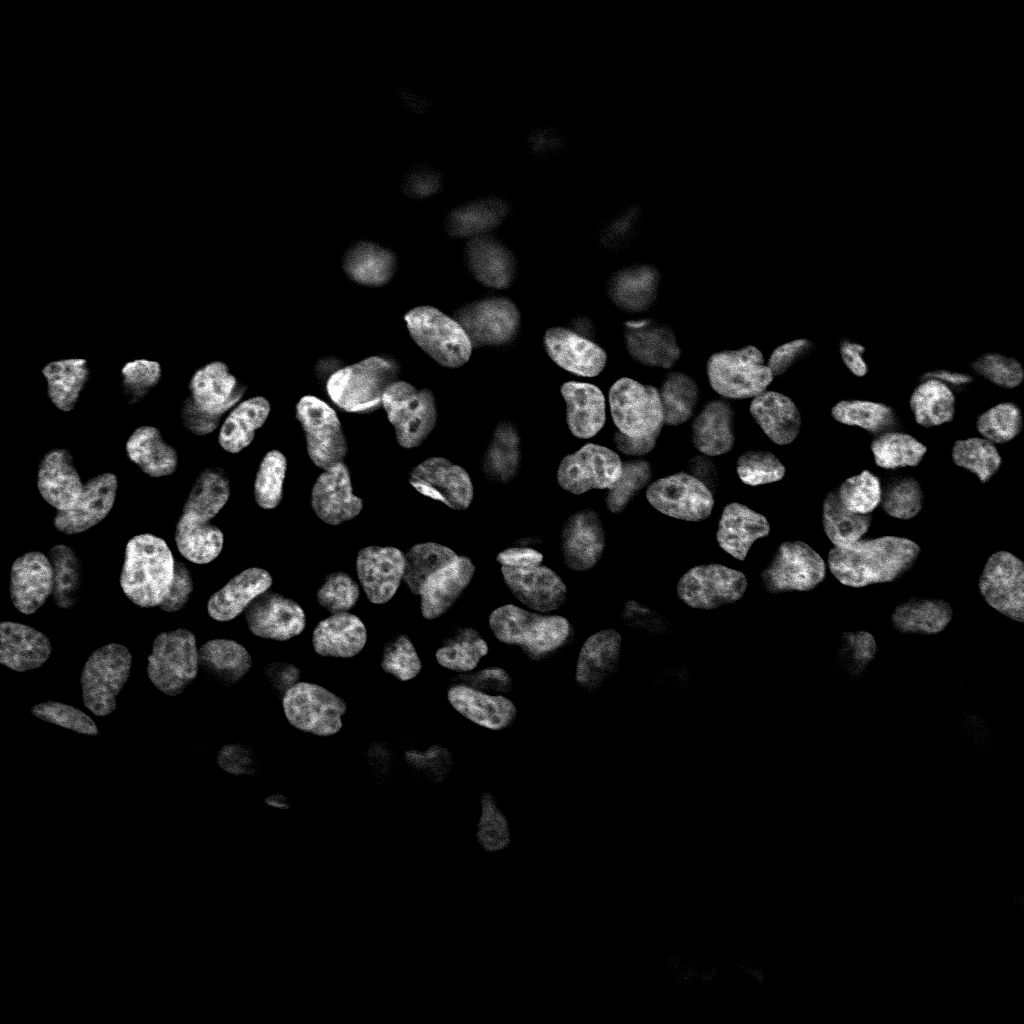

Supplement: Supplementary file 9 — Source data Fig. 5 [file 44318_2025_454_MOESM9_ESM.zip › Figure 5/EMBOJ-2024-119002_SourceDataForFigure5A/siNeg_Wild type_DAPI.tif]

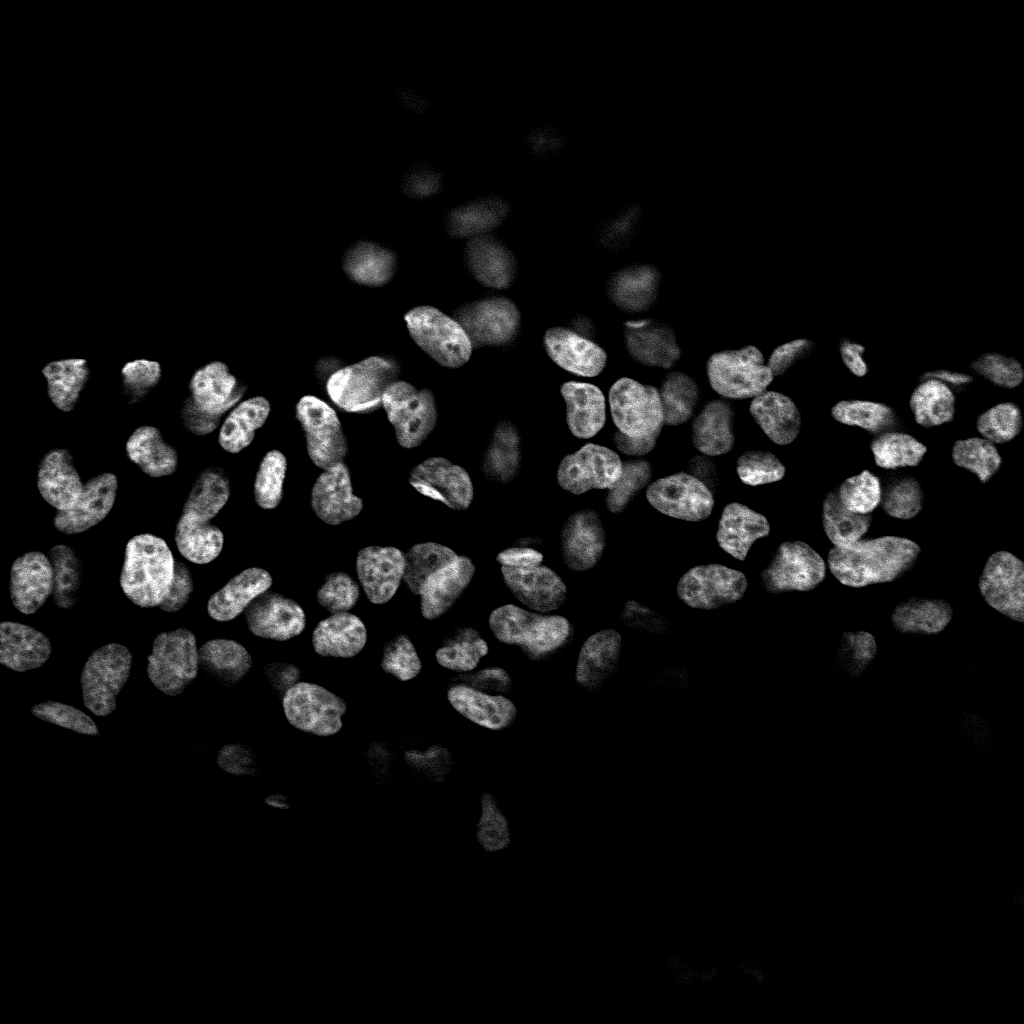

Supplement: Supplementary file 9 — Source data Fig. 5 [file 44318_2025_454_MOESM9_ESM.zip › Figure 5/EMBOJ-2024-119002_SourceDataForFigure5A/siNeg_Wild type_Merged.tif]

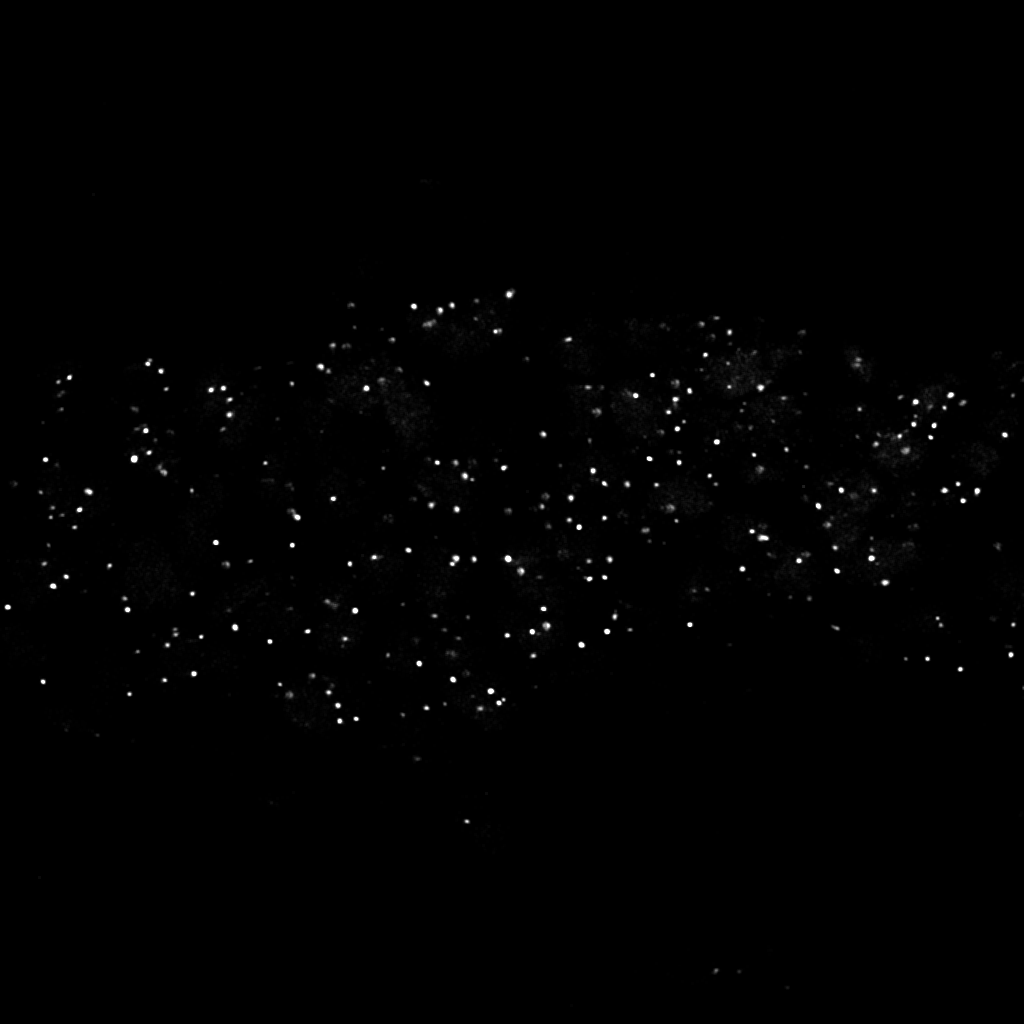

Supplement: Supplementary file 9 — Source data Fig. 5 [file 44318_2025_454_MOESM9_ESM.zip › Figure 5/EMBOJ-2024-119002_SourceDataForFigure5A/siNeg_Wild type_PLA.tif]

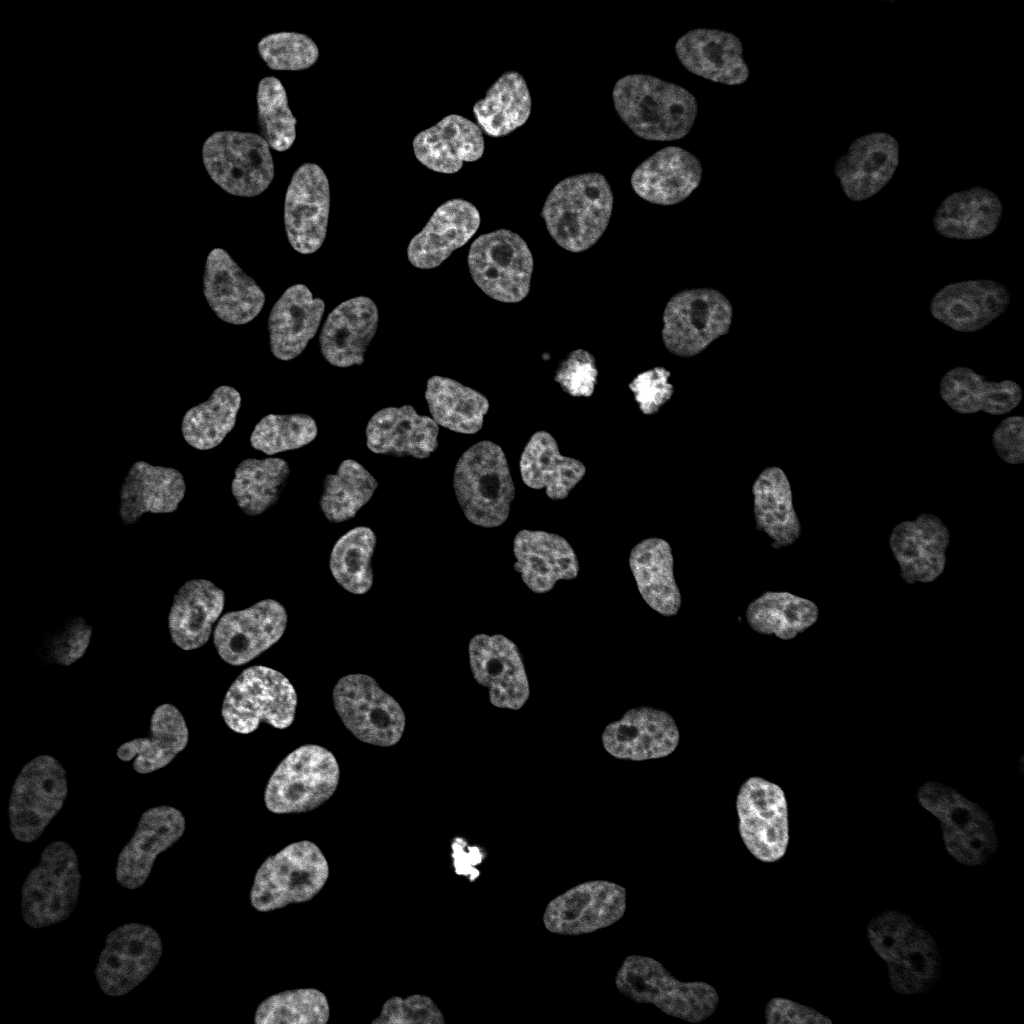

Supplement: Supplementary file 9 — Source data Fig. 5 [file 44318_2025_454_MOESM9_ESM.zip › Figure 5/EMBOJ-2024-119002_SourceDataForFigure5A/siVDAC1_Merged.tif]

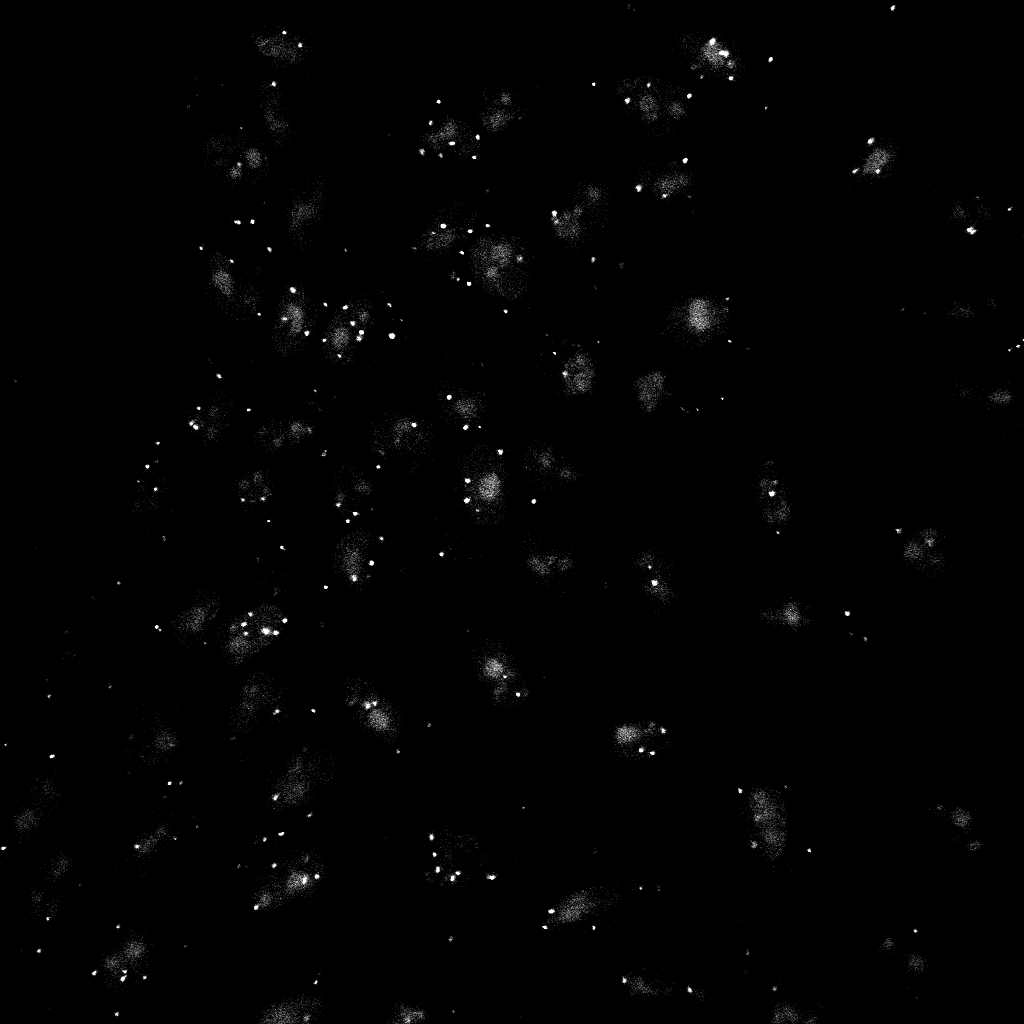

Supplement: Supplementary file 9 — Source data Fig. 5 [file 44318_2025_454_MOESM9_ESM.zip › Figure 5/EMBOJ-2024-119002_SourceDataForFigure5A/siVDAC1_PLA.tif]

shControl

shAnxA5

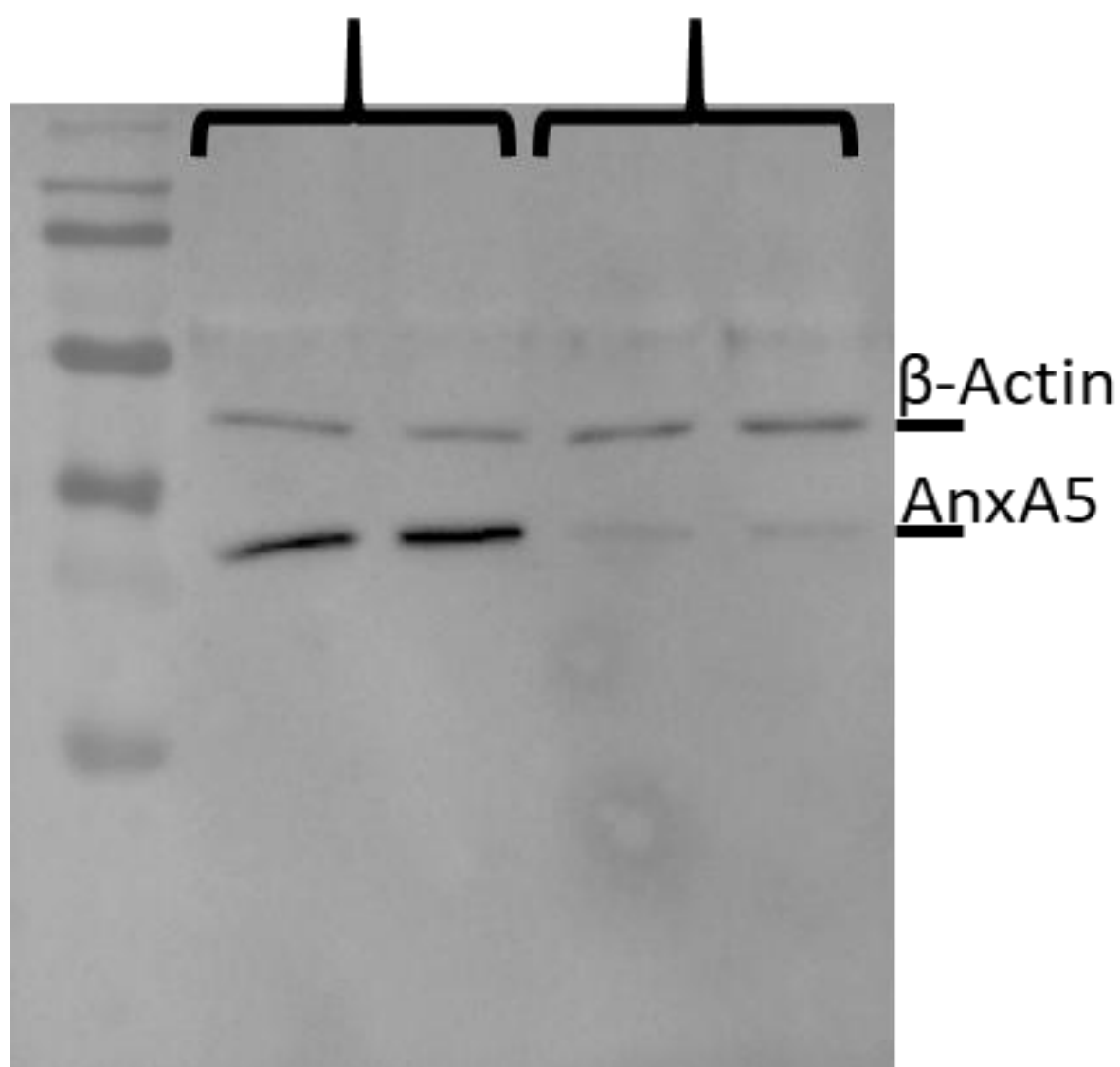

Supplement: Supplementary file 10 — EV and Appendix Figure Source Data [file 44318_2025_454_MOESM10_ESM.zip › EMBOJ-2024-119002R1-EV_and_Appendix_Figures_Source_Data-sd/Fig. EVs source file.zip/Fig EV1/EV1 K/Fig EV1K.pdf]

**AnxA5 (35 kDa) Antibody**

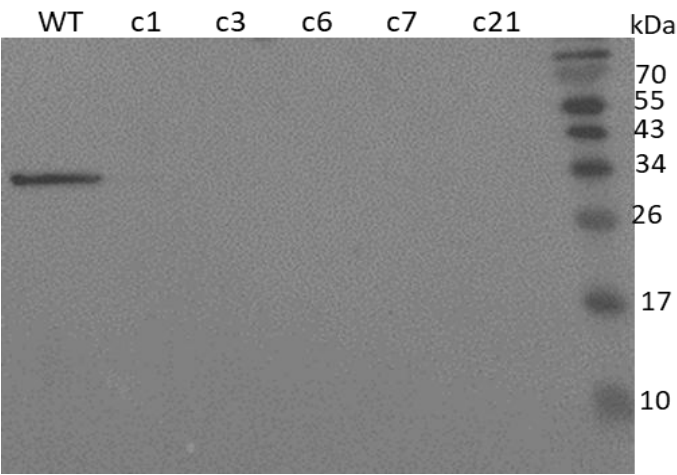

**Histone-H3 (15 kDa) Antibody**

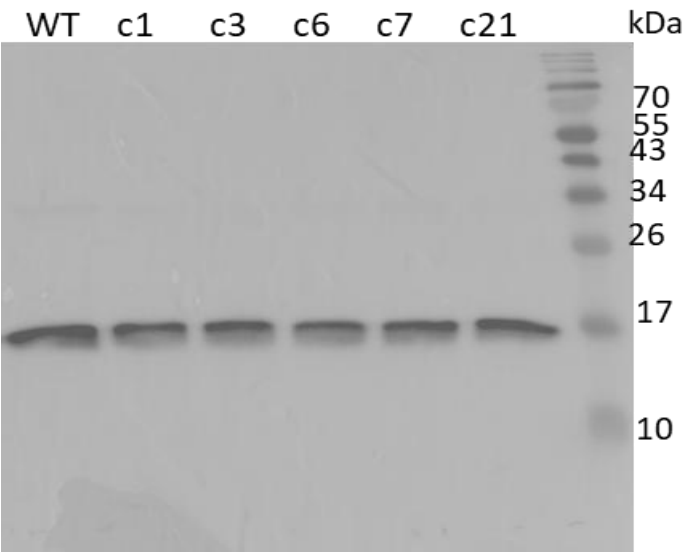

Supplement: Supplementary file 10 — EV and Appendix Figure Source Data [file 44318_2025_454_MOESM10_ESM.zip › EMBOJ-2024-119002R1-EV_and_Appendix_Figures_Source_Data-sd/Fig. EVs source file.zip/Fig EV1/EV1 B/Fig EV1B.pdf]

**Annexin A5 antibody**

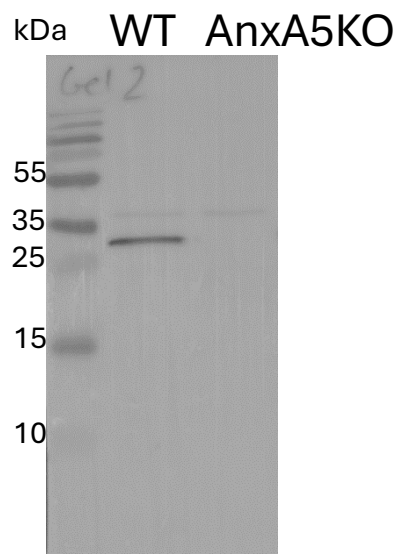

**Histone antibody**

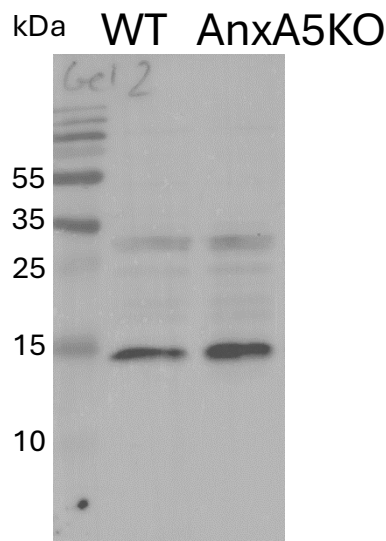

Supplement: Supplementary file 10 — EV and Appendix Figure Source Data [file 44318_2025_454_MOESM10_ESM.zip › EMBOJ-2024-119002R1-EV_and_Appendix_Figures_Source_Data-sd/Fig. EVs source file.zip/Fig EV1/EV1 A/expanded view figure 1a.pdf]

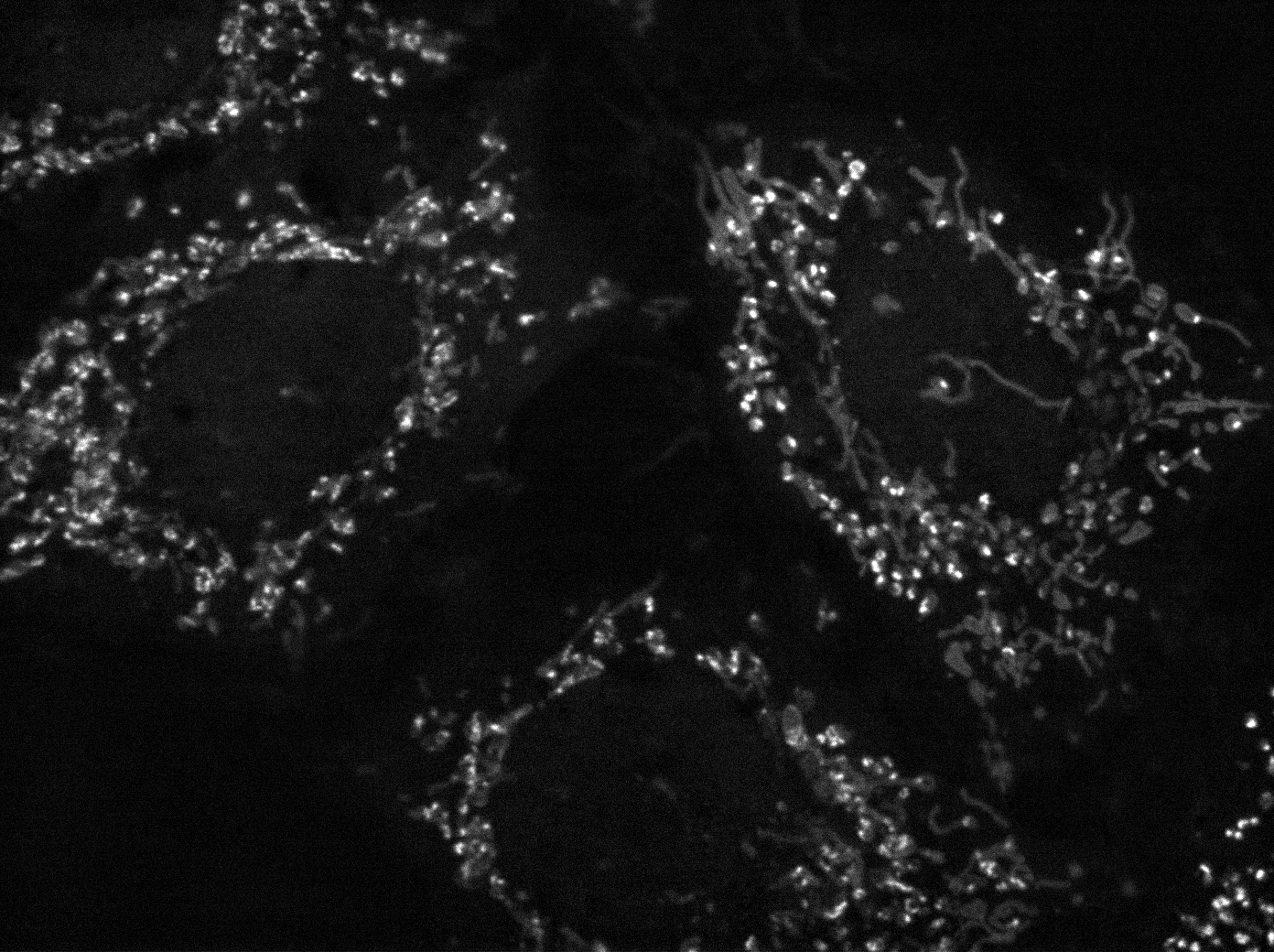

Supplement: Supplementary file 10 — EV and Appendix Figure Source Data [file 44318_2025_454_MOESM10_ESM.zip › EMBOJ-2024-119002R1-EV_and_Appendix_Figures_Source_Data-sd/Fig. EVs source file.zip/Fig EV5/EV5 F/AnxA5KO_DMSO.tif]

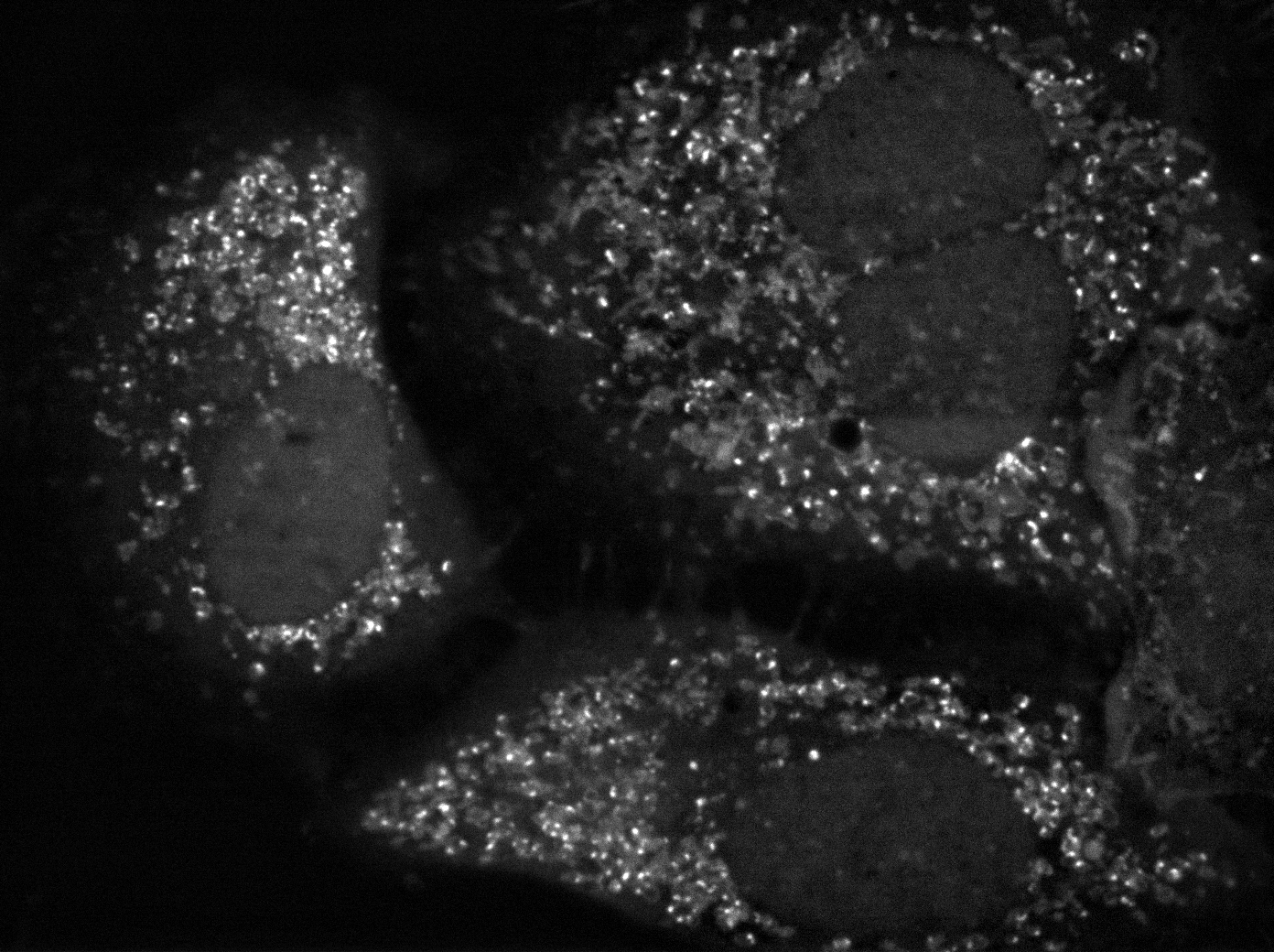

Supplement: Supplementary file 10 — EV and Appendix Figure Source Data [file 44318_2025_454_MOESM10_ESM.zip › EMBOJ-2024-119002R1-EV_and_Appendix_Figures_Source_Data-sd/Fig. EVs source file.zip/Fig EV5/EV5 F/AnxA5KO_VBIT4.tif]

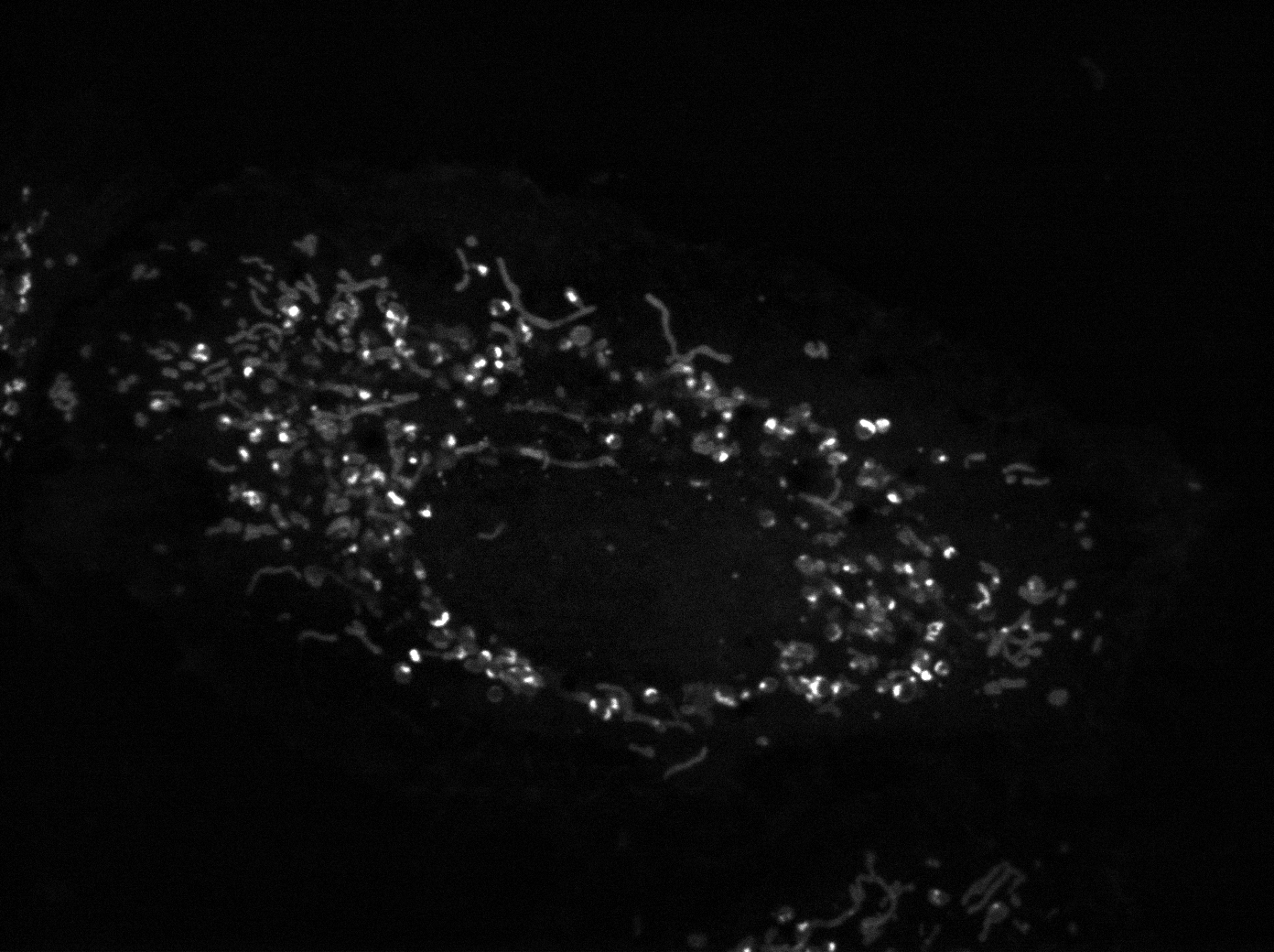

Supplement: Supplementary file 10 — EV and Appendix Figure Source Data [file 44318_2025_454_MOESM10_ESM.zip › EMBOJ-2024-119002R1-EV_and_Appendix_Figures_Source_Data-sd/Fig. EVs source file.zip/Fig EV5/EV5 F/WT_DMSO.tif]

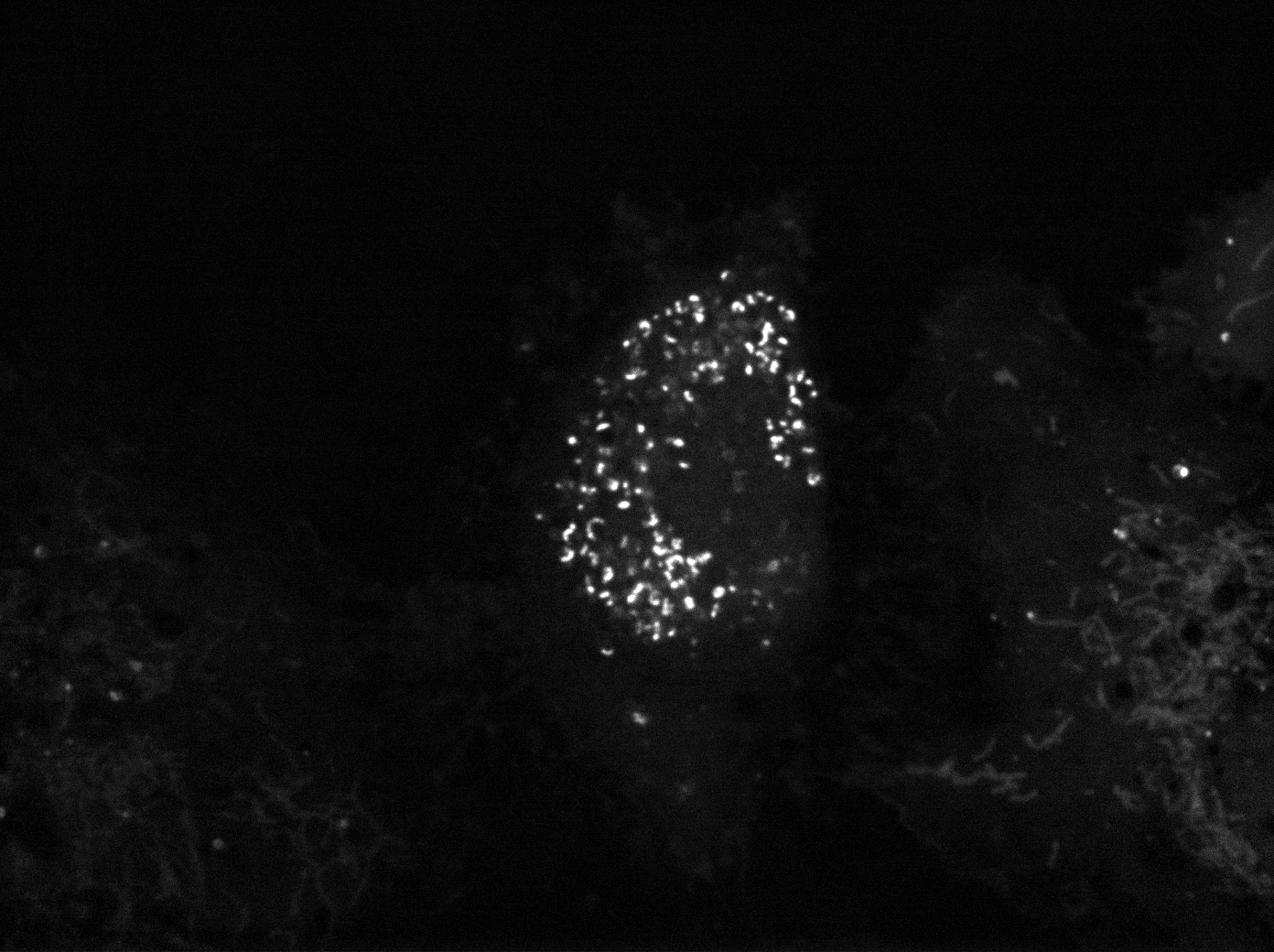

Supplement: Supplementary file 10 — EV and Appendix Figure Source Data [file 44318_2025_454_MOESM10_ESM.zip › EMBOJ-2024-119002R1-EV_and_Appendix_Figures_Source_Data-sd/Fig. EVs source file.zip/Fig EV5/EV5 F/WT_Cisplatin.tif]

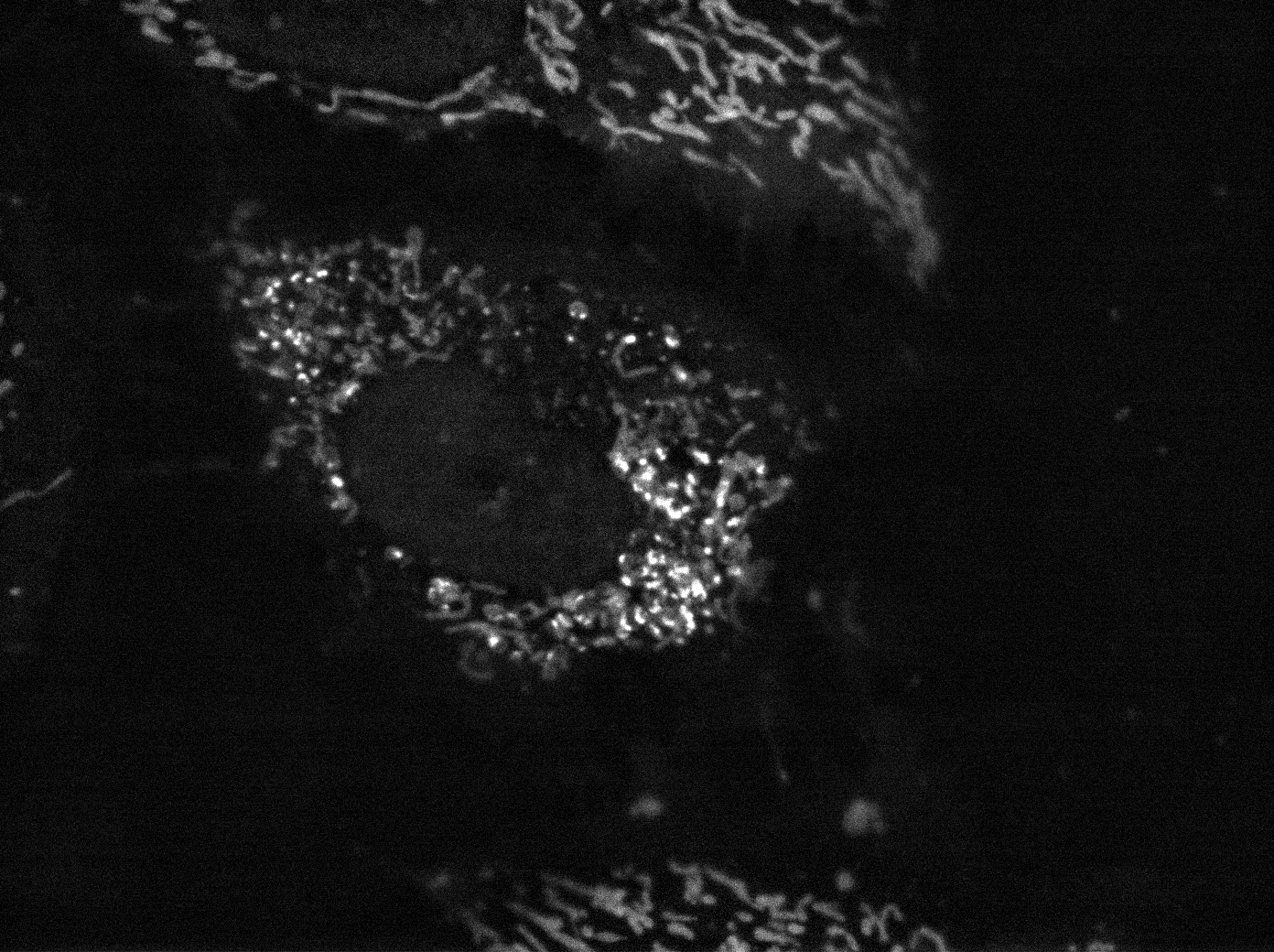

Supplement: Supplementary file 10 — EV and Appendix Figure Source Data [file 44318_2025_454_MOESM10_ESM.zip › EMBOJ-2024-119002R1-EV_and_Appendix_Figures_Source_Data-sd/Fig. EVs source file.zip/Fig EV5/EV5 F/WT_VBIT4.tif]

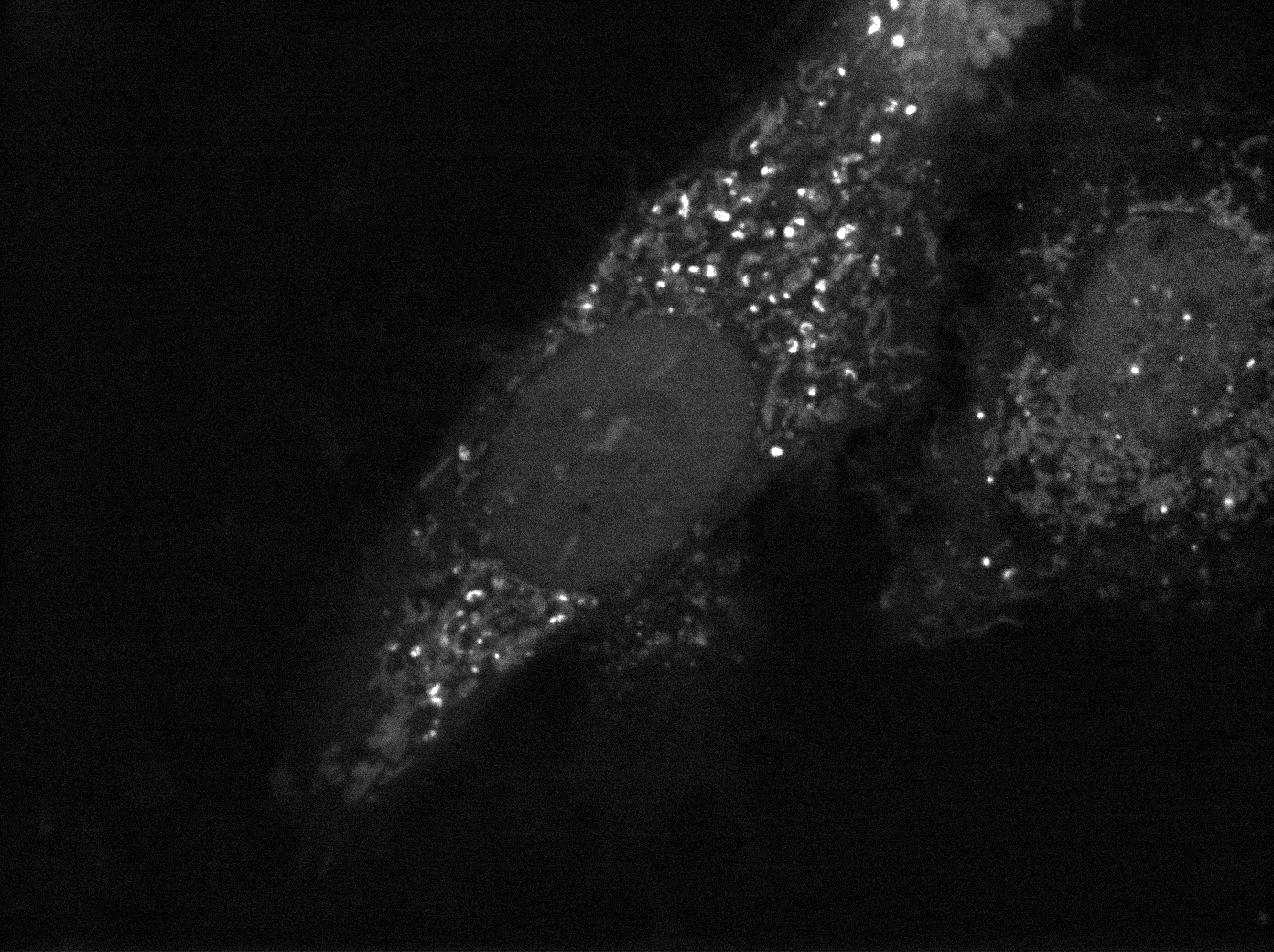

Supplement: Supplementary file 10 — EV and Appendix Figure Source Data [file 44318_2025_454_MOESM10_ESM.zip › EMBOJ-2024-119002R1-EV_and_Appendix_Figures_Source_Data-sd/Fig. EVs source file.zip/Fig EV5/EV5 F/AnxA5KO_Cisplatin_VBIT4.tif]

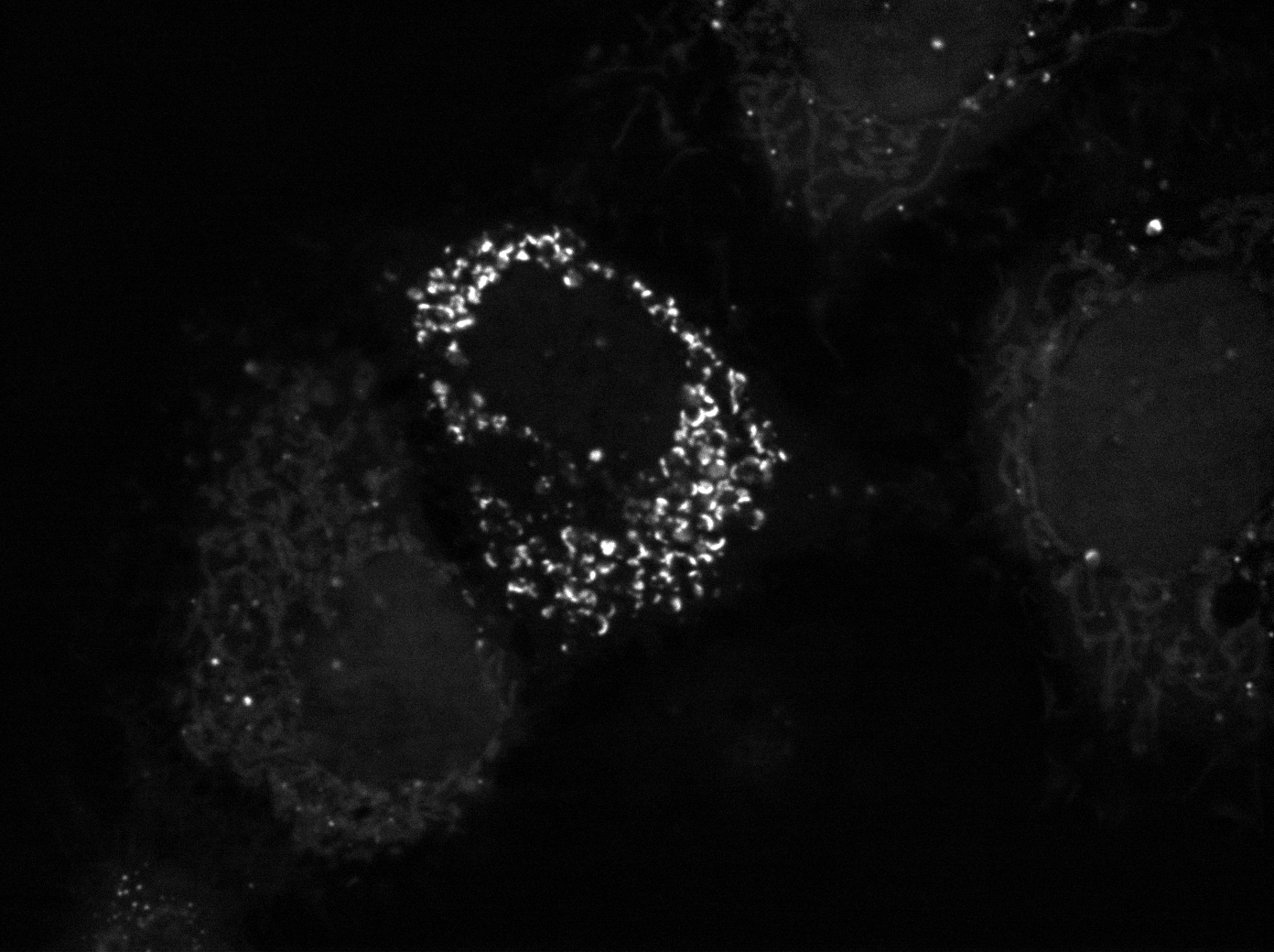

Supplement: Supplementary file 10 — EV and Appendix Figure Source Data [file 44318_2025_454_MOESM10_ESM.zip › EMBOJ-2024-119002R1-EV_and_Appendix_Figures_Source_Data-sd/Fig. EVs source file.zip/Fig EV5/EV5 F/AnxA5KO_Cisplatin.tif]

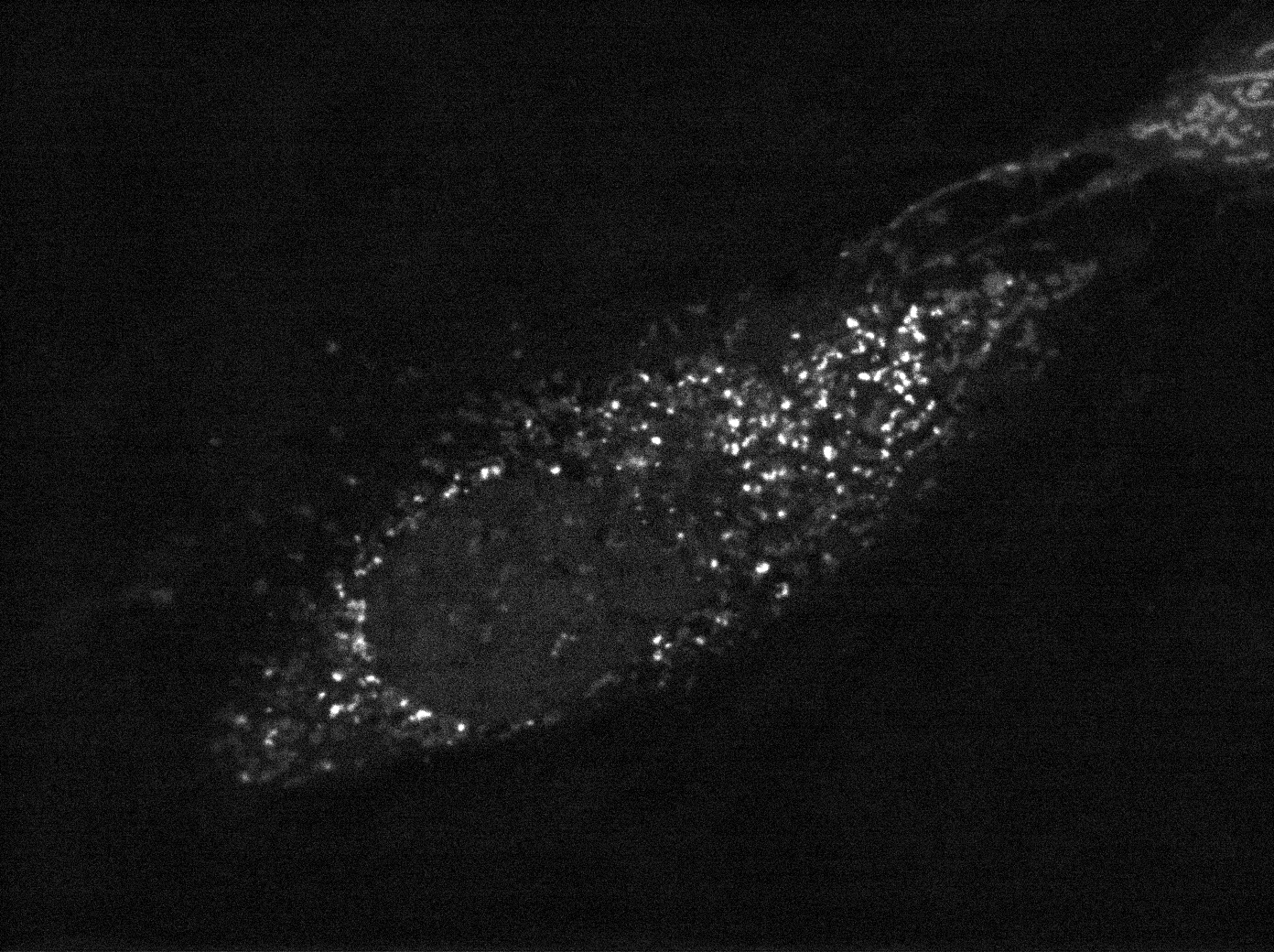

Supplement: Supplementary file 10 — EV and Appendix Figure Source Data [file 44318_2025_454_MOESM10_ESM.zip › EMBOJ-2024-119002R1-EV_and_Appendix_Figures_Source_Data-sd/Fig. EVs source file.zip/Fig EV5/EV5 F/WT__Cisplatin_VBIT4.tif]

All blots are probed with VDAC1 antibody

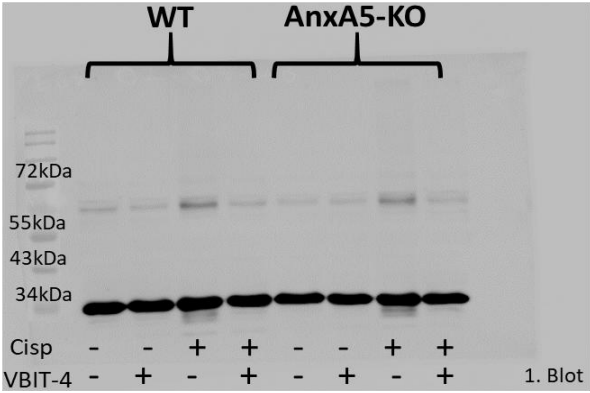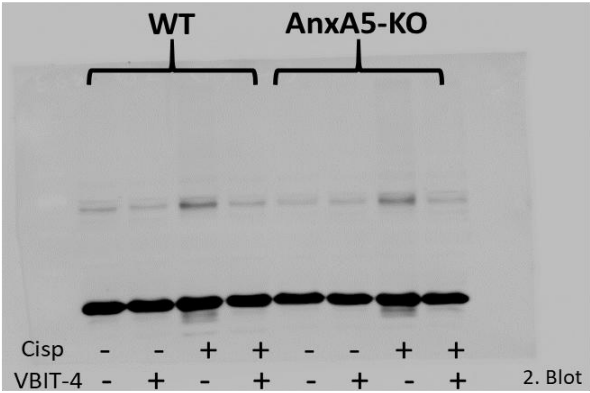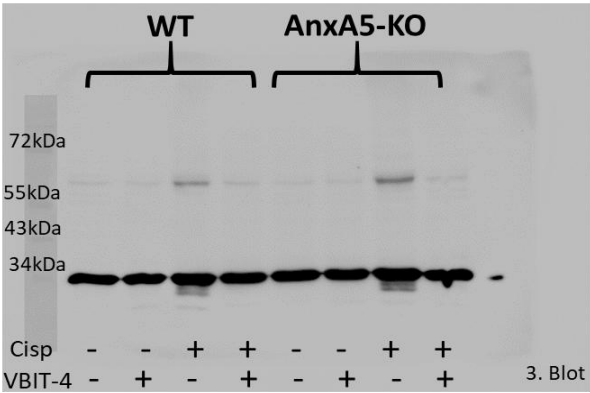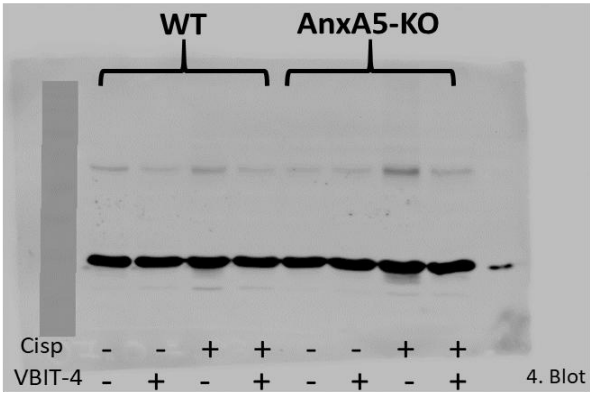

Supplement: Supplementary file 10 — EV and Appendix Figure Source Data [file 44318_2025_454_MOESM10_ESM.zip › EMBOJ-2024-119002R1-EV_and_Appendix_Figures_Source_Data-sd/Fig. EVs source file.zip/Fig EV5/EV5 A/Fig EV5A.pdf]
