# Supplementary material for: Annexin A5 controls VDAC1-dependent mitochondrial Ca2+ homeostasis and determines cellular susceptibility to apoptosis
Source: EMBO J. 2025 May 9;44(12):3413–47. doi: 10.1038/s44318-025-00454-9 (PMC12170872; doi:10.1038/s44318-025-00454-9)
Supplement: Supplementary file 5 — Source data Fig. 3 [file 44318_2025_454_MOESM5_ESM.zip › Figure 3/3A/Figure 3A all blots.pdf]

n=1

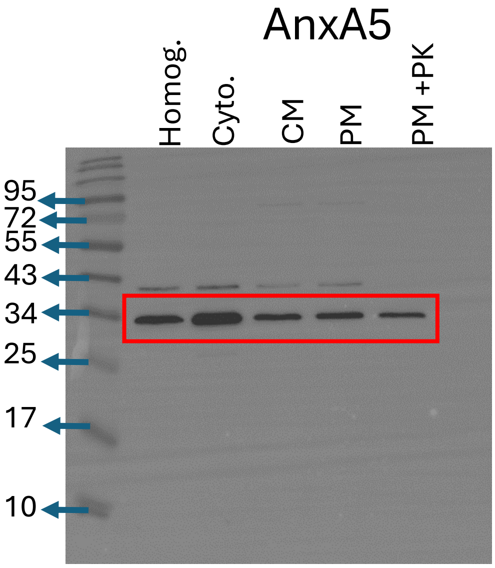

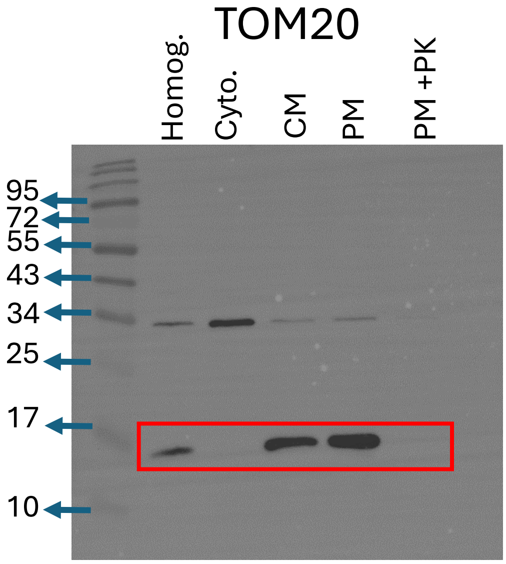

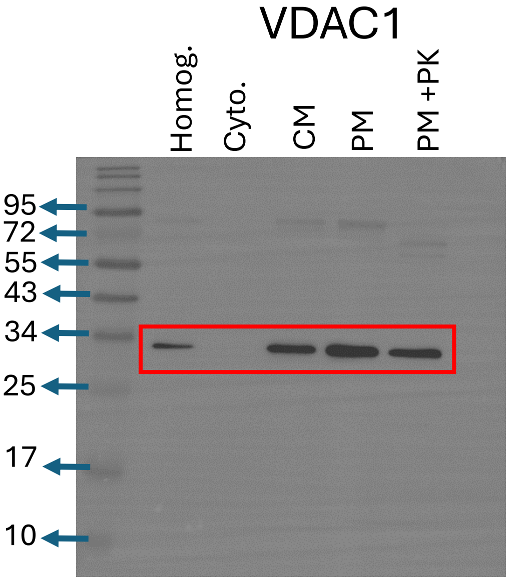

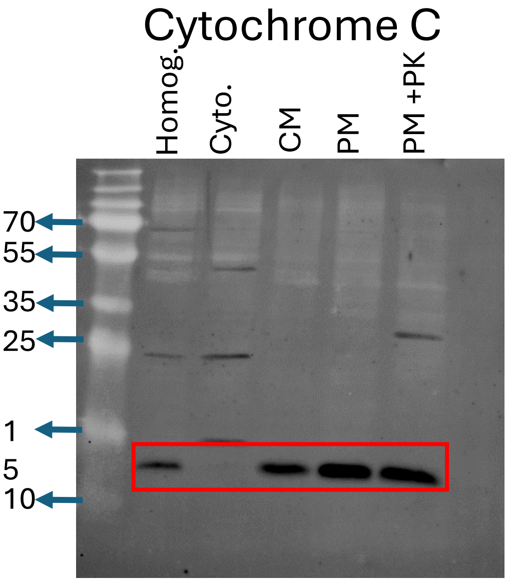

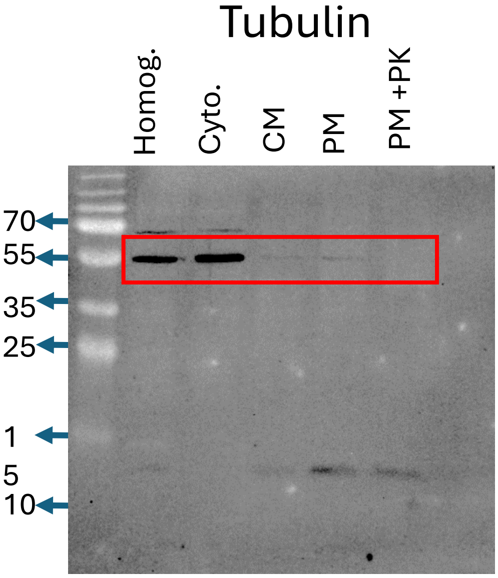

The AnxA5 blot was stripped and then probed with a TOM20 antibody. Similarly, the Cytochrome C blot was stripped and reprobed with Tubulin and VDAC1 antibodies. The blots on this page were used for Figure 3A.

Homog. = Homogenate

Cyto = Cytosolic fraction

CM = Crude mitochondria

PM = Pure mitochondira

PM+PK = Pure mitochondira + proteinase K

n=2

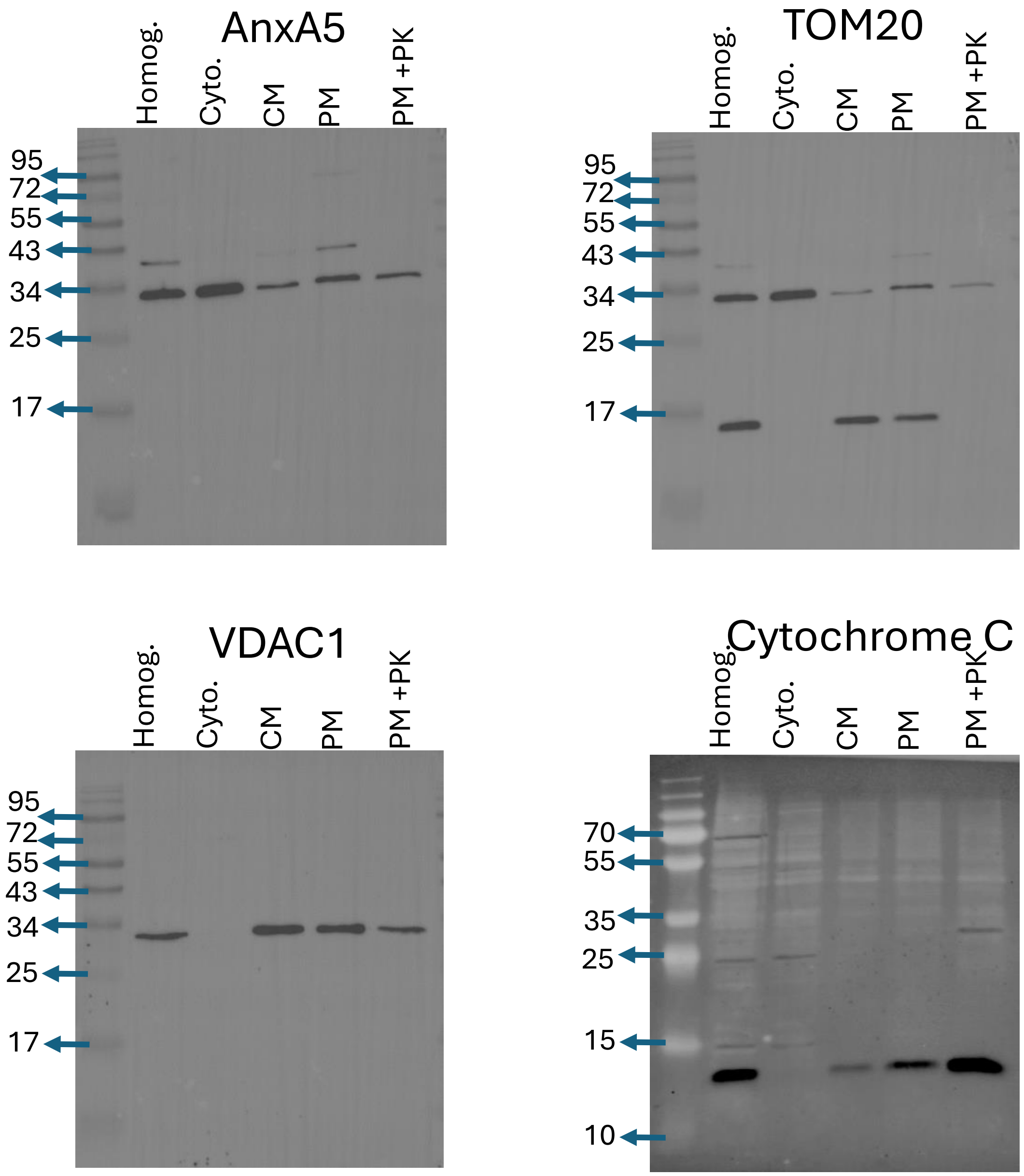

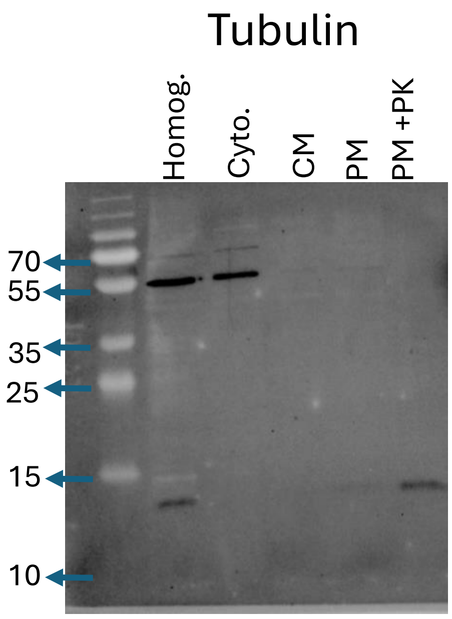

The AnxA5 blot was stripped and then probed with a TOM20 antibody. Similarly, the Cytochrome C blot was stripped and reprobed with Tubulin and VDAC1 antibodies.
Homog. = Homogenate
Cyto = Cytosolic fraction
CM = Crude mitochondria
PM = Pure mitochondira
PM+PK = Pure mitochondira + proteinase K

n=3

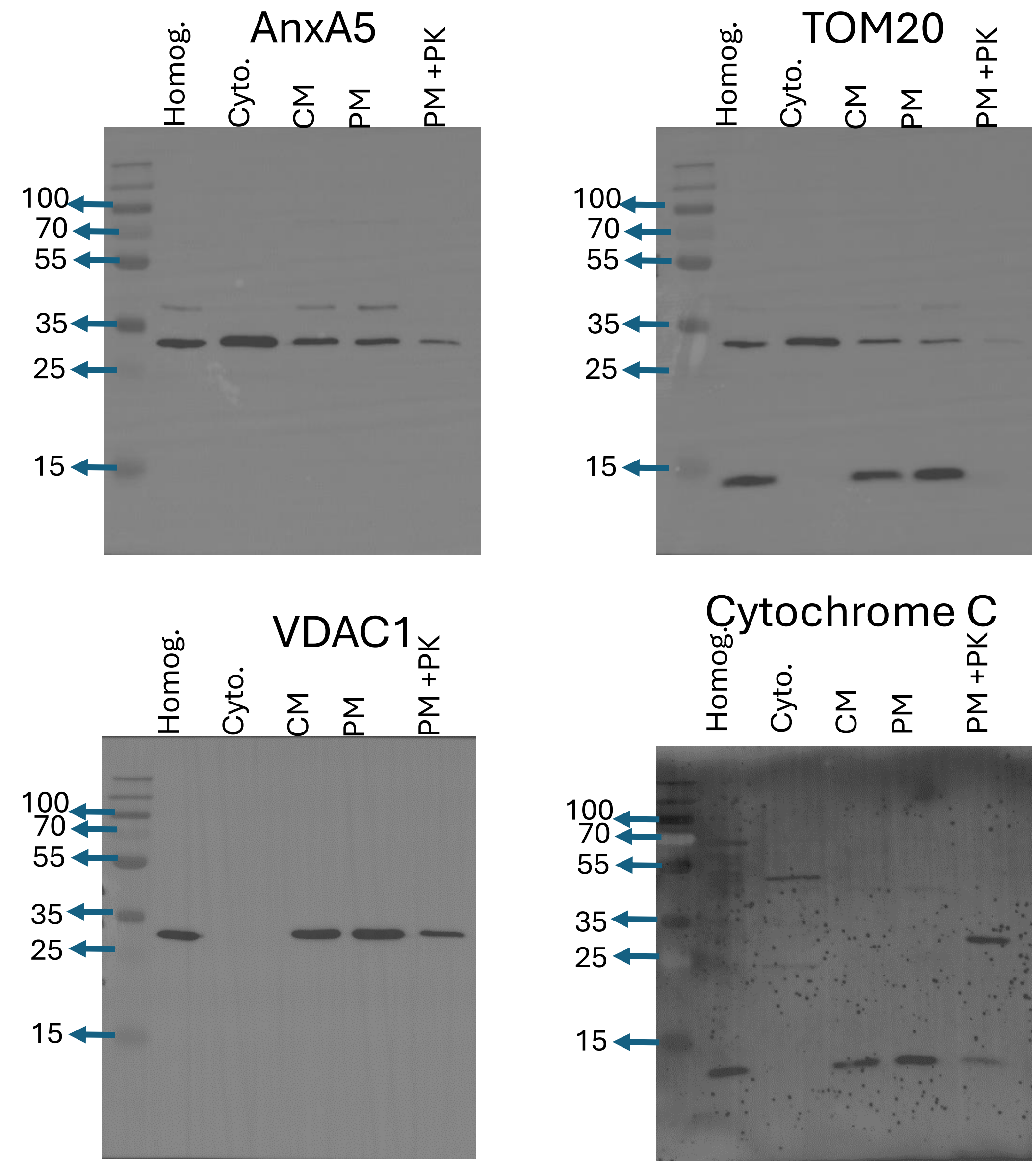

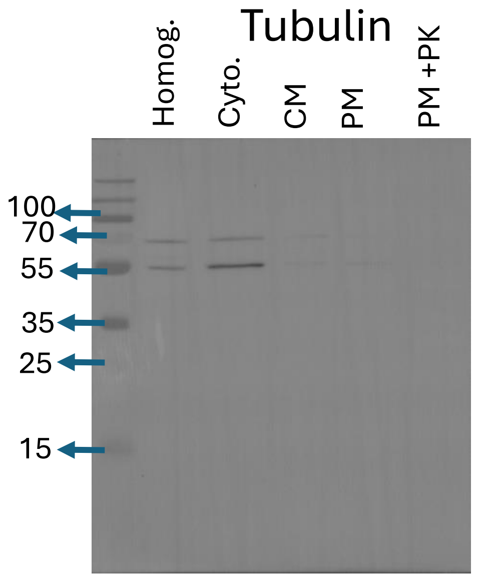

The AnxA5 blot was stripped and then probed with a TOM20 antibody. Similarly, the Cytochrome C blot was stripped and reprobed with Tubulin and VDAC1 antibodies. Homog. = Homogenate
Cyto = Cytosolic fraction
CM = Crude mitochondria
PM = Pure mitochondira
PM+PK = Pure mitochondira + proteinase K
