# Supplementary material for: Annexin A5 controls VDAC1-dependent mitochondrial Ca2+ homeostasis and determines cellular susceptibility to apoptosis
Source: EMBO J. 2025 May 9;44(12):3413–47. doi: 10.1038/s44318-025-00454-9 (PMC12170872; doi:10.1038/s44318-025-00454-9)

Figure 3a, uncropped version of upper right image

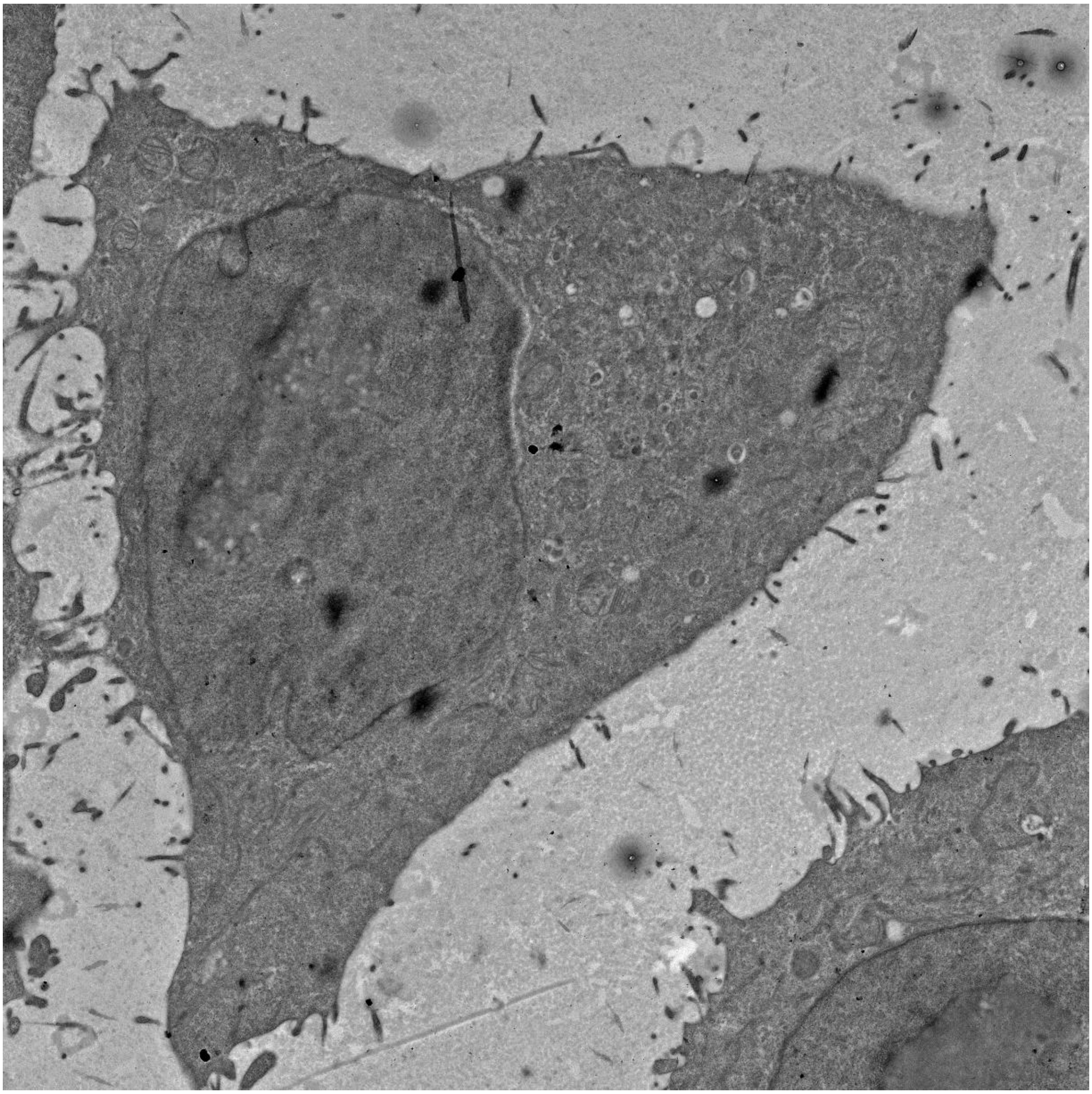

Figure 3a, uncropped version of upper left image

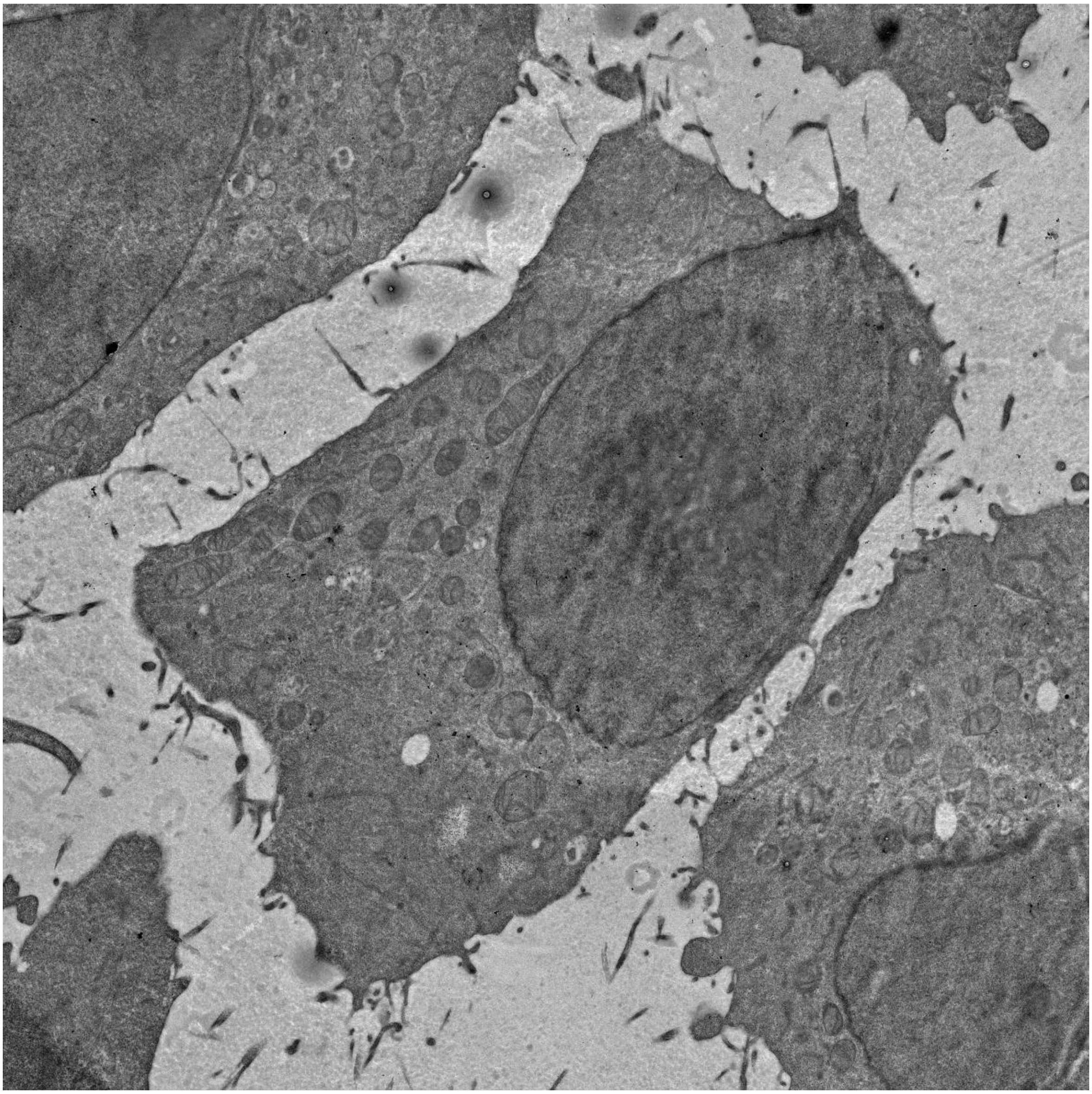

Figure 3a, uncropped version of lower left image

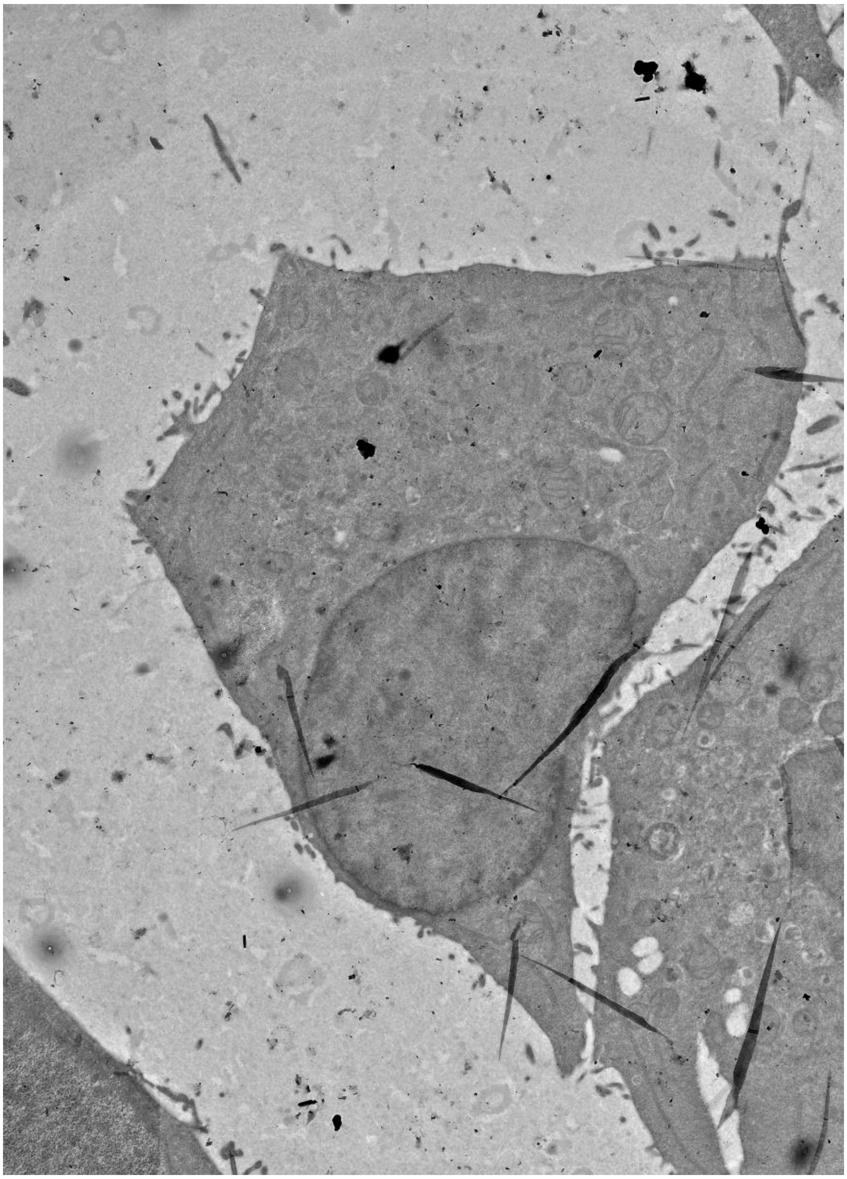

Figure 3a, uncropped version of lower right image

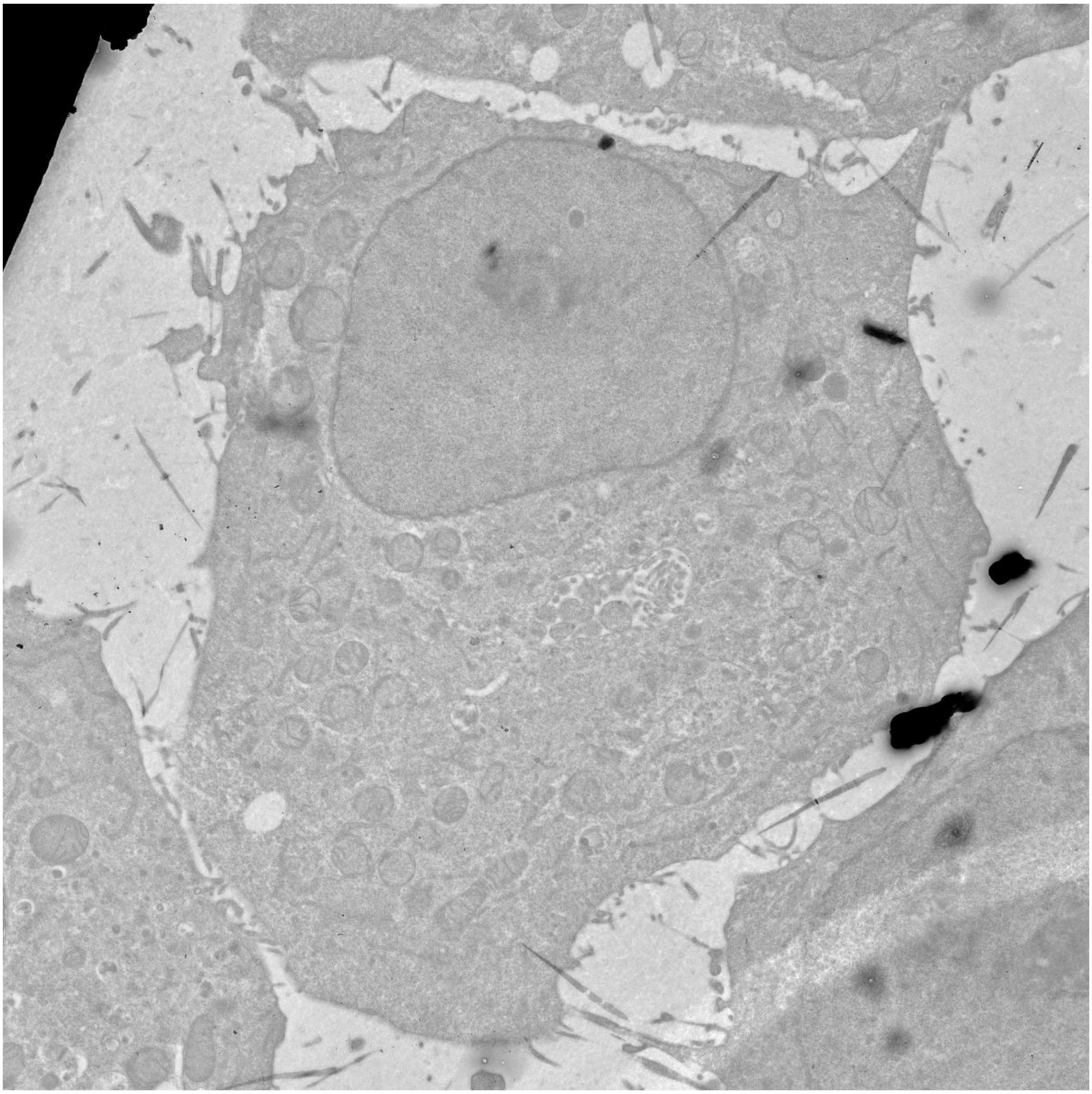

Supplement: Supplementary file 5 — Source data Fig. 3 [file 44318_2025_454_MOESM5_ESM.zip › Figure 3/3B/Uncropped version of Figure 3b.pdf]
