# Supplementary material for: Annexin A5 controls VDAC1-dependent mitochondrial Ca2+ homeostasis and determines cellular susceptibility to apoptosis
Source: EMBO J. 2025 May 9;44(12):3413–47. doi: 10.1038/s44318-025-00454-9 (PMC12170872; doi:10.1038/s44318-025-00454-9)

Both representative and replicate experiments for Figures 7H and 7I are shown. The first blot was used in Figure 7H, and all four blots were quantified and presented in Figure 7I.

VDAC1 antibody was used for all four blots.

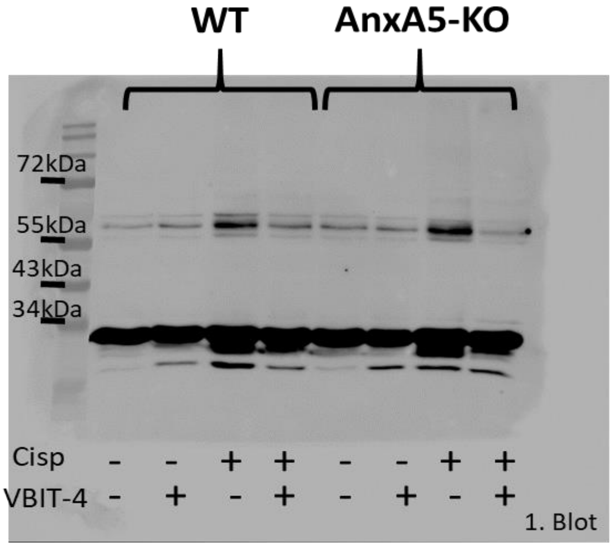

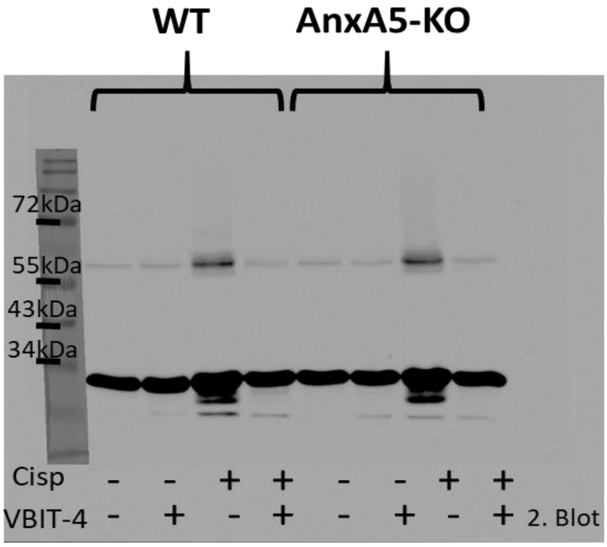

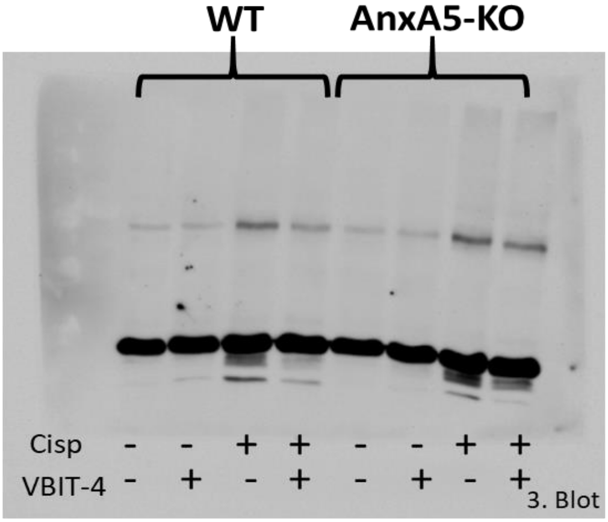

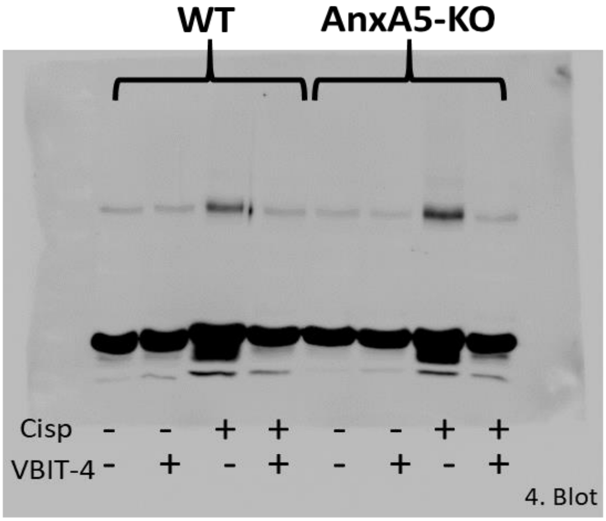

Supplement: Supplementary file 8 — Source data Fig. 7 [file 44318_2025_454_MOESM8_ESM.zip › Figure 7/7H/VDAC1_blots_cisplatin_treatment.pdf]
