# Supplementary material for: Annexin A5 controls VDAC1-dependent mitochondrial Ca2+ homeostasis and determines cellular susceptibility to apoptosis
Source: EMBO J. 2025 May 9;44(12):3413–47. doi: 10.1038/s44318-025-00454-9 (PMC12170872; doi:10.1038/s44318-025-00454-9)
Supplement: Supplementary file 10 — EV and Appendix Figure Source Data [file 44318_2025_454_MOESM10_ESM.zip › EMBOJ-2024-119002R1-EV_and_Appendix_Figures_Source_Data-sd/Appendix Figure S3J/Appendix Figure S3J. blots.pdf]

Cells were treated with 5 and 10 μM of cisplatin for 12, 24, and 48 hours. To better capture the dimeric VDAC1 level, two different Ethylene Glycol Bis(Succinimidyl Succinate) concentrations (150 and 300 μM) were used. **Blots were then probed with a VDAC1 antibody.**

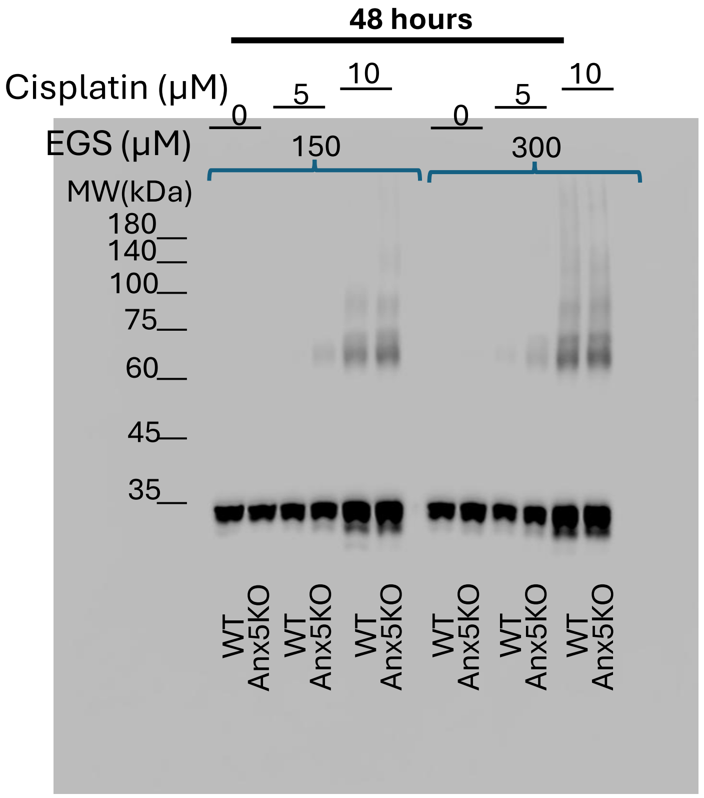

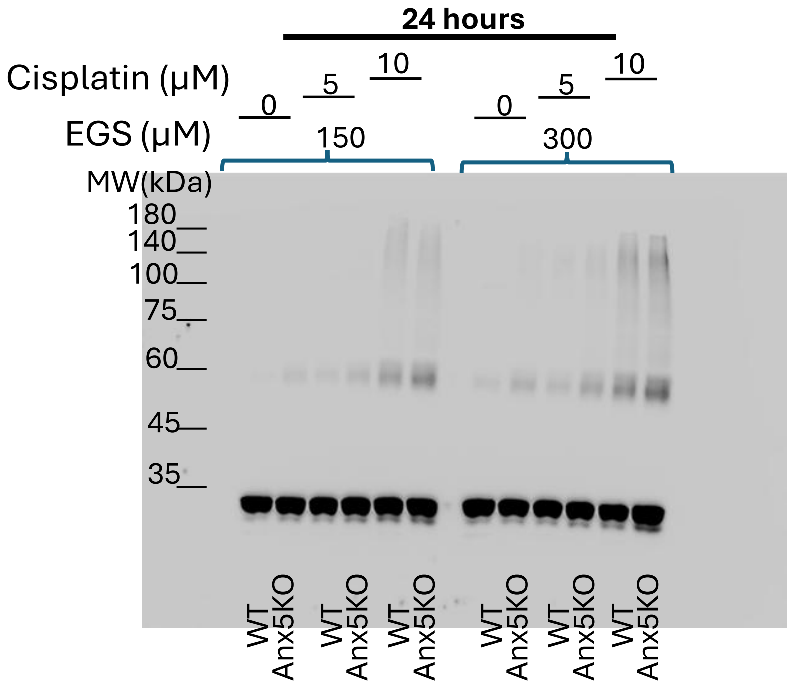

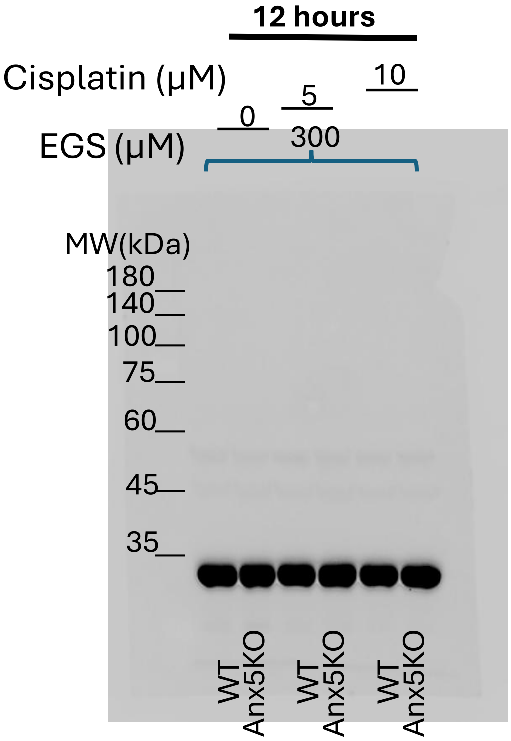

12 hours
Cisplatin (μM)
0
5
10
EGS (μM)
300
MW(kDa)
180
140
100
75
60
45
35
WT
Anx5KO
WT
Anx5KO
WT
Anx5KO
