# Supplementary material for: Annexin A5 controls VDAC1-dependent mitochondrial Ca2+ homeostasis and determines cellular susceptibility to apoptosis
Source: EMBO J. 2025 May 9;44(12):3413–47. doi: 10.1038/s44318-025-00454-9 (PMC12170872; doi:10.1038/s44318-025-00454-9)
Supplement: Supplementary file 10 — EV and Appendix Figure Source Data [file 44318_2025_454_MOESM10_ESM.zip › EMBOJ-2024-119002R1-EV_and_Appendix_Figures_Source_Data-sd/Fig. EVs source file.zip/Fig EV5/EV5 C/Fig. EV5C.pdf]

Blots are probed with VDAC1 Antibody

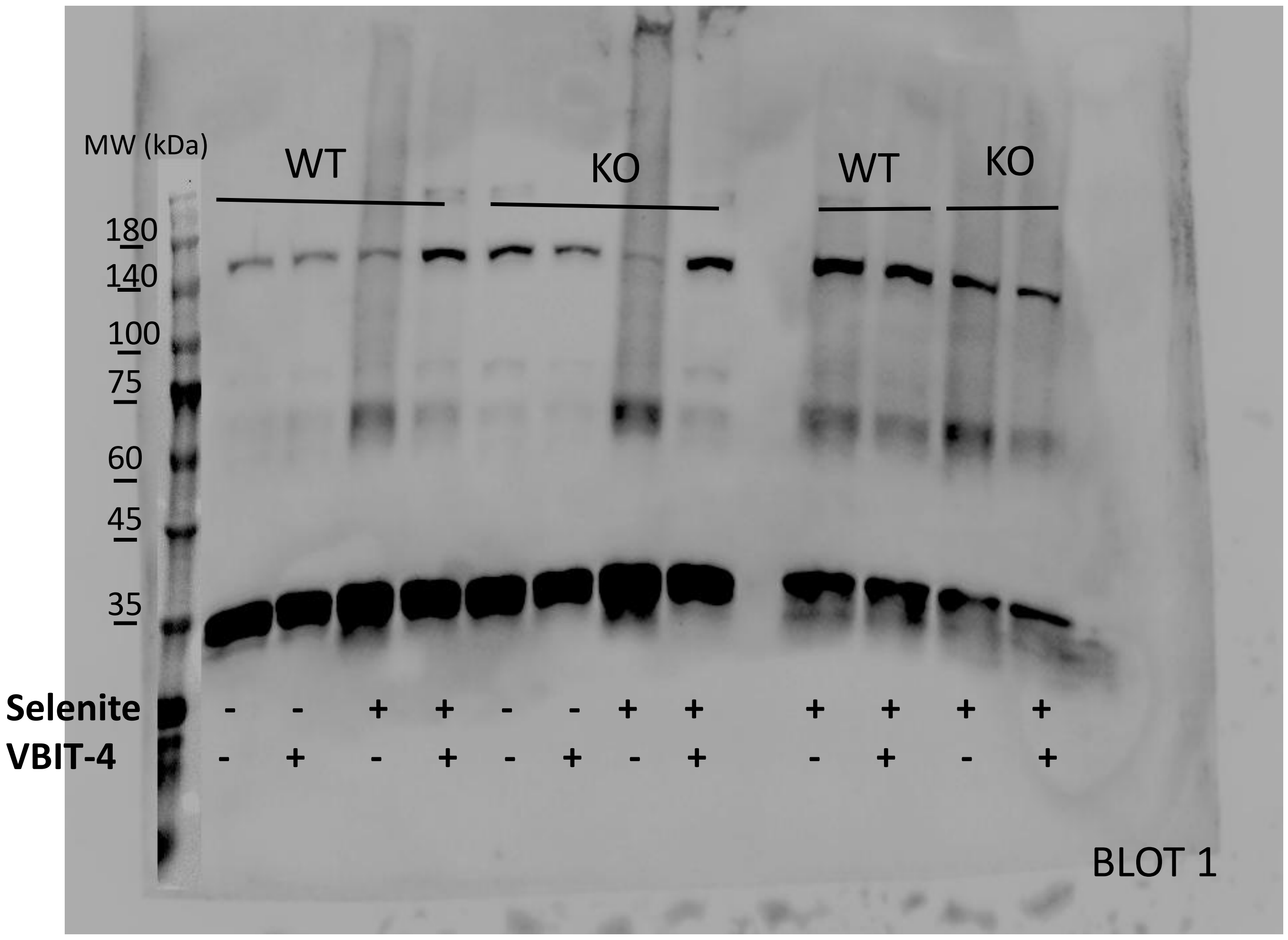

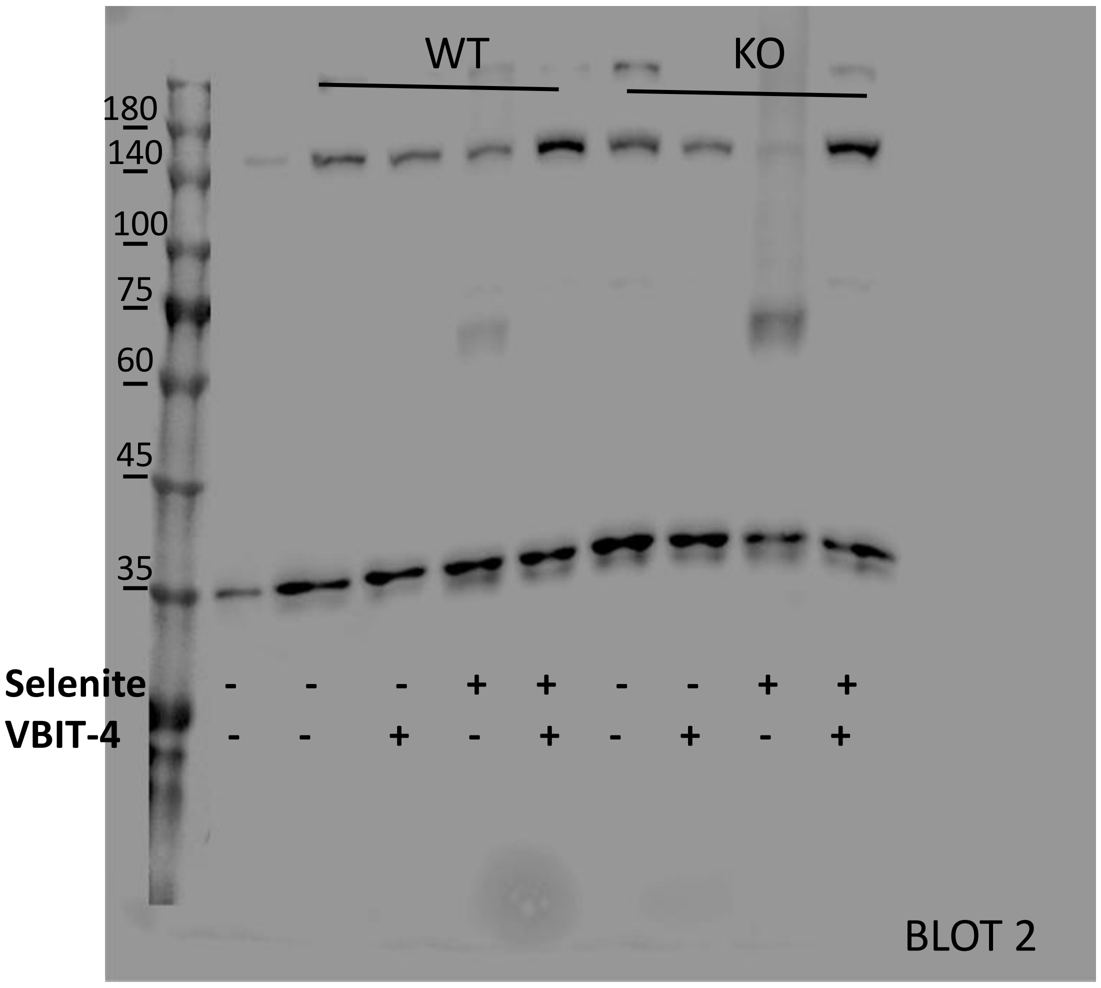

The dimeric levels of VDAC1 are calculated based on the bands that fall between 60 and 75 kDa.
