# Supplementary material for: Annexin A5 controls VDAC1-dependent mitochondrial Ca2+ homeostasis and determines cellular susceptibility to apoptosis
Source: EMBO J. 2025 May 9;44(12):3413–47. doi: 10.1038/s44318-025-00454-9 (PMC12170872; doi:10.1038/s44318-025-00454-9)
Supplement: Supplementary file 10 — EV and Appendix Figure Source Data [file 44318_2025_454_MOESM10_ESM.zip › EMBOJ-2024-119002R1-EV_and_Appendix_Figures_Source_Data-sd/Fig. EVs source file.zip/Fig EV2/EV2 J/Fig EV2J.pdf]

N=1

AnxA5 (35 kDa) Antibody

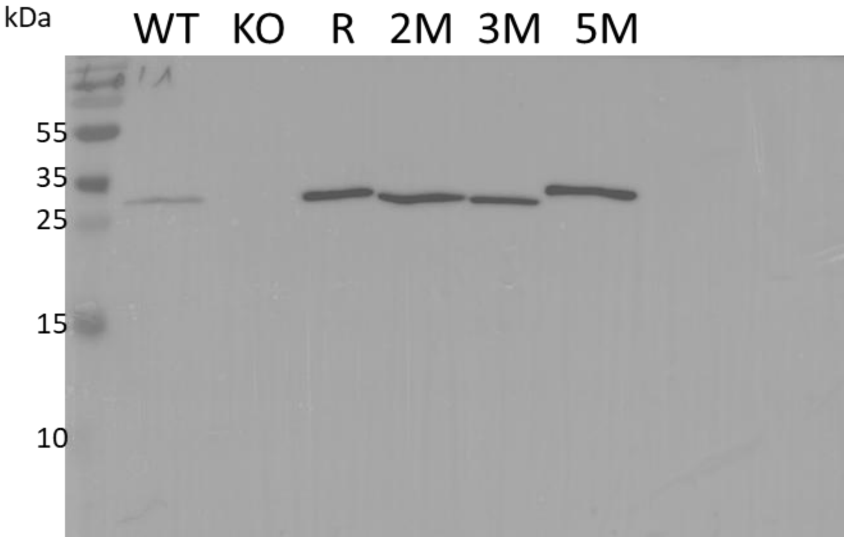

Histone H3 (15 kDa) Antibody

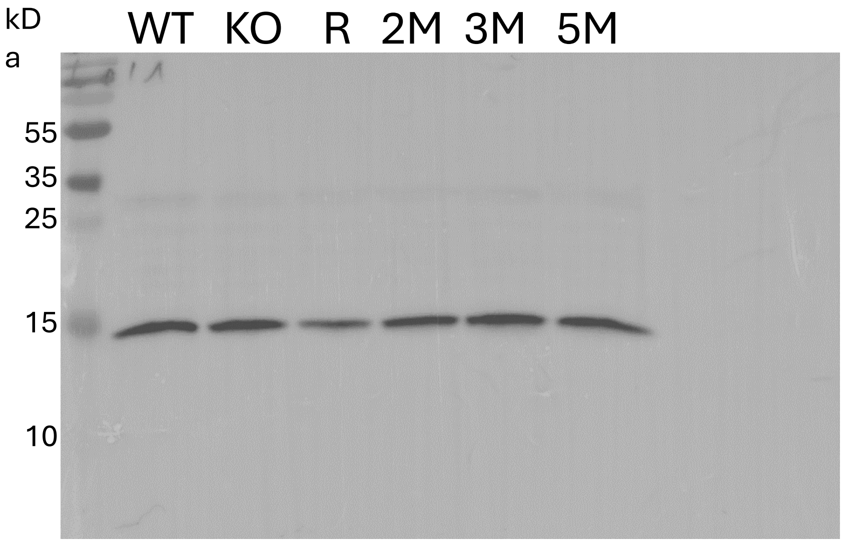

AnxA5 blot was stripped and incubated with Histone H3 antibody

WT = HeLa cells
KO = AnxA5-KO cells
R = AnxA5-KO cells transfected with WT AnxA5
2M = AnxA5-KO cells transfected with AnxA5-2Mt
3M = AnxA5-KO cells transfected with AnxA5-3Mt
5M = AnxA5-KO cells transfected with AnxA5-5Mt

N=2

AnxA5 (35 kDa) Antibody

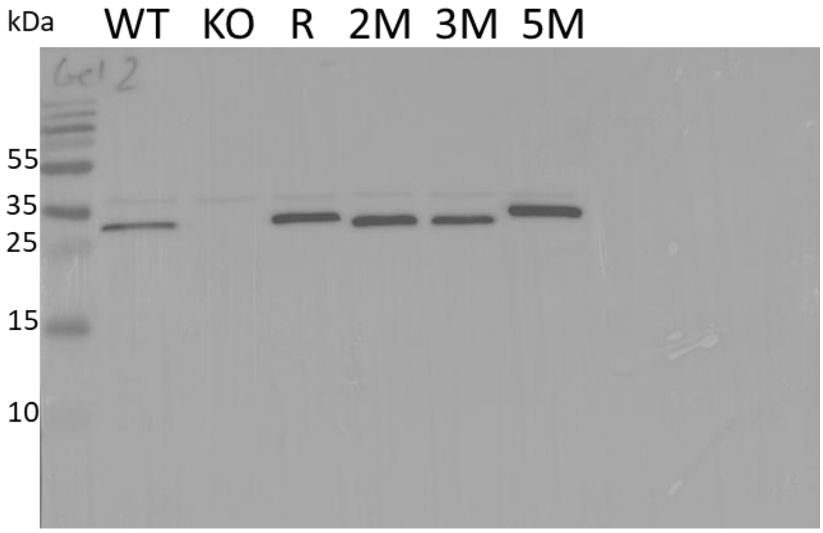

Histone H3 (15 kDa) Antibody

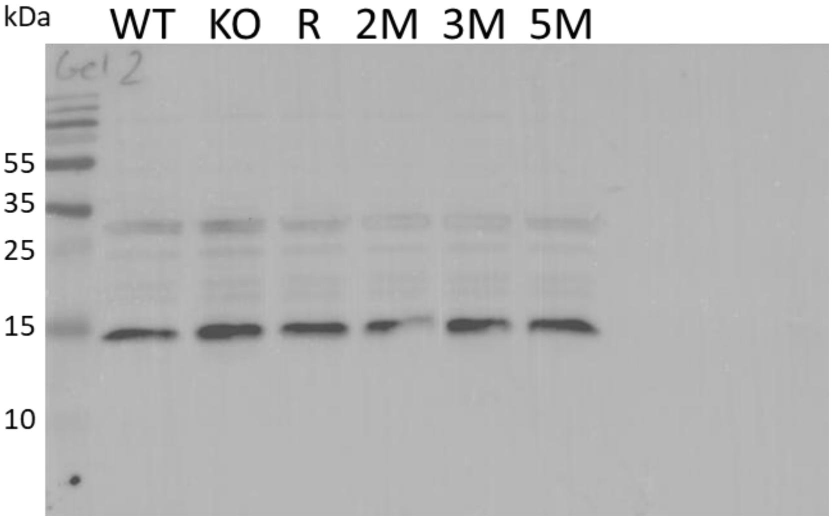

AnxA5 blot was stripped and incubated with Histone H3 antibody

WT = HeLa cells
KO = AnxA5-KO cells
R = AnxA5-KO cells transfected with WT AnxA5
2M = AnxA5-KO cells transfected with AnxA5-2Mt
3M = AnxA5-KO cells transfected with AnxA5-3Mt
5M = AnxA5-KO cells transfected with AnxA5-5Mt
