# Supplementary material for: Annexin A5 controls VDAC1-dependent mitochondrial Ca2+ homeostasis and determines cellular susceptibility to apoptosis
Source: EMBO J. 2025 May 9;44(12):3413–47. doi: 10.1038/s44318-025-00454-9 (PMC12170872; doi:10.1038/s44318-025-00454-9)
Supplement: Supplementary file 11 — Expanded View Figures [file 44318_2025_454_MOESM11_ESM.pdf]

Expanded View Figures

Figure EV1. Characterization of AnxA5-KO cells.

(**A**) Immunoblots show the AnxA5 expression in WT and AnxA5-KO in HeLa and (**B**) EA.hy926 cells (Experiments are performed in Clone 21 indicated as a red rectangle). Uncropped blots are provided in the Source data. Panel (**A**) shows a redisplay of content from Fig. EV2J. (**C**) Graphical representation of genetically encoded FRET-based mitochondrial matrix targeted Ca^{2+} sensor (4mtD3cpv). (**D**) Representative image of HeLa cells transfected with (4mtD3cpv). The cells have been pseudocolored to represent mitochondrial Ca^{2+} levels as a ratio under basal (left panel) conditions or upon histamine stimulation (left panel) (Scale bar = 5 μm). (**E**) Average time courses of the 100 μM histamine-induced $[Ca^{2+}]_{Matrix}$ responses in WT (black) and AnxA5-KO (red) in EA.hy926 cells measured in Ca^{2+}-free buffer (containing 100 μM EGTA). (**F**) Bar graphs show the basal $[Ca^{2+}]_{Matrix}$ and (**G**) histamine-induced maximum $[Ca^{2+}]_{Matrix}$ levels in WT (black) and AnxA5-KO (red). Data points represent the mean ± SEM ($n_{WT} = 9/6$; $n_{AnxA5\text{-}KO} = 12/6$). The *p*-value for (**G**) is $p = 0.0011$ ($^{**}p < 0.01$). (**H**) Mean time courses of the histamine-induced $[Ca^{2+}]_{Cyto}$ responses in WT (black) and AnxA5-KO (red) in EA.hy926 cells measured in Ca^{2+}-free buffer (containing 100 μM EGTA). (**I**) Bar graphs show the basal $[Ca^{2+}]_{Cyto}$ and (**J**) histamine-induced maximum $[Ca^{2+}]_{Cyto}$ levels in WT (black) and AnxA5-KO (red). Data points represent the mean ± SEM ($n_{WT} = 101/6$; $n_{AnxA5\text{-}KO} = 88/6$). The *p*-value for (**J**) is $p = 0.0094$ ($^{**}p < 0.01$). (**K**) Representative Immunoblot shows the expression level of AnxA5 transfected either with shControl or shAnxA5. Uncropped blots are provided in the Source Data. (**L**) Bar graph represents immunoblot analysis of AnxA5 expression as mean ± SEM ($n_{shControl} = 3$; $n_{shAnxA5} = 3$). (**M**) Average time courses of the 100 μM histamine-induced $[Ca^{2+}]_{Matrix}$ responses in the presence (dashed lines) and absence (solid lines) of CGP37157 in shControl (black) and shAnxA5 (red) in HeLa cells. Data points represent the mean ± SEM ($n_{shControl} = 29/3$; $n_{shAnxA5} = 28/4$; $n_{shControl\text{-}CGP37157} = 56/3$; $n_{shAnxA5\text{-}CGP37157} = 43/4$). Significant differences were assessed with the two-tailed unpaired Student's t-test ($^{**}p < 0.01$ and ns: not significant). Source data are available online for this figure.

 © The Author(s)

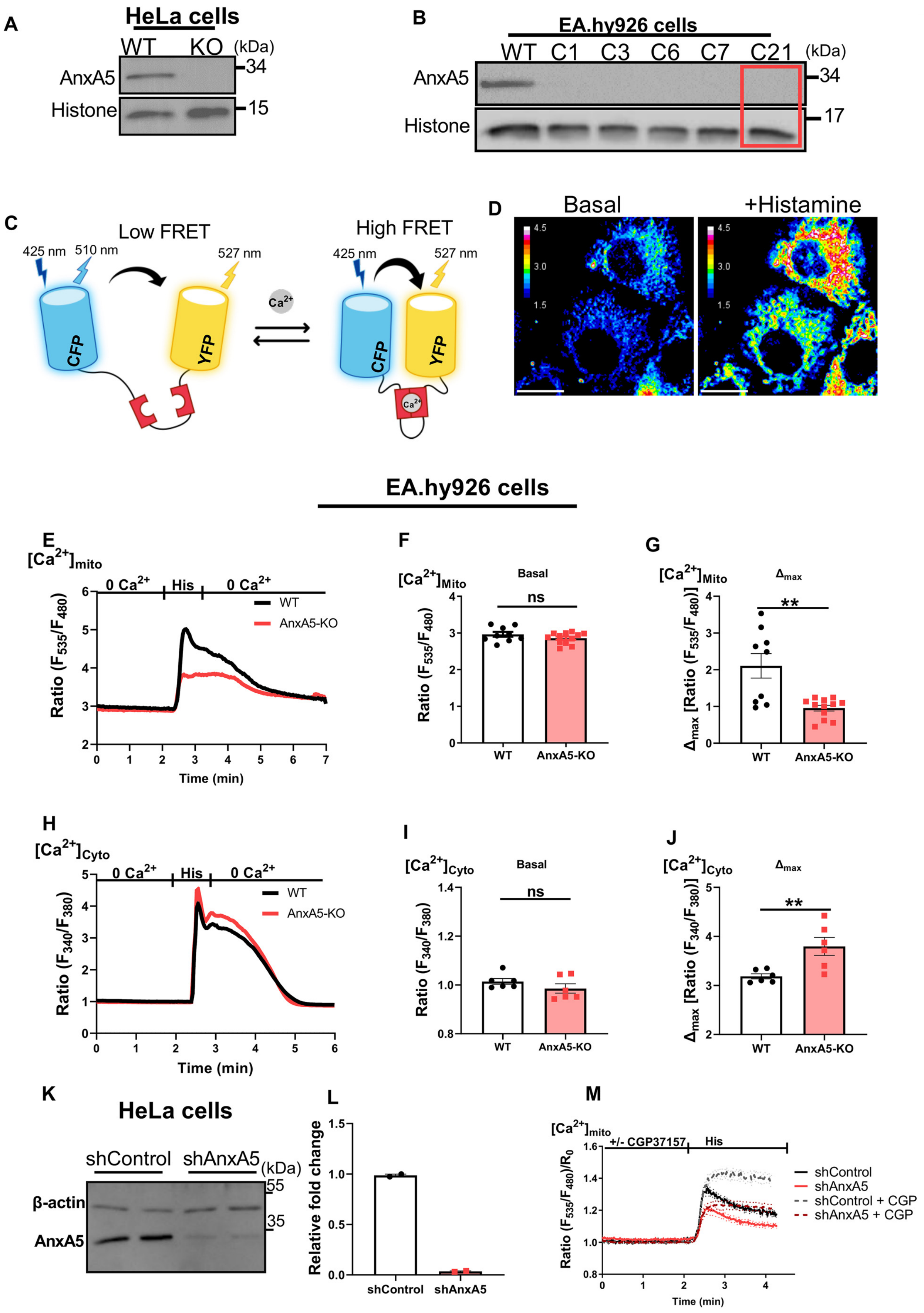
A
HeLa cells
WT KO (kDa)
AnxA5 34
Histone 15
B
EA.hy926 cells
WT C1 C3 C6 C7 C21 (kDa)
AnxA5 34
Histone 17
C
Low FRET
High FRET
425 nm 510 nm 527 nm
425 nm 527 nm
CFP
YFP
Ca2+
D
Basal
+Histamine
4.5
3.0
1.5
EA.hy926 cells
E
[Ca2+]mito
0 Ca2+ His 0 Ca2+
WT
AnxA5-KO
Ratio (F535/F480)
Time (min)
F
[Ca2+]Mito
Basal
ns
Ratio (F535/F480)
WT AnxA5-KO
G
[Ca2+]Mito
Δmax
**
Δmax [Ratio (F535/F480)]
WT AnxA5-KO
H
[Ca2+]Cyto
0 Ca2+ His 0 Ca2+
WT
AnxA5-KO
Ratio (F340/F380)
Time (min)
I
[Ca2+]Cyto
Basal
ns
Ratio (F340/F380)
WT AnxA5-KO
J
[Ca2+]Cyto
Δmax
**
Δmax [Ratio (F340/F380)]
WT AnxA5-KO
K
HeLa cells
shControl shAnxA5 (kDa)
β-actin 55
AnxA5 35
L
Relative fold change
shControl shAnxA5
M
[Ca2+]mito
+/- CGP37157 His
Ratio (F535/F480)/R0
Time (min)
shControl
shAnxA5
shControl + CGP
shAnxA5 + CGP

© The Author(s)

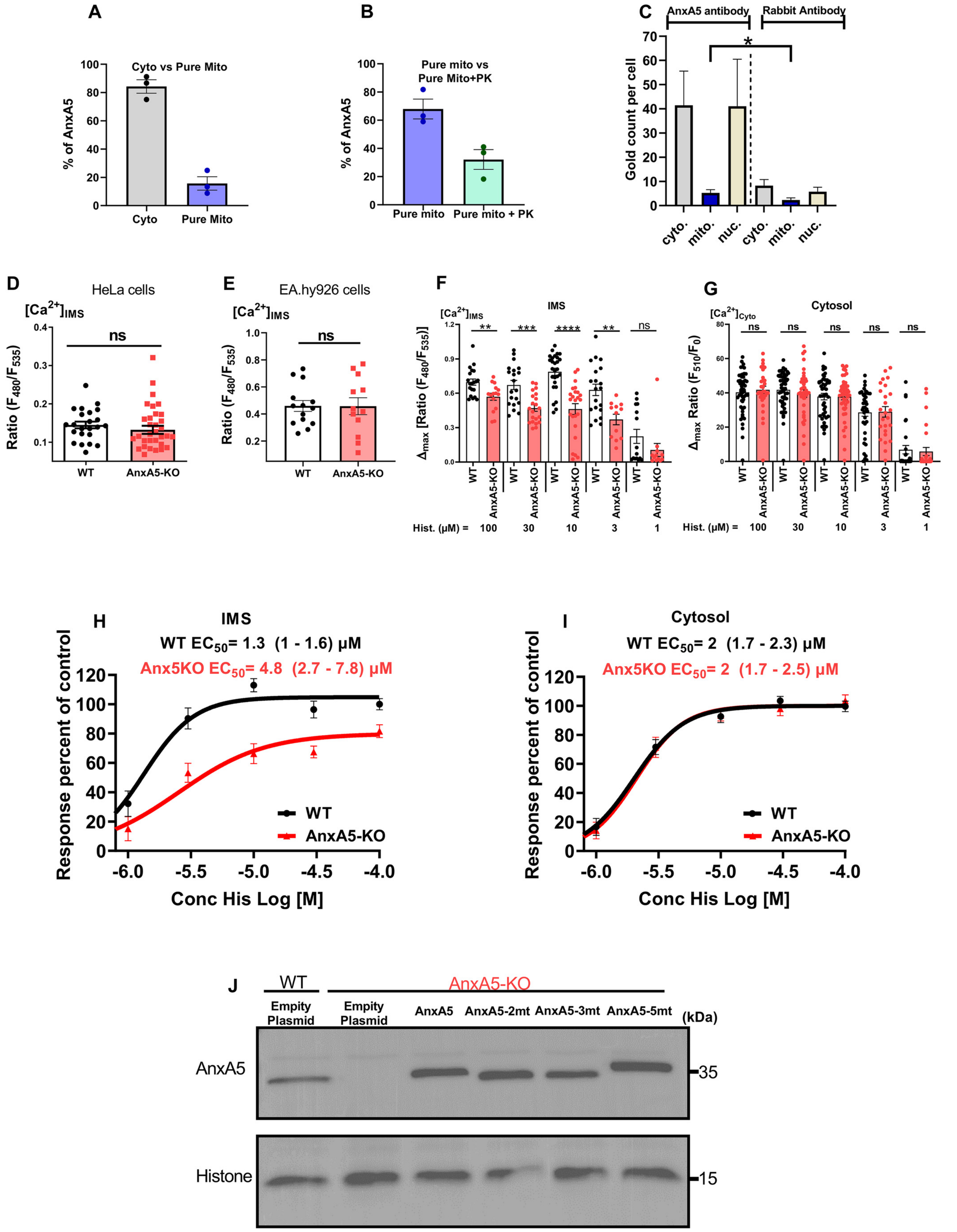
A
Cyto vs Pure Mito
% of AnxA5
Cyto
Pure Mito
B
Pure mito vs Pure Mito+PK
Pure mito
Pure mito + PK
C
AnxA5 antibody
Rabbit Antibody
Gold count per cell
cyto.
mito.
nuc.
D
HeLa cells
$[Ca^{2+}]_{IMS}$
ns
Ratio (F_{480}/F_{535})
WT
AnxA5-KO
E
EA.hy926 cells
$[Ca^{2+}]_{IMS}$
ns
F
IMS
$[Ca^{2+}]_{IMS}$
Δ_{max} [Ratio (F_{480}/F_{535})]
Hist. (μM) = 100 30 10 3 1
G
Cytosol
$[Ca^{2+}]_{Cyto}$
Δ_{max} Ratio (F_{510}/F_0)
H
IMS
WT EC_{50}= 1.3 (1 - 1.6) μM
Anx5KO EC_{50}= 4.8 (2.7 - 7.8) μM
Response percent of control
Conc His Log [M]
WT
AnxA5-KO
I
Cytosol
WT EC_{50}= 2 (1.7 - 2.3) μM
Anx5KO EC_{50}= 2 (1.7 - 2.5) μM
J
WT
AnxA5-KO
Empity Plasmid
Empity Plasmid
AnxA5
AnxA5-2mt
AnxA5-3mt
AnxA5-5mt
(kDa)
AnxA5
Histone
35
15

 © The Author(s)

Figure EV2. AnxA5 localizes on and within the mitochondria and regulates IMS Ca^{2+} signaling.

(**A**) Bar graphs show the calculated percentage localization of AnxA5 in cytosol versus pure mitochondria, (**B**) pure mitochondria versus pure mitochondria + proteinase K (PK) treatment in mitochondria isolated from HeLa cells. Data points represent the mean ± SEM in HeLa cells ($n = 3$). (**C**) Bar graphs show the distribution of gold particles in cytosol, mitochondria, and the nucleus using AnxA5 antibody or rabbit antibody as a negative control. Data points represent the mean ± SEM in HeLa ($n_{AnxA5\ antibody} = 13/3$; $n_{Rabbit\ antibody} = 15/3$). The *p*-value is $p = 0.0337$ ($*p < 0.05$). (**D**) Bar graphs show the basal $[Ca^{2+}]_{IMS}$ levels in WT (black), AnxA5-KO (red), and Rescue (blue) in HeLa and (**E**) EA.hy926 cells. Data points represent the mean ± SEM in HeLa ($n_{WT} = 23/7$; $n_{AnxA5\text{-}KO} = 34/8$; $n_{Rescue} = 30/9$) and in EA.hy926 cells ($n_{WT} = 14/6$; $n_{AnxA5\text{-}KO} = 12/6$). (**F**) Bar graphs show a histamine-induced maximum $[Ca^{2+}]_{IMS}$ and (**G**) $[Ca^{2+}]_{cyto}$ elevation in WT (black) and AnxA5-KO (red) HeLa cells measured in Ca^{2+}-free buffer. The *p*-values for (**F**), from left to right, are: $p = 0.0037$ ($**p < 0.01$), $p = 0.0002$ ($***p < 0.001$), $p < 0.0001$ ($****p < 0.0001$), $p = 0.0014$ ($**p < 0.01$), and $p = 0.1758$ (ns). (**H**) Concentration-response curve of histamine (1, 3, 10, 30, 100 µM) shows maximum $[Ca^{2+}]_{IMS}$ and (**I**) $[Ca^{2+}]_{Cyto}$ rise in WT (black) and AnxA5-KO (red) cells measured in Ca^{2+}-free buffer. The values were calculated from panels (**F**) and (**G**). Data points represent the mean ± SEM in IMS ($n_{WT\text{-}100\ \mu M\text{-}Hist} = 19/4$; $n_{AnxA5\text{-}KO\text{-}100\ \mu M\text{-}Hist} = 14/5$; $n_{WT\text{-}30\mu M\text{-}Hist} = 19/5$; $n_{AnxA5\text{-}KO\text{-}30\ \mu M\text{-}Hist} = 20/5$; $n_{WT\text{-}10\ \mu M\text{-}Hist} = 26/6$; $n_{AnxA5\text{-}KO\text{-}10\ \mu M\text{-}Hist} = 23/6$; $n_{WT\text{-}3\mu M\text{-}Hist} = 19/6$; $n_{AnxA5\text{-}KO\text{-}3\ \mu M\text{-}Hist} = 11/6$; $n_{WT\text{-}1\ \mu M\text{-}Hist} = 14/3$; $n_{AnxA5\text{-}KO\text{-}1\ \mu M\text{-}Hist} = 11/3$) and in the cytosol ($n_{WT\text{-}100\ \mu M\text{-}Hist} = 50/5$; $n_{AnxA5\text{-}KO\text{-}100\ \mu M\text{-}Hist} = 33/4$; $n_{WT\text{-}30\ \mu M\text{-}Hist} = 50/5$; $n_{AnxA5\text{-}KO\text{-}30\ \mu M\text{-}Hist} = 40/5$; $n_{WT\text{-}10\ \mu M\text{-}Hist} = 46/4$; $n_{AnxA5\text{-}KO\text{-}10\ \mu M\text{-}Hist} = 46/4$; $n_{WT\text{-}3\ \mu M\text{-}Hist} = 39/4$; $n_{AnxA5\text{-}KO\text{-}3\ \mu M\text{-}Hist} = 25/4$; $n_{WT\text{-}1\ \mu M\text{-}Hist} = 30/5$; $n_{AnxA5\text{-}KO\text{-}1\ \mu M\text{-}Hist} = 28/5$). (**J**) Representative immunoblots show the expression of AnxA5 in WT and AnxA5-KO cells transfected either with an empty plasmid (in WT and AnxA5-KO cells) or with AnxA5, AnxA5-2mt, AnxA5-3mt, and AnxA5-5mt (in AnxA5-KO cells). Uncropped blots are provided in the Source Data. Significant differences were assessed using either one-way ANOVA with Tukey's multiple comparison tests or Kruskal–Wallis test (ns: not significant) and with the two-tailed unpaired Student's t-test or Kolmogorov–Smirnov ($*p < 0.05$, $**p < 0.01$, $***p < 0.001$, $****p < 0.0001$, and ns: not significant). Source data are available online for this figure.

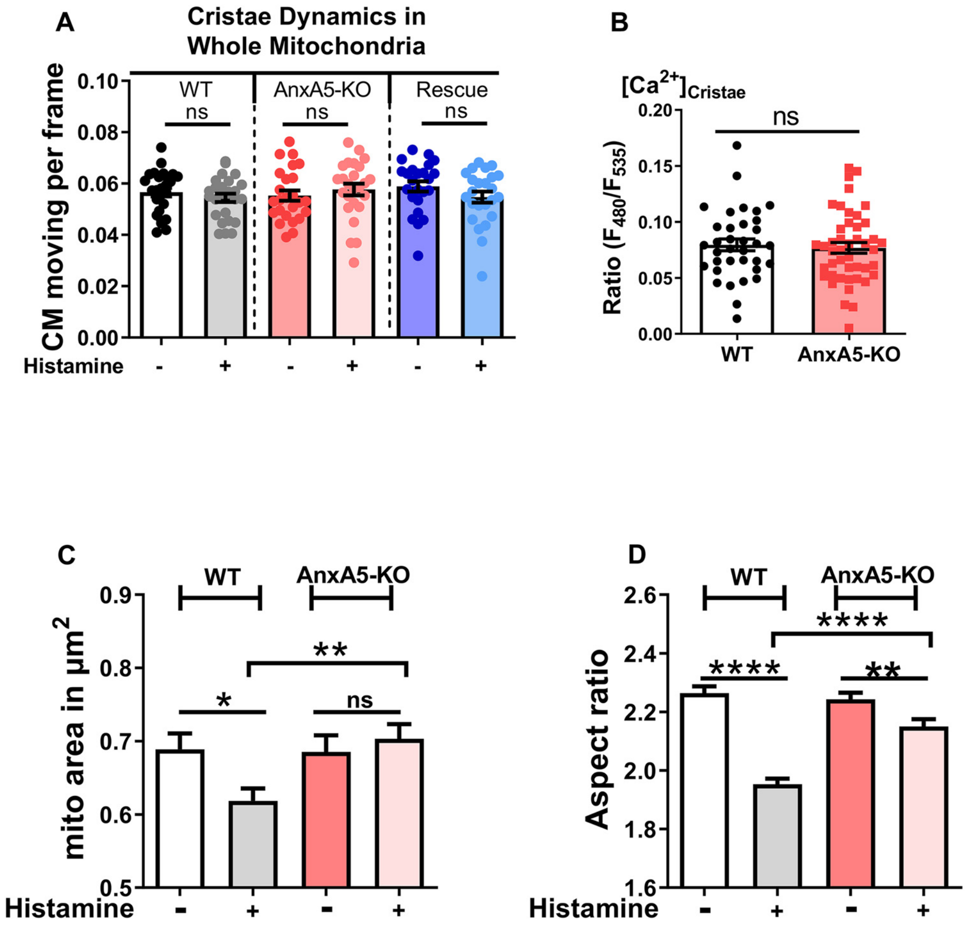

Figure EV3. AnxA5 contributes to Ca^{2+}-induced remodeling of mitochondrial morphology.

(**A**) Bar graph shows cristae membrane movements per frame in the whole mitochondria under basal and upon IP_3-induced $[Ca^{2+}]_{ER}$ release in WT (black), AnxA5-KO (red), and Rescue (blue) in HeLa cells. Data points represent the mean ± SEM ($n_{WT} = 26/6$; $n_{AnxA5\text{-}KO} = 25/6$; $n_{Rescue} = 24/6$). (**B**) Bar graph shows the basal $[Ca^{2+}]_{cristae}$ levels in WT (black) and AnxA5-KO (red) cells. Data points represent the mean ± SEM ($n_{WT} = 35/6$; $n_{AnxA5\text{-}KO} = 44/6$). (**C**) Bar graphs show the mitochondrial area and (**D**) aspect ratio in WT and AnxA5-KO before and 90 s after $[Ca^{2+}]_{ER}$ release. Data points represent the mean ± SEM ($n_{WT} = 84/9$; $n_{AnxA5\text{-}KO} = 88/9$). The *p*-values, from left to right, are for (**C**): $p = 0.0109$ ($*p < 0.05$), $p = 0.0015$ ($**p < 0.01$) and $p = 0.5559$ (ns); and for (**D**): $p < 0.0001$ ($****p < 0.0001$), $p < 0.0001$ ($****p < 0.0001$), and $p = 0.0066$ ($**p < 0.01$). Significant differences were assessed using either one-way ANOVA with Tukey's multiple comparison tests or the Kruskal-Wallis test and with the two-tailed unpaired Student's t-test ($*p < 0.05$, $**p < 0.01$, $****p < 0.0001$, and ns: not significant). Source data are available online for this figure.

 © The Author(s)

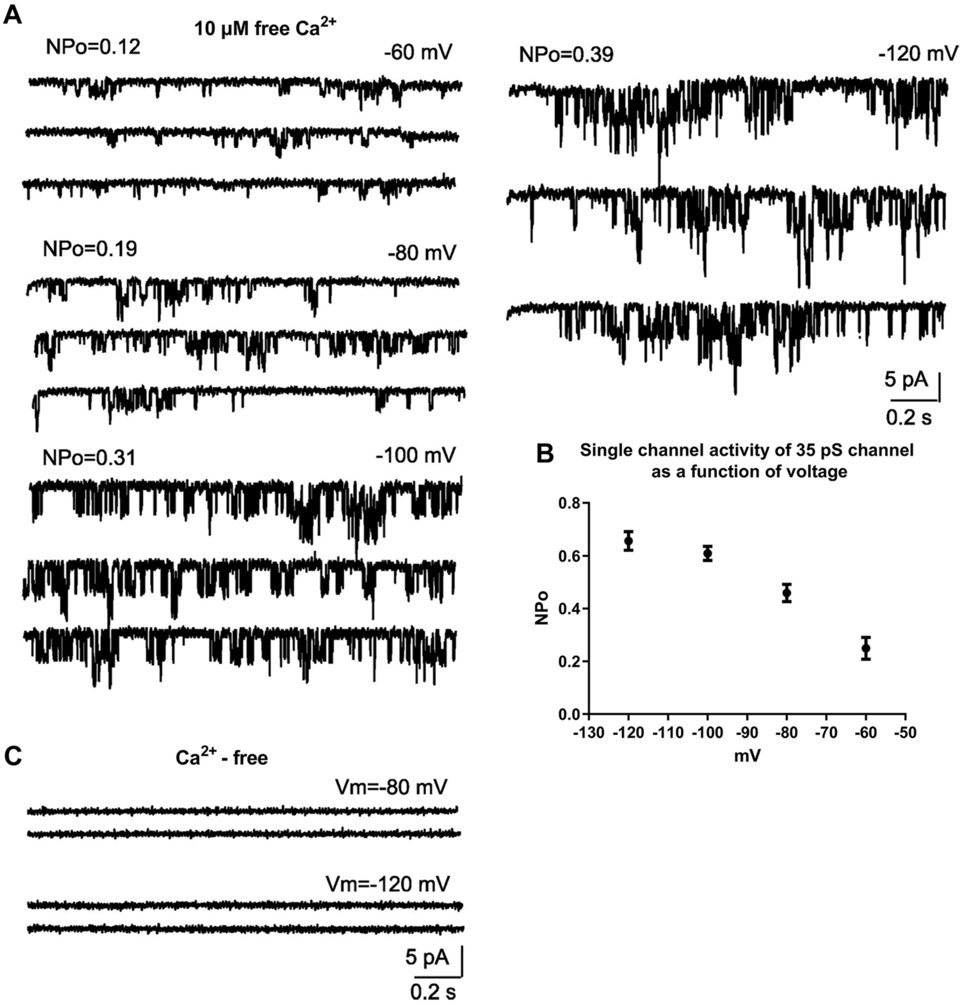

Figure EV4. Characterization of the 35 pS channel at the OMM.

(**A**) Representative single-channel traces showing the 35 pS channel in different voltages in intact mitochondria isolated from the WT HeLa cells (10 µM free Ca^{2+} in the pipette). (**B**) Graph shows the mean NPo of the 35 pS channel at −60, −80, −100, and −120 mV. (**C**) Representative single-channel traces showing no channel activity in intact mitochondria isolated from the WT HeLa cells (Ca^{2+}-free). Data points represent the mean ± SEM ($n_{WT\text{-}60} = 3$; $n_{WT\text{-}80} = 7$; $n_{WT\text{-}100} = 7$; $n_{WT\text{-}120} = 10$). Source data are available online for this figure.

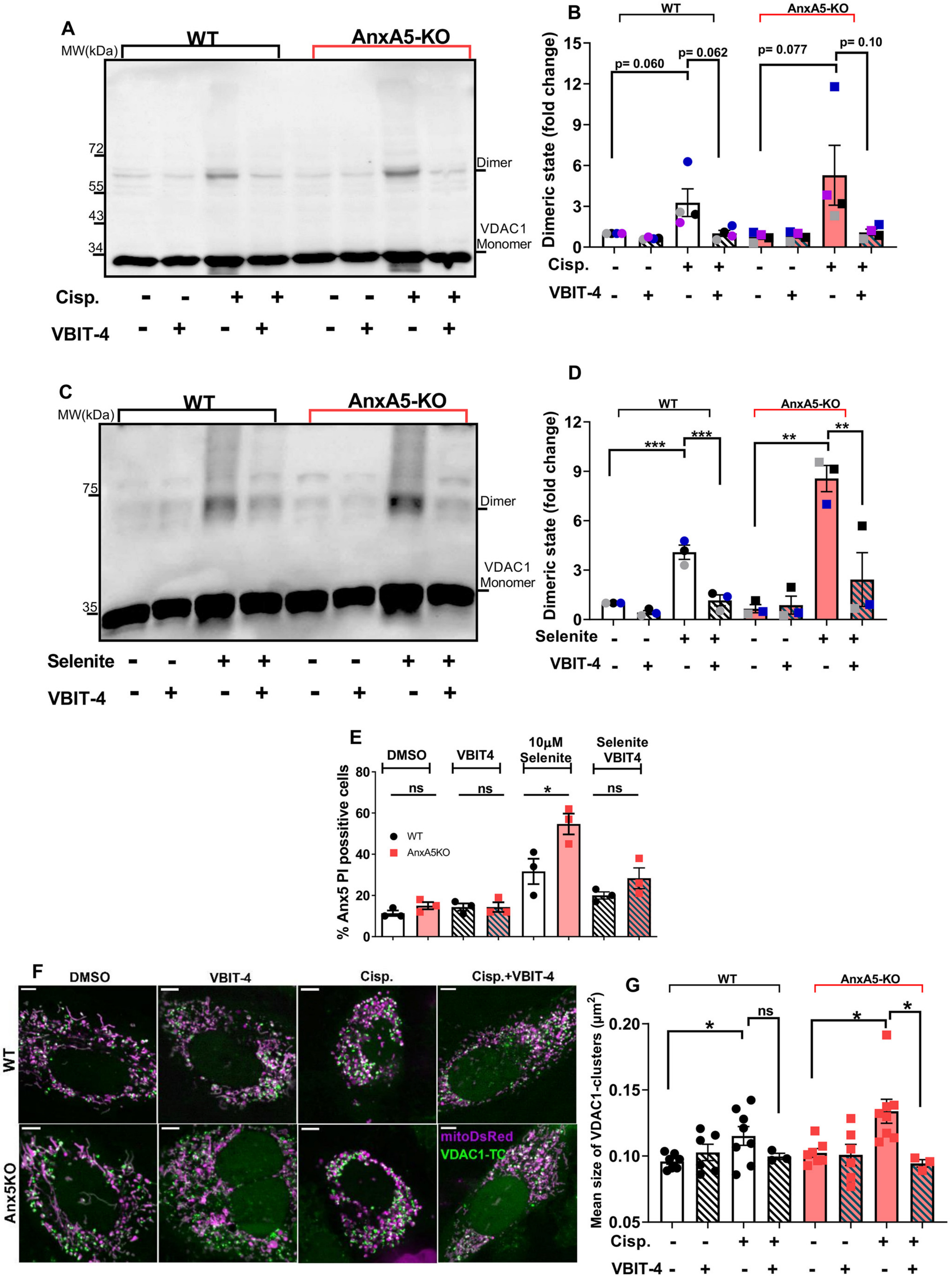
A
WT
AnxA5-KO
MW(kDa)
72
55
43
34
Dimer
VDAC1
Monomer
Cisp.
VBIT-4
B
WT
AnxA5-KO
p= 0.060
p= 0.062
p= 0.077
p= 0.10
Dimeric state (fold change)
C
WT
AnxA5-KO
MW(kDa)
75
35
Dimer
VDAC1
Monomer
Selenite
VBIT-4
D
WT
AnxA5-KO
***
**
Dimeric state (fold change)
Selenite
VBIT-4
E
DMSO
VBIT4
10μM Selenite
Selenite VBIT4
ns
*
WT
AnxA5KO
% Anx5 PI positive cells
F
DMSO
VBIT-4
Cisp.
Cisp.+VBIT-4
WT
Anx5KO
mitoDsRed
VDAC1-TC
G
WT
AnxA5-KO
ns
*
Mean size of VDAC1-clusters (μm²)
Cisp.
VBIT-4

 © The Author(s)

Figure EV5. AnxA5 regulates cisplatin/selenite-induced VDAC1 dimerization.

(**A**) Representative immunoblot shows monomeric and dimeric VDAC1 levels. Uncropped blots are provided in the Source data. (**B**) Bar graph shows the quantification of the immunoblot in WT (black) and AnxA5-KO (red) cells upon 24 h DMSO, 20 µM VBIT-4, 10 µM cisplatin, and cisplatin+VBIT-4 treatment (each color represents the experiments from the same day). Data points represent the mean ± SEM ($n_{WT\text{-}all} = 4$; $n_{AnxA5\text{-}KO\text{-}all} = 4$). (**C**) Representative immunoblot shows monomeric and dimeric VDAC1 levels in response to 48-hour DMSO, 20 µM VBIT-4, 10 µM selenite, and selenite + VBIT-4 treatment. (**D**) Bar graph shows the quantification of the immunoblot in panel (**C**). Data points represent the mean ± SEM (nWT-all = 3; nAnxA5-KO-all = 3). The *p*-values, from left to right, are: $p = 0.0002$ (***$p < 0.001$), $p = 0.0003$ (***$p < 0.001$), $p = 0.0017$ (**$p < 0.01$), and $p = 0.0082$ (**$p < 0.01$). (**E**) Bar graphs show the percentage of late apoptosis in WT (black) and AnxA5-KO (red) cells upon 48 h DMSO, 20 µM VBIT-4, 10 µM selenite, and selenite+VBIT-4 treatment. Data points represent the mean ± SEM (nWT-all = 3; nAnxA5-KO-all = 3). The *p*-values, from left to right, are: $p = 0.1688$ (ns), $p > 0.9999$ (ns), $p = 0.0448$ (*$p < 0.05$), and $p = 0.1932$ (ns). (**F**) Representative confocal images of WT and AnxA5-KO HeLa cells, expressing VDAC1-TC (green) and mitoDsRed (red), were captured (Scale bar = 5 µm). (**G**) Bar graph shows the quantification of the obtained confocal images indicating VDAC1 cluster size in µm^2 in WT (black) and AnxA5-KO (red) cells upon 12 h DMSO, 20 µM VBIT-4, 10 µM cisplatin, and cisplatin+VBIT-4 treatment. Data points represent the mean ± SEM ($n_{WT\text{-}DMSO} = 7$; $n_{WT\text{-}VBIT\text{-}4} = 6$; $n_{WT\text{-}cisp.} = 8$; $n_{WT\text{-}cisp.+VBIT\text{-}4} = 3$; $n_{AnxA5\text{-}KO\text{-}DMSO} = 7$; $n_{AnxA5\text{-}KO\text{-}VBIT\text{-}4} = 6$; $n_{AnxA5\text{-}KO\text{-}cisp.} = 8$; $n_{AnxA5\text{-}KO\text{-}cisp.+VBIT\text{-}4} = 3$). The *p*-values, from left to right, are: $p = 0.0494$ (*$p < 0.05$), $p = 0.2773$ (ns), $p = 0.0126$ (*$p < 0.05$), and $p = 0.0168$ (*$p < 0.05$). Significant differences were assessed using one-way ANOVA with Tukey's multiple comparison tests or with the unpaired Student's t-test (*$p < 0.05$, **$p < 0.01$, ***$p < 0.005$, and ns: not significant). Source data are available online for this figure.

© The Author(s)
